# Heptamethylindenyl (Ind*) enables diastereoselective benzamidation of cyclopropenes *via* Rh(iii)-catalyzed C–H activation†
†Electronic supplementary information (ESI) available: Experimental procedures and compound characterization. CCDC 1472771. For ESI and crystallographic data in CIF or other electronic format see DOI: 10.1039/c6sc02587k



**DOI:** 10.1039/c6sc02587k

**Published:** 2016-09-23

**Authors:** Natthawat Semakul, Kelvin E. Jackson, Robert S. Paton, Tomislav Rovis

**Affiliations:** a Department of Chemistry , Colorado State University , Fort Collins , Colorado 80523 , USA; b Chemistry Research Laboratory , University of Oxford , Mansfield Road , Oxford OX1 3TA , UK . Email: robert.paton@chem.ox.ac.uk

## Abstract

The diastereoselective coupling of *O*-substituted arylhydroxamates and cyclopropenes mediated by Rh(iii) catalysis was successfully developed. Through ligand development, the diastereoselectivity of this reaction was improved using a heptamethylindenyl (Ind*) ligand, which has been rationalized using quantum chemical calculations. In addition, the nature of the *O*-substituted ester of benzhydroxamic acid proved important for high diastereoselectivity. This transformation tolerates a variety of benzamides and cyclopropenes that furnish cyclopropa[*c*]dihydroisoquinolones with high diastereocontrol, which could then be easily transformed into synthetically useful building blocks for pharmaceuticals and bio-active molecules.

## Abstract 2

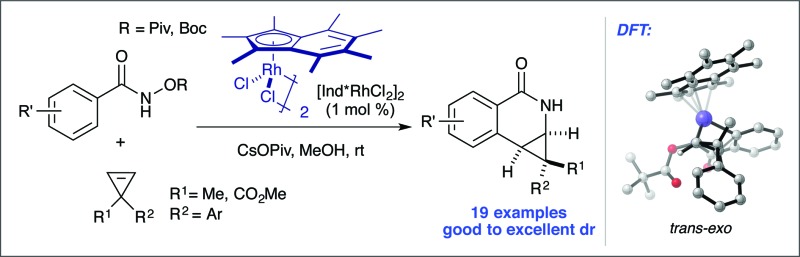
The diastereoselective coupling of *O*-substituted arylhydroxamates and cyclopropenes mediated by Rh(iii) catalysis was successfully developed.

## Introduction

**Fig. 1:**
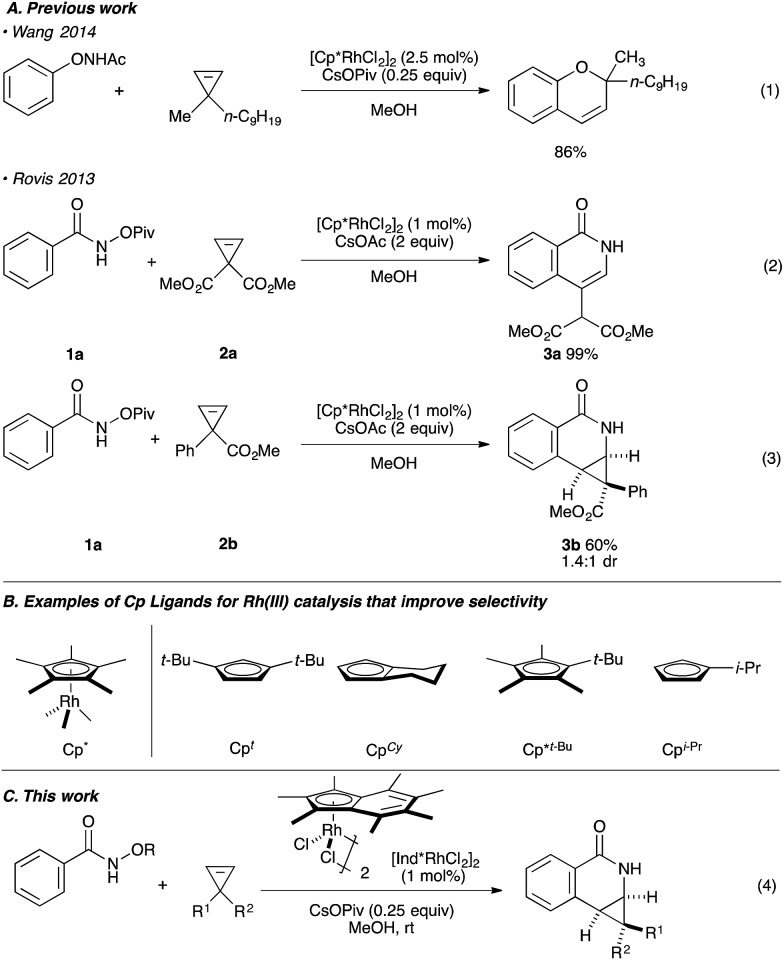
(A) The use of cyclopropenes in Rh(iii) catalysis. (B) Examples of Cp ligands that improve selectivity. (C) This work.

Rh(iii)-catalyzed C–H bond functionalization strategies have emerged as a powerful synthetic tool.^1^ The methodology allows for the functionalization of simple organic molecules and expedient synthesis of nitrogen-containing heterocycles from readily available precursors. Cyclopropenes constitute a class of building blocks with a special reactivity due to their high ring strain energy (54 kcal mol^–1^). In this context, a handful of reactions utilizing transition metals has been developed for the stereoselective functionalization of cyclopropenes.^2^ Under the aegis of Rh(iii) catalysis, Wang and coworkers have shown that cyclopropenes participate in a Rh(iii) catalyzed reaction with *N*-phenoxyacetamide to give 2*H*-chromenes (Fig. 1A, eqn (1)).^3^ Our group reported the Rh(iii)-mediated coupling of *O*-pivaloyl benzhydroxamate **1a** with 3,3-diester substituted cyclopropene **2a** to afford 4-substituted isoquinolone **3a** after ring opening of the three-membered ring (Fig. 1A, eqn (2)).^4^ During this study, when using the methyl 1-phenylcycloprop-2-ene-1-carboxylate **2b** as a substrate, the Cp*Rh(iii) catalyst gives the [4.1.0] bicyclic product **3b** in low diastereoselectivity (1.4 : 1 dr, Fig. 1A, eqn (3)). We believe the lack of diastereoselectivity stems from the poor facial selectivity during coordination of the cyclopropene and the migratory insertion step of cyclopropene unit. We reasoned that creating anisotropy around the cyclopentadienyl ligand of the rhodium metal center could solve this selectivity issue.

Our group^6–12^ and others^5,13^ have developed several Rh(iii)-catalyzed transformations where the nature of the Cp ligand drastically impacts the reactivity^5–7^ and selectivity^8–13^ of the reaction (Fig. 1B). For example, the sterically bulky di-*tert*-butylcyclopentadienyl (Cp^*t*^) ligand has been shown to improve the regiochemistry of alkyne and alkene insertion events in the synthesis of pyridones,^8^ pyridines^9^ and dihydroisoquinolones.^10^ Interestingly, Cramer and coworkers found a divergent regioselective synthesis of 3- and 4-substituted dihydroisoquinolone from *O*-Boc arylhydroxamate and styrene when using cyclohexane-fused cyclopentadienyl (Cp^Cy^) and pentamethylcyclopentadienyl (Cp*) ligands.^13^ Recently, our group disclosed a cyclopropanation reaction with the coupling of *N*-enoxyphthalimides and alkenes. Monoisopropylcyclopentadienyl (Cp^iPr^) outperforms the more common Cp* ligand, furnishing the *trans*-cyclopropane in high diastereoselectivity.^11^ Alternatively, a divergent carboamination path was identified when using a hindered *tert*-butyltetramethylcyclopentadienyl (Cp*^t-Bu^) ligand delivering the acyclic adduct with high chemoselectivity.^12^ Motivated by these results, we believed ligand design could provide a solution to the inherent selectivity issues encountered for the coupling of benzamide and 3,3-disubstituted cyclopropenes (Fig. 1C, eqn (4)).

## Results and discussion

We began our investigation by employing *O*-pivaloyl benzhydroxamate ester **1a** and cyclopropene **2c** as model substrates for the optimization of the catalytic process (Table 1). [Cp*RhCl_2_]_2_ provides the desired product in a moderate yield and diastereoselectivity (5.8 : 1 dr, entry 1). The relative stereochemistry of the major diastereoisomer of **3c** was confirmed by NOESY (see ESI†). By modulating the steric and electronic properties of the Cp ligand, we have shown that the diastereoselectivity of the reaction is considerably affected. Sterically hindered di-*tert*-butylcyclopentadienyl^8–10^ (Cp^*t*^) and the electron-poor trifluoromethyl tetramethylcyclopentadienyl^7^ (Cp*^CF_3_^) ligands give only modest diastereoselectivity (entries 2 and 3). The monoisopropylcyclopentadienyl ligand^11^ (Cp^iPr^) gave the desired product in a good yield albeit with no diastereocontrol (entry 4). 3,5-Bis(trifluoromethyl)aryl tetramethylcyclopentadienyl^6^ (Cp*^bisCF_3_Ar^) provides the desired product in good yield with slightly improved diastereoselectivity (7.0 : 1 dr, entry 5). Good level of diastereocontrol (8.8 : 1 dr) is achieved when *tert*-butyl tetramethylcyclopentadienyl^12^ (Cp*^tBu^) ligand was employed (entry 6). Gratifyingly, heptamethylindenyl ligand^14^ (Ind*) provides high reactivity and diastereoselectivity with 90% yield and 15.2 : 1 dr (entry 7). To demonstrate the scalability of the transformation, the reaction was performed in 2 mmol scale of substrate **1a**, which gives the expected product with comparable yield and selectivity. The catalyst loading can be lowered to 0.5 mol% [Ind*RhCl_2_]_2_ without affecting reactivity (entry 8). We then examined the nature of the directing group. It was found that using *O*-Boc benzhydroxamate ester **1b** as a substrate gave excellent diastereoselectivity (>20 : 1 dr) but with slightly lower yield (entry 9), presumably due to a competitive Lossen rearrangement under the basic conditions.^15^

**Table 1:** Ligand optimization^*a*^

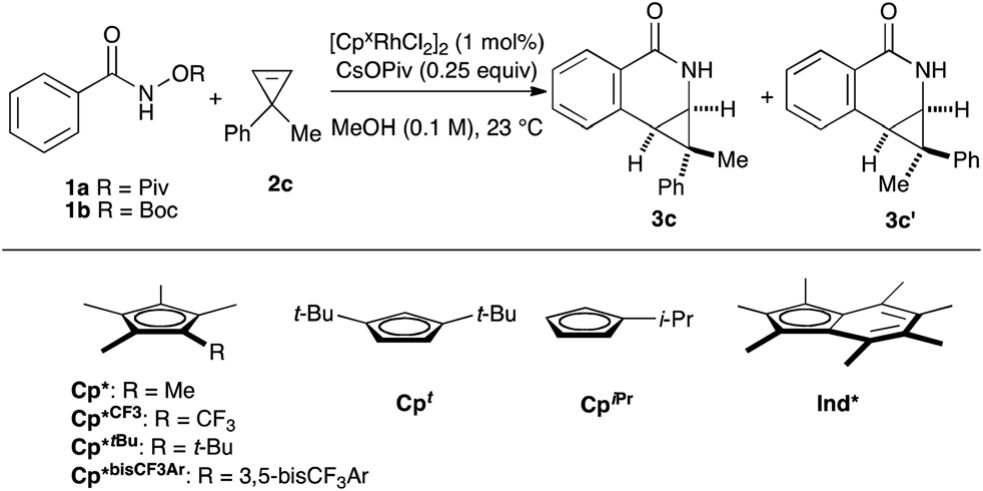				
Entry	Substrate	Cp^x^	Yield^*b*^	dr (**3c** : **3c′**)^*b*^
1	**1a**	Cp*	63	5.8 : 1
2	**1a**	Cp^*t*^	82	5.0 : 1
3	**1a**	Cp*^CF_3_^	75	5.3 : 1
4	**1a**	Cp^iPr^	73	1.1 : 1
5	**1a**	Cp*^bisCF_3_Ar^	80	7.0 : 1
6	**1a**	Cp*^tBu^	64	8.8 : 1
7	**1a**	Ind*	90^*c*^	15.2 : 1
8^*d*^	**1a**	Ind*	85^*c*^	15 : 1
9	**1b**	Ind*	69^*c*^	>20 : 1

^*a*^Reaction conditions: **1a** or **1b** (0.1 mmol), **2c** (0.11 mmol), Rh catalyst (1 mol%), CsOPiv (0.25 equiv.) in MeOH (0.1 M) at 23 °C for 18 h.

^*b*^The yield and diastereoselectivity were measured from the ^1^H-NMR analysis of the unpurified reaction mixture using 1,3,5-trimethoxybenzene as an internal standard.

^*c*^Isolated yield.

^*d*^Catalyst loading of 0.5 mol% on 1 mmol scale.

Both benzamide directing groups, *O*-Piv **1a** (condition **A**) and *O*-Boc **1b** (condition **B**), were used for studying the scope of the transformation (Table 2). Substituents at the *para* position of the benzamide are tolerated in the reaction (Table 2, **3d–3h**). The *O*-Piv directing group with an electron rich *para*-methoxy substituent (OMe) gave excellent diastereoselectivity (>20 : 1 dr, product **3e**) compared to electron deficient substituents (∼10 : 1 dr, products **3f**, **3g** and **3h**). The electron-rich benzamide derived from gallic acid furnishes the desired product with good yield and excellent diastereoselectivity (>20 : 1 dr, product **3i**). The *O*-Boc directing group gives the products in good to excellent diastereoselectivity (**3d–3g**). Of interest are halogen substituents at the *para* positions (Cl and Br) which provide a functional group handle for further chemical modification. The *ortho*-methyl arylbenzhydroxamate substrate retards the transformation presumably due to steric hindrance. Substituents at the *meta* position on the arylhydroxamates can potentially deliver two regioisomeric products arising from the selectivity of C–H activation. *meta*-Trifluoromethyl arylhydroxamate exclusively provides the 6-substituted product (**3j**) in good yield and diastereoselectivity. Tetrahydronaphthalene-derived arylhydroxamate underwent the transformation with good regioselective C–H activation at less hindered position (8.6 : 1 ratio) to give the desired product (**3k**) in good yield and high diastereoselectivity. However, *meta*-methyl arylhydroxamate gave ∼3.6 : 1 regioisomeric ratio of C–H activation in good yield and diastereoselectivity (**3l**). *meta*-Methoxy arylhydroxamate provided 1 : 1 mixture of regioisomeric products (**3m** and **3m′**) in good diastereocontrol, presumably a consequence of a combination of steric effects and kinetic acidity issues. In addition, X-ray structure of **3m** ambiguously confirmed the relative stereochemistry of *trans*-diastereomer.

**Table 2:** Benzamide scope^*a*^ ^,^ ^*b*^ ^,^ ^*c*^

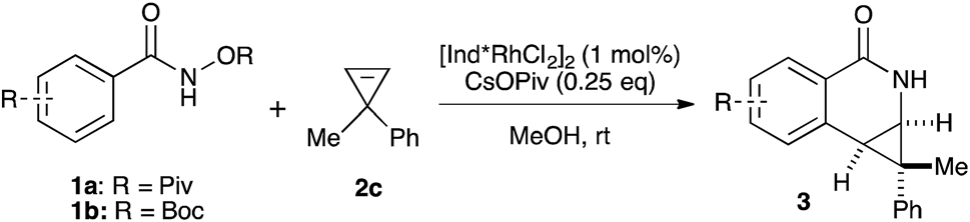
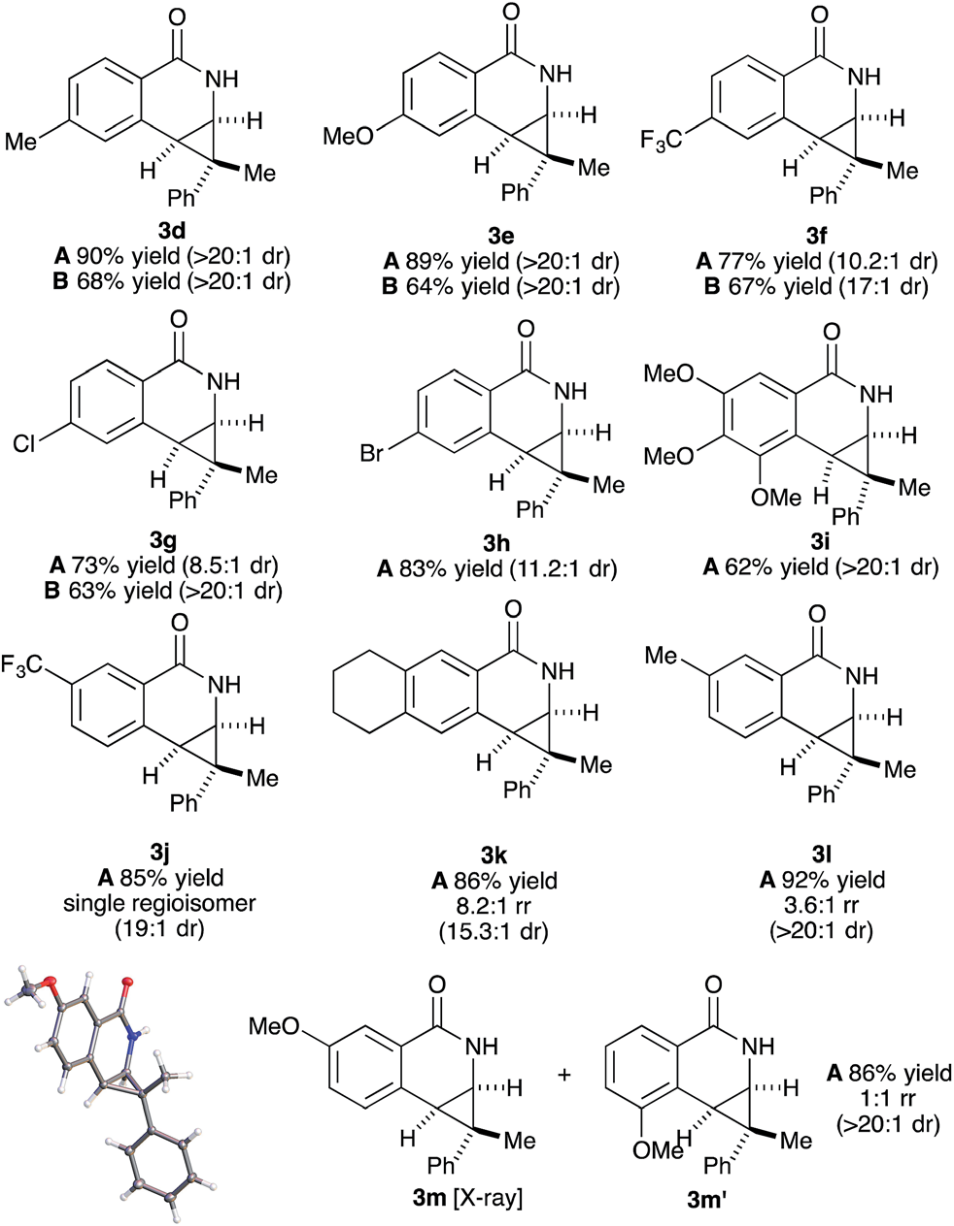

^*a*^Conditions: **1a** (for **A**) or **1b** (for **B**) (0.1 mmol), **2c** (0.11 mmol), Rh catalyst (1 mol%), CsOPiv (0.25 equiv.) in MeOH (0.1 M) at 23 °C for 18 h.

^*b*^Isolated yield of the major diastereomer after silica gel column chromatography.

^*c*^Diastereoselectivity was measured by ^1^H-NMR spectra of the unpurified material.

Variations of the cyclopropene coupling partner were explored for the transformation using the *O*-Boc benzhydroxamate **1b**. Cyclopropenes bearing substituents at the para position gave the desired products in moderate yields and excellent diastereoselectivity regardless of the electronic nature of substituents (Table 3, **4a**, **4b** and **4c**). Cyclopropene with a *meta*-methoxy group undergoes the transformation with slightly lower diastereoselectivity relative to the *para*-methoxy group (Table 3, **4d**). A naphthalene-substituted cyclopropene **2e** and a spiro-tetralin containing substrate **2f** each furnish the desired products **4e** and **4f** in good yield and excellent diastereoselectivity. In our previous studies,^4^ we found that methyl 1-phenylcycloprop-2-ene-1-carboxylate **2b** reacts with benzamide **1b** and gives the desired product with low diastereoselectivity (1.4 : 1 dr) using [Cp*RhCl_2_]_2_ as the precatalyst. With the [Ind*RhCl_2_]_2_ ligand, we were pleased to find that cyclopropene **2b** afforded the dihydroisoquinolone **4g** with improved diastereoselectivity (8.7 : 1 dr). The relative stereochemistry of the major diastereomer of **4g** was confirmed by NOESY (see ESI†). The observed major diastereomer can be rationalized by the size of the substituents on the cyclopropane ring. Thus, the phenyl group is larger than the carboxylate ester (*A*-values for Ph- and –CO_2_Me are 3.0 and 1.3, respectively) leading to higher diastereoselectivity observed in these reactions. The amidoarylation with benzyl substituted cyclopropene affords the desired product **4h** in good yield but with lower diastereoselectivity. This observation can be explained by the steric differences of phenyl *vs.* benzyl groups (*A*-values for Ph and Bn are 3.0 and 1.75, respectively). 2,3,3-Trisubstituted cyclopropenes did not participate in the Rh(iii)-catalyzed coupling with benzamides.

**Table 3:** Cyclopropene scope^*a*^

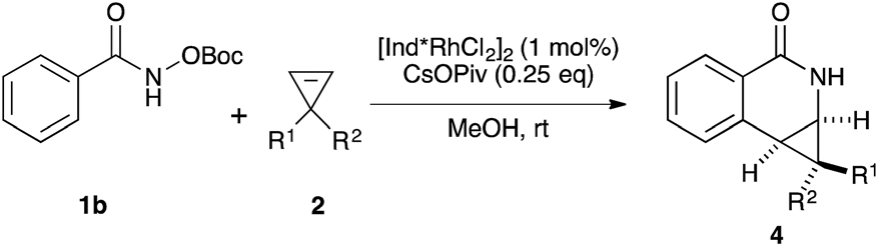
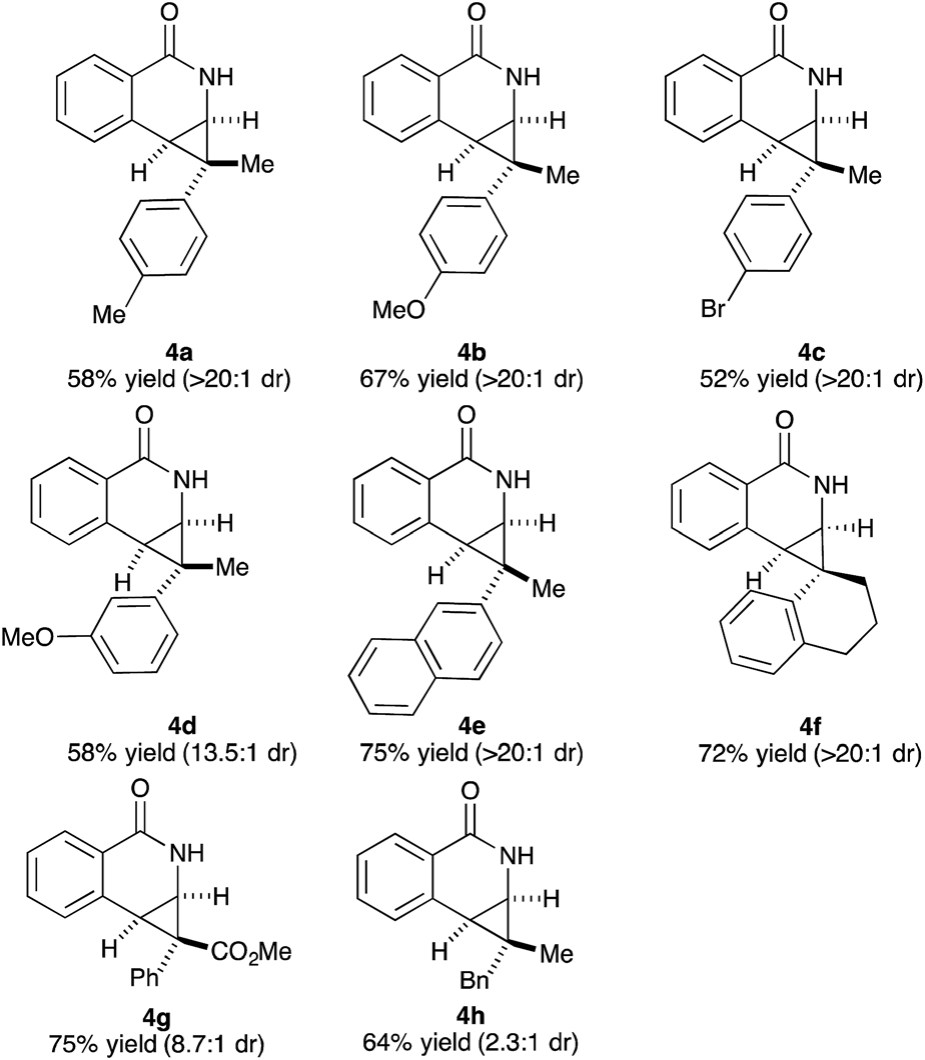

^*a*^See Table 2.

**Scheme 1:**
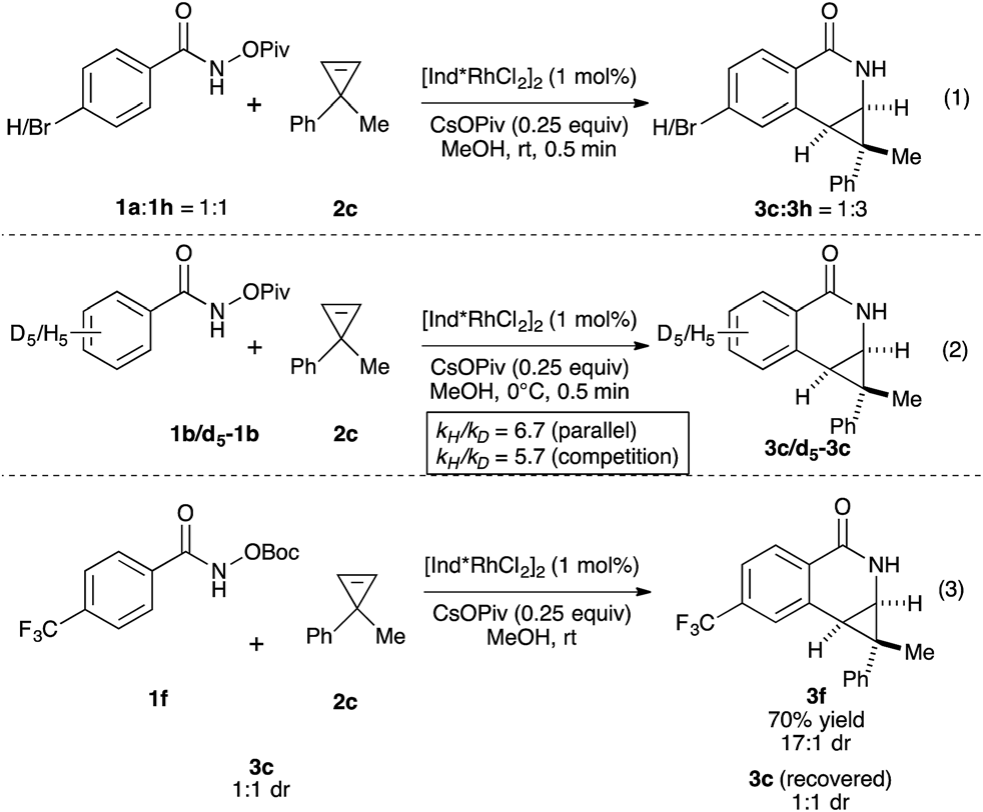
Mechanistic experiments.

We then investigated the mechanism of the transformation. The reversibility of C–H activation was first examined. Trace deuterium incorporation (<5%) was observed when the reaction was run in CD_3_OD, suggesting the C–H activation is largely irreversible (see ESI†). The competitive reaction between *p*-bromobenzamide (**1h**) and unsubstituted benzamide (**1a**) was conducted to probe the electronic preference of reaction (Scheme 1, eqn (1)). The product formation favors an electron deficient substrate in a 3 : 1 ratio. Kinetic isotope studies revealed KIE values of 6.7 and 5.7 for the parallel and competition experiments, respectively (Scheme 1, eqn (2)). These studies together suggest that the C–H activation occurs *via* concerted metallation-deprotonation (CMD) mechanism and is the turnover-limiting step, as seen in several previous examples of C–H activation with Rh(iii).^16,17^ To determine if epimerization of the product occurs under the reaction conditions, we independently prepared product **3c** (1 : 1 dr) and resubjected it to the reaction conditions of benzamide **1f** and cyclopropene **2c**. After full conversion to **3f** (70% yield, 17 : 1 dr), we did not observe any change of the dr of **3c**, indicating the products are not epimerized under the reaction conditions.

**Scheme 2:**
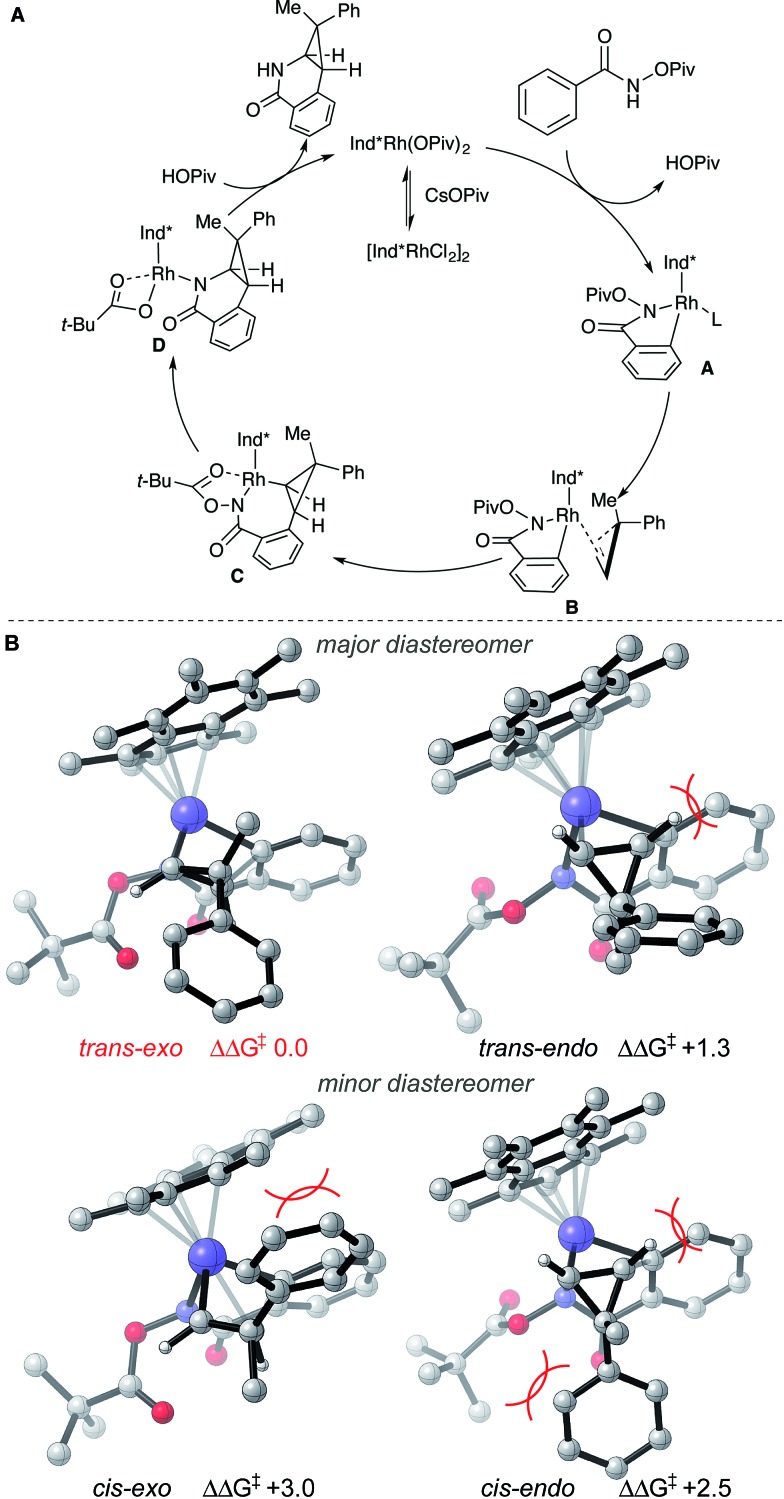
(A) Proposed reaction mechanism and (B) stereochemical model for diastereoselectivity. Gibbs energies in kcal mol^–1^.

Based on literature precedent^17^ and our mechanistic studies, the mechanism of the transformation is proposed in Scheme 2A. The Ind*Rh(OPiv)_2_ species is generated *in situ* by an anion exchange of [Ind*RhCl_2_]_2_ and CsOPiv. The amide directed C–H activation occurs *via* a CMD mechanism to give the five-membered rhodacycle intermediate **A**, which then coordinates the cyclopropene giving intermediate **B**.

To understand diastereoselectivity and the effect of the Ind* ligand we performed density functional theory (DFT) calculations.^18^ Transition structures (TSs) were optimized at the TPSS/def2-TZVP level of theory, which was the most accurate of several functionals tested (see ESI†), for the reaction of benzamide **1a** with cyclopropene **2c** using Cp* and Ind* ligands. Firstly, we confirmed that the product diastereoselectivity arises from the facial selectivity of the coordination of the cyclopropene and subsequent migratory insertion step (Scheme 2B). Our calculations indicate a facile migratory insertion step (barriers of 8.0 and 9.7 kcal mol^–1^) which is substantially exergonic, so that the barriers in the reverse direction are prohibitively large (25.7 kcal mol^–1^) given the reaction conditions. We predict this step will occur irreversibly,^19^ thus determining the diastereoselectivity. With both Cp* and Ind* ligands, we found that the insertion step can proceed *via* four distinct TSs. For both diastereofaces of the cyclopropene, two conformers exist in which the cyclpropenyl gem-disubstituted carbon can be oriented towards (*endo*) or away (*exo*) from the benzamide. In terms of the nomenclature adopted the *trans*-diastereomer is the major product experimentally. All four possibilities are shown for the Ind* ligand in Scheme 2B (with Cp* structures in the ESI†).

The most favorable TS (*trans*–*exo*) agrees with the observed sense of diastereoselectivity and the computations also quantitatively reproduce the increase in selectivity of Ind* *vs.* Cp* ligands (*cis*–*trans* ΔΔ*G* ^‡^ increasing from 1.8 to 2.5 kcal mol^–1^; note that the favored *cis* TS changes from *cis*–*exo* for Cp* to *cis*–*endo* for Ind*). In the favored TS the cyclopropene substrate is oriented with the methyl group towards the ligand. The alternative approach (*trans*–*endo*) is less favorable, suffering from a more severe H…H clash (2.16 Å) about the incipient C–C bond. The Piv group is also oriented towards the ligand in this less favorable TS. The minor diastereomer results from trying to orient the larger phenyl group toward the Ind* ligand (*cis*–*exo* TS) or toward the substrate and directing group (*cis*–*endo* TS), causing unfavorable steric interactions. These structures show the synergistic effect of steric interactions involving both the Ind* ligand and directing group on the facial selectivity. Migratory insertion of cyclopropene gives intermediate **C**. Reductive elimination (C–N bond formation) occurs to generate a Rh(i) species.^19^ The saturated coordination of acyl directing group to Rh(iii) of intermediate **C** is important for the reductive elimination step since *O*-methyl benzhydroxamate is not reactive for the transformation (see ESI†).

**Scheme 3:**
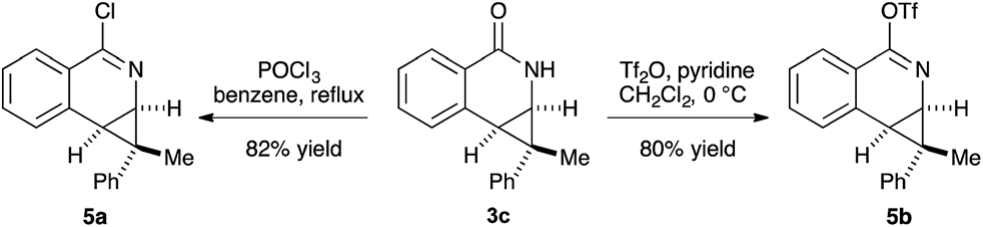
Derivatizations of product.

The prevalence of nitrogen-containing heterocycles in pharmaceuticals led us to investigate the derivatization of the dihydroisoquinolones bearing [4.1.0] bicycles.^20^ For example, the chloro- and *O*-triflate substituted dihydroisoquinolines, which are versatile functional group handles for further cross-coupling reactions could be easily prepared from the dihydroisoquinolone products in good yields, allowing for easy incorporation of these bicycles into pharmaceuticals or bio-active molecules (Scheme 3).

## Conclusions

In summary, we have developed a heptamethylindenyl (Ind*) ligand that enables high diastereoselectivity for cyclopropene insertion in the Rh(iii)-catalyzed synthesis of cyclopropa[*c*]dihydroisoquinolone. The steric interaction of the ligand on rhodium and the ester substitution of *O*-substituted benzhydroxamate work cooperatively to improve the diastereoselectivity of cyclopropene insertion. Mechanistically, the C–H activation proceeds *via* a concerted metallation-deprotonation pathway and is the turnover-limiting step. This methodology is useful for the rapid synthesis of nitrogen-containing heterocycles with a [4.1.0] motif and their derivatives.
