# Supplementary material for: Heptamethylindenyl (Ind*) enables diastereoselective benzamidation of cyclopropenes via Rh(iii)-catalyzed C–H activation
Source: Chem Sci. 2016 Sep 23;8(2):1015–20. doi: 10.1039/c6sc02587k (PMC5354047; doi:10.1039/c6sc02587k)
Supplement: Supplementary file 1 [file SC-008-C6SC02587K-s001.pdf]

Electronic Supplementary Material (ESI) for Chemical Science.
This journal is © The Royal Society of Chemistry 2016

Computational Supporting Information

Heptamethyl Indenyl (Ind*) Enables Diastereoselective Benzamidation of Cyclopropenes *via* Rh(III)-Catalyzed C-H Activation

Kelvin E. Jackson and Robert S. Paton*
Chemistry Research Laboratory, University of Oxford, Mansfield Road, Oxford OX1 3TA, UK

Table of Contents

1. Computational Methods S1
2. Analysis of the competing migratory insertion transition structures with the Cp* ligand S2
3. Analysis of the competing migratory insertion transition structures with the I* ligand S5
6. Product structures S7
7. Dispersion effects S8
8. Cartesian Coordinates S9

1. Computational Methods

Density functional theory (DFT) calculations were performed with *Gaussian09* rev D.01.[1] The meta-generalized gradient approximations (meta-GGA) TPSS exchange-correlation functional[2] was used for all geometry optimizations with density fitting for Coulomb integrals (RI-J), a fine integration grid and the Karlsruhe def2-TZVP basis set for all elements.[3] All stationary points were verified as either minima or saddle points by the presence of zero or a single imaginary harmonic vibrational frequency, respectively. To locate the most stable conformations of structures containing the unsymmetrical permethylindenyl ligand, a dihedral scan was performed in each case with a def2-SVP basis set. Stable conformations were reoptimized with the larger def2-TZVP basis set and the most stable structures are presented. Relative free energies were evaluated at 298.15 K applying a haptic translational entropy correction to adjust from a standard-state of 1 atm to 1 mol/l, while a quasi-harmonic approximation was used in which the treatment of vibrational entropies switches

[1] Gaussian 09, Revision D.01, M. J. Frisch, G. W. Trucks, H. B. Schlegel, G. E. Scuseria, M. A. Robb, J. R. Cheeseman, G. Scalmani, V. Barone, B. Mennucci, G. A. Petersson, H. Nakatsuji, M. Caricato, X. Li, H. P. Hratchian, A. F. Izmaylov, J. Bloino, G. Zheng, J. L. Sonnenberg, M. Hada, M. Ehara, K. Toyota, R. Fukuda, J. Hasegawa, M. Ishida, T. Nakajima, Y. Honda, O. Kitao, H. Nakai, T. Vreven, J. A. Montgomery, Jr., J. E. Peralta, F. Ogliaro, M. Bearpark, J. J. Heyd, E. Brothers, K. N. Kudin, V. N. Staroverov, R. Kobayashi, J. Normand, K. Raghavachari, A. Rendell, J. C. Burant, S. S. Iyengar, J. Tomasi, M. Cossi, N. Rega, J. M. Millam, M. Klene, J. E. Knox, J. B. Cross, V. Bakken, C. Adamo, J. Jaramillo, R. Gomperts, R. E. Stratmann, O. Yazyev, A. J. Austin, R. Cammi, C. Pomelli, J. W. Ochterski, R. L. Martin, K. Morokuma, V. G. Zakrzewski, G. A. Voth, P. Salvador, J. J. Dannenberg, S. Dapprich, A. D. Daniels, Ö. Farkas, J. B. Foresman, J. V. Ortiz, J. Cioslowski, and D. J. Fox, Gaussian, Inc., Wallingford CT, 2009.
[2] J. Tao, J. P. Perdew, V. N. Staroverov and G. E. Scuseria, *Phys. Rev. Lett.*, 2003, **91**, 146401.
[3] (a) A. Schaefer, H. Horn and R. Ahlrichs, *J. Chem. Phys.,* 1992, **97**, 2571; (b) A. Schaefer, C. Huber, and R. Ahlrichs, *J. Chem. Phys.,* 1994, **100**, 5829.

from a rigid-rotor harmonic oscillator to a free rotor at frequencies below 100 cm^{-1}, implemented in Python as we have described previously.[4] The effects of a correction for dispersion were evaluated by reoptimization of transition structures using the TPSS and PBE functionals[5] with and without a Becke-Johnson damped D3-correction,[6] and with the M06 hybrid meta GGA functional.[7] Analysis of ligand cone angles was performed with an in-house Python script using Bondi's van der Waals radii.[8] Visualization of non-covalent interactions was carried out with *NCIplot 3.0*, based on densities obtained from PBE/6-31G(d)/DGDZVP single point calculations.[9]

2. Analysis of the competing migratory insertion transition structures with the Cp* ligand

We studied the C-C bond forming step to assess the origins of diastereoselectivity and to understand the role of the Cp* or I* ligand in this step (Figure S1). Firstly, we confirmed that this migratory insertion step is stereo-determing, computing the barrier in the forward and reverse direction in each conformation. The calculations show facile insertion, and substantial exergonicity, making the barriers in the reverse direction prohibitively large (25.7 kcal/mol). This step is thus expected to occur irreversibly, determining the diastereoselectivity.

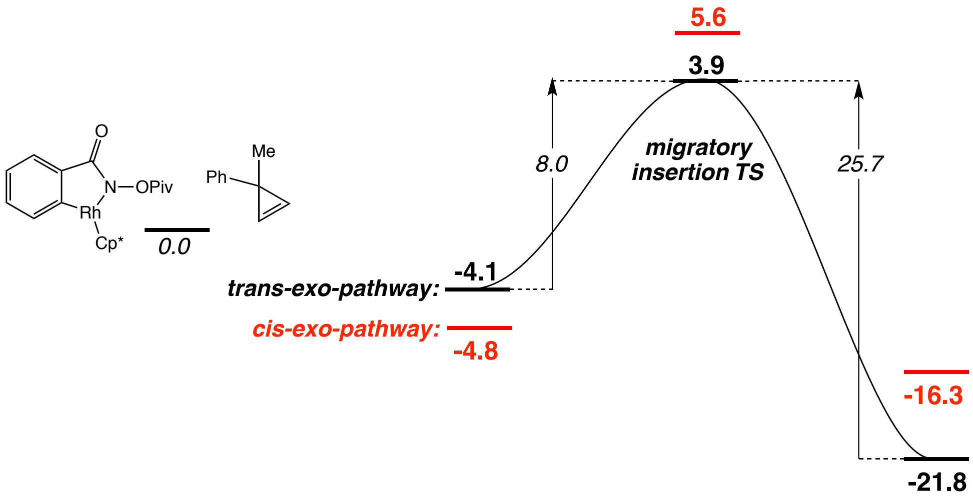

Figure S1. Cyclopropene insertion is computed to be irreversible (TPSS/def2-TZVP energeties computed relative to separated rhoadacycle and cyclopropene shown in kcal/mol).

---

[4] W. E. Cortopassi, R. Simion, C. E. Hornsby, T. C. Costa Franca and R. S. Paton, *Chem. Eur. J.,* 2015, **21**, 18983.

[5] (a) J. P. Perdew, K. Burke and M. Ernzerhof, *Phys. Rev. Lett.*, 1996, **77** 3865; (b) J. P. Perdew, K. Burke and M. Ernzerhof, *Phys. Rev. Lett.*, 1997, **78** 1396.

[6] S. Grimme, J. Antony, S. Ehrlich and H. Krieg, *J. Chem. Phys.,* 2010, **132**, 154104.

[7] Y. Zhao and D. G. Truhlar, *Theor. Chem. Acc.,* 2007, **120**, 215.

[8] A. Bondi, *J. Phys. Chem.,* 1964, **68**, 441.

[9] (a) J. Contreras-Garcia, E. Johnson, S. Keinan, R. Chaudret, J-P. Piquemal, D. Beratan, W. Yang, *J. Chem. Theor. Comp*. **2001**, *7*, 625; (b) E R. Johnson, S. Keinan, P. Mori-Sanchez, J. Contreras-Garcia, A. J. Cohen, and W. Yang, *J. Am. Chem. Soc*. **2010**, *132*, 6498.

Migratory insertion can proceed via four distinct transition structures (TSs), two for each of the diastereomeric products. These structures differ according to whether the gem-disubtituted carbon of the cyclopropene is oriented towards (*endo*) or away (*exo*) from the benzamide, and the relative *cis/trans* stereochemistry of the final product. Experimentally the *trans*-diastereomer dominates. All four possibilities are shown for the Cp* ligand in Figure S2. In the favored TS (*trans-exo*) the cyclopropene substrate is oriented with the methyl group towards the ligand. All of the TS structures show a greater metal-centroid distance *vs* intermediate **A** by 0.05Å or more, and in this preferred TS the metal-centroid distance is the longest. The minor diastereomer results from trying to orient the larger phenyl group toward the Cp* ligand (in the *cis-exo* TS) causing unfavorable steric interactions. The alternative *endo* structures avoid clashes with the ligand, but more severe interactions between the substrates result: a cyclopropene C-H is in close contact with a rhodacycle C-H (2.16Å) in both structures, and the phenyl group interacts unfavorably with the N-protecting group in the *trans-endo* structure. Another consequence of adopting the *endo* conformation is that the Boc group is oriented less favorably than in the *exo* structures, towards the ligand.

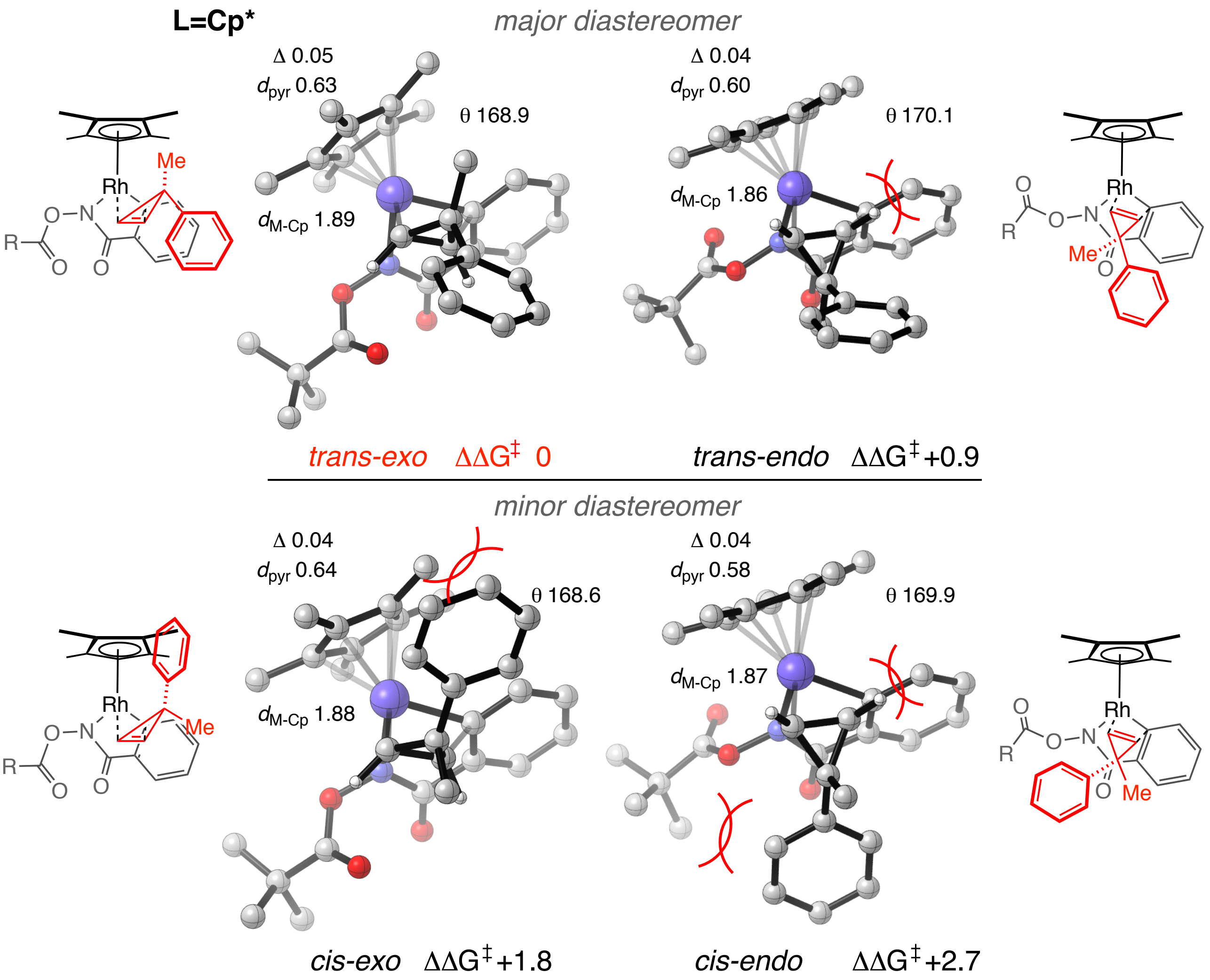

Figure S2. Comparison of TPSS/def2-TZVP optimized TSs for cyclopropene addition with Cp* ligands (relative TS Gibbs energies in kcal/mol). Metal-centroid distances and pyramidalization at Rh shown in Å. For reference, intermediate A is essentially planar with d_{pyr} = 0.03Å.

Analysis of the orientation of the Cp ligands (Figure S3) shows minor variation between the four TS structures of 10°.

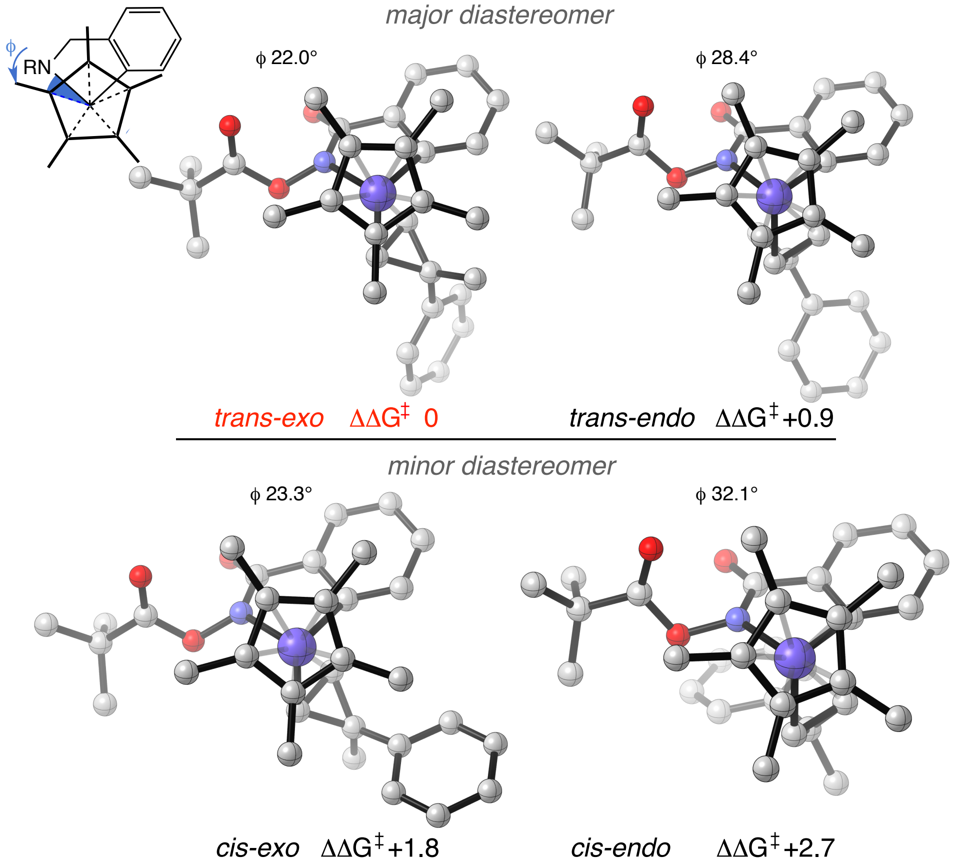

Figure S3. Alternative overhead perspective of TPSS/def2-TZVP optimized TSs for cyclopropene addition with Cp* ligands (relative TS Gibbs energies in kcal/mol).

3. Analysis of the competing migratory insertion transition structures with the I* ligand

We obtained migratory insertion TSs with the I* ligand as before. Due to the unsymmetrical nature of the ligand it was necessary to perform dihedral scans about the metal-centroid axis for each structure. The most stable conformer for each of the four possibilities is shown in Figure S4. In all but one structure, the most favorable geometry of the ligand involves orienting the benzene ring towards the incoming cyclopropene. The overhead perspective shown in Figure S5 highlights these geometries. In the most sterically demanding case (*cis-exo*) it is more favorable to orient this ring away. The same structure as for Cp*, *trans-exo,* is the most stable. In the preferred TS, the ligand to accommodates the methyl group to the extent that this interaction appears to be stabilizing (the NCI analysis is shown in Figure S6).

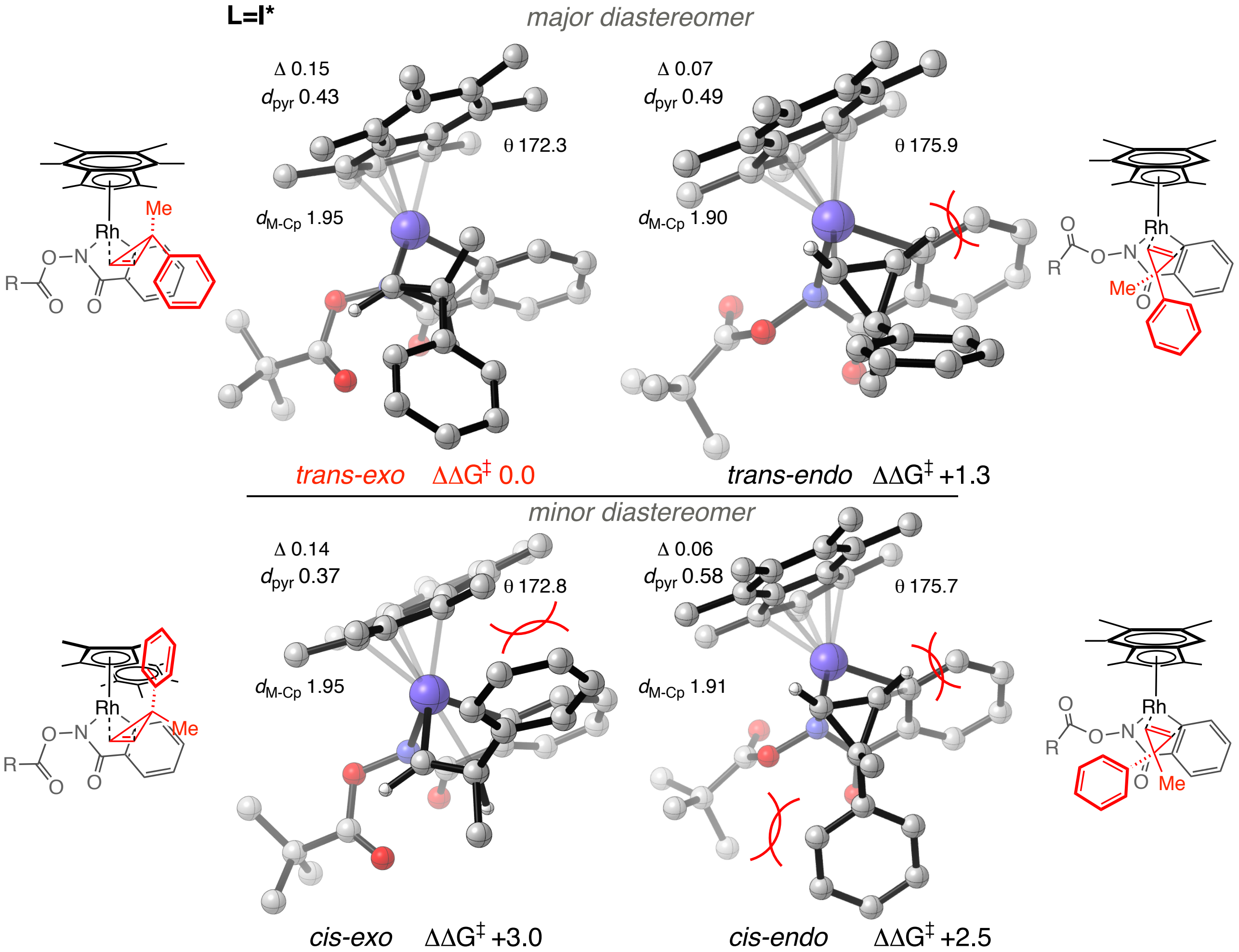

Figure S4. Comparison of TPSS/def2-TZVP optimized TSs for cyclopropene addition with I* ligands (relative TS Gibbs energies in kcal/mol). Metal-centroid distances and pyramidalization at Rh shown in Å.

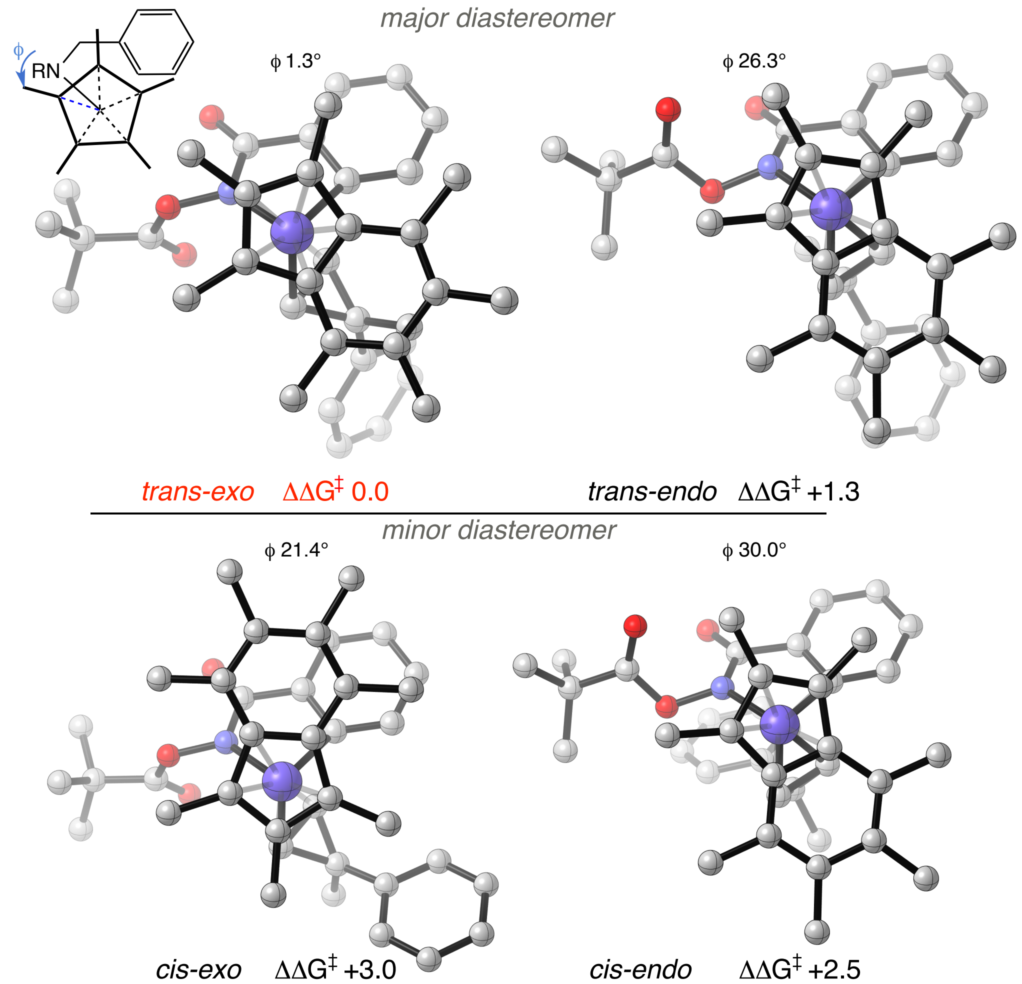

Figure S5. Alternative overhead perspective of TPSS/def2-TZVP optimized TSs for cyclopropene addition with I* ligands (relative TS Gibbs energies in kcal/mol).

The nonbonding interactions discussed above are evident from the visualization of the Non-Covalent Interaction isosurfaces (Figure S6). In the preferred TS structure (*exo-trans*) the interaction of the methyl group and the ligand does not display characteristics of an unfavorable steric interaction – rather both the distance between these groups, and the isosurface suggest a C-H-π interaction. The *endo-trans* structure is also shown for comparison. Here the methyl group is oriented to produce a close C-H contact with the benzamide, and there is an unavoidable clash between two C-H hydrogen atoms at site of the forming C-C bond.

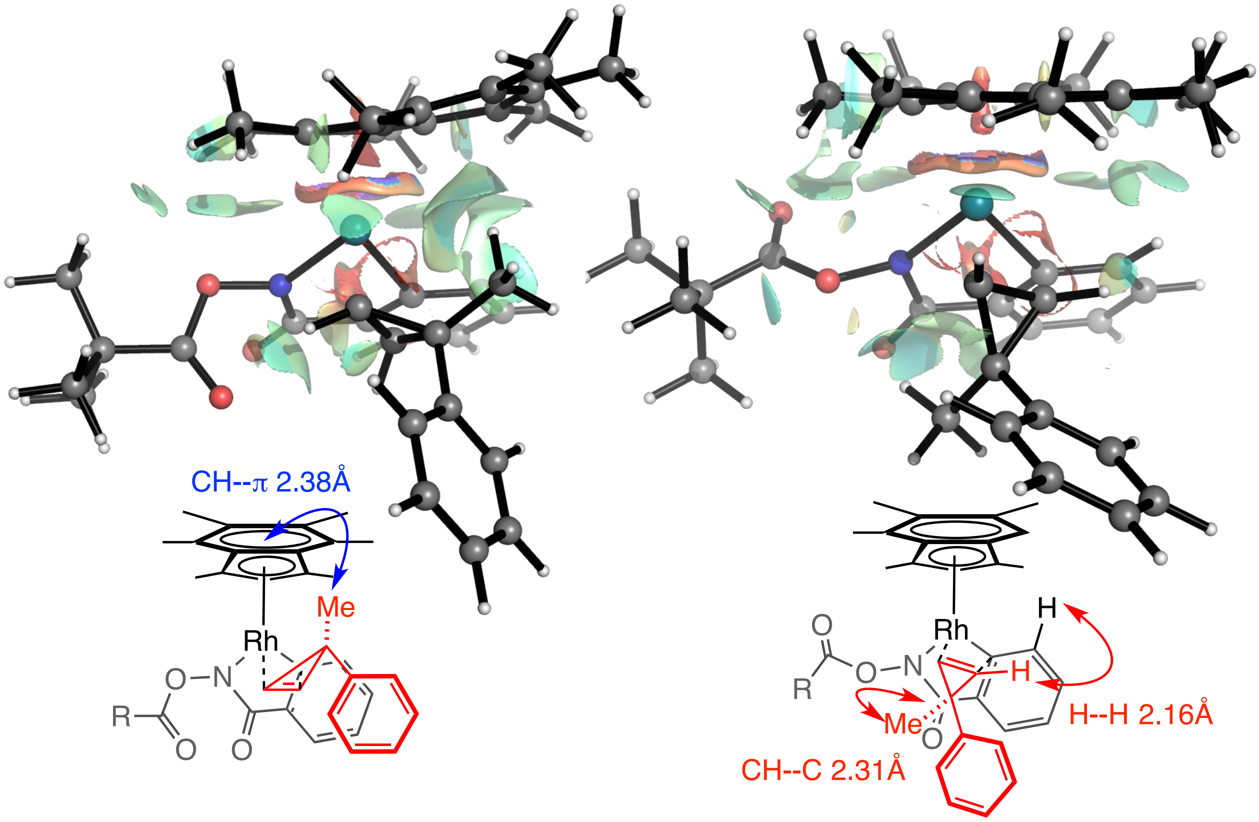

Figure S6. Non-Covalent Interaction (NCI) isosurface for PBE/6-31G*/DGDZVP//TPSS/def2-TZVP TSs for cyclopropene addition with I* ligands showing *exo-trans* (LHS) and *endo-trans* (RHS) structures.

6. Product structures

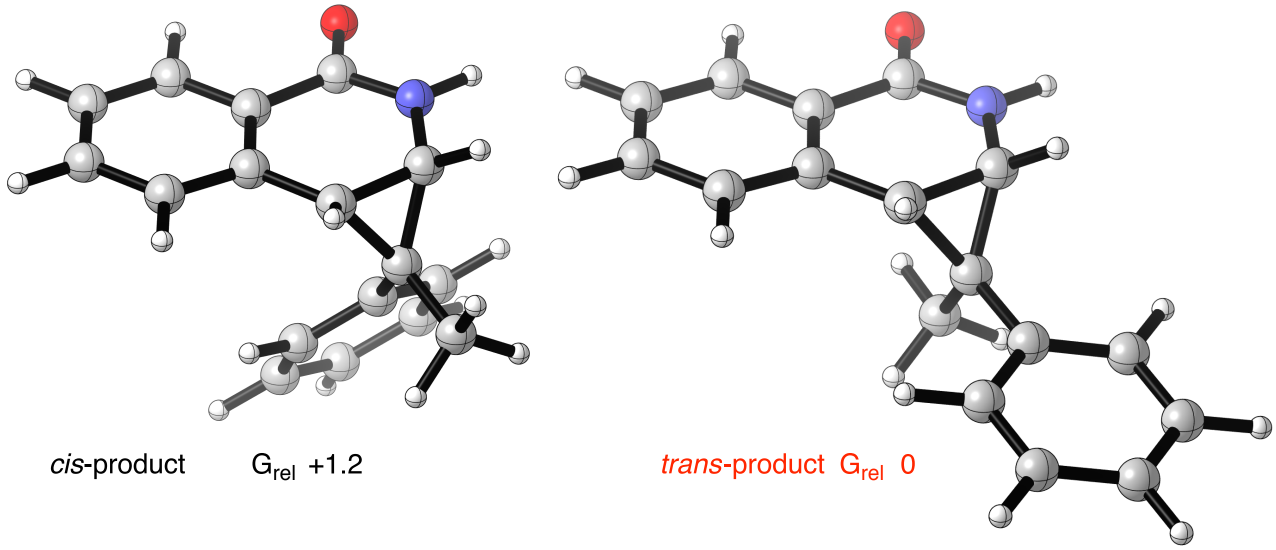

Figure S7. Comparison of TPSS/def2-TZVP diastereomeric product structures addition (relative Gibbs energies in kcal/mol.

7. Dispersion effects

With both Cp* and I* several density functionals were examined, along with the inclusion of an interatomic density-independent dispersion correction with a Becke-Johnson damping function at short range (GD3BJ). For the Cp* ligand we optimized TS structures with each functional, giving the relative Gibbs energies in Table S1. For I*, TS optimizations were performed with PBE and TPSS functionals; dispersion-corrected values were obtained as single-point corrections on these geometries. Comparing DFT results against experiment we observe that explicitly dispersion-corrected functionals (TPSS-D3, PBE-D3) and M06 (which captures medium-range correlation as a hybrid meta-GGA functional) fail to reproduce the correct sense of diastereoselectivity. This is true for both ligand systems. In each case this was because the *cis-endo* TS, in which the phenyl group is oriented towards the Cp* ligand, became the most stable in contrast to the TPSS and PBE results. A plausible origin of this is the artificial over-stabilization of the nonbonding interation involving the phenyl group and ligand. This over-estimation of non-covalent interactions experimentally-determined in solution by dispersion-corrected DFT has been noted previously.[10] The TPSS and PBE functionals without dispersion corrections do not suffer from this unphysical over-stabilization, and both correctly account for the observed sense of diastereoselectivity.

**Ligand**	**Functional**	**ΔΔG‡ (kcal /mol)**				**d.r.**	**correct sense?**
		trans-exo	***trans-endo***	***cis-exo***	***cis-endo***		
[a]**Cp***	TPSS	**0.0**	**0.9**	**1.8**	**2.7**	**21:1**	✓
[a]**Cp***	TPSS-D3	**3.4**	**4.0**	**2.3**	**0.0**	**1:230**	✗
[a]**Cp***	PBE	**0.0**	**0.6**	**1.8**	**1.4**	**10:1**	✓
[a]**Cp***	PBE-D3	**3.0**	**3.2**	**2.1**	**0.0**	**1:100**	✗
[a]**Cp***	M06	**1.5**	**2.8**	**0.4**	**0.0**	**1:18**	✗
[a]**I***	TPSS	**0.0**	**1.3**	**3.0**	**2.5**	**168:1**	✓
[b]**I***	TPSS-D3	**0.2**	**2.8**	**1.7**	**0.0**	**1:2**	✗
[a]**I***	PBE	**0.0**	**0.2**	**1.1**	**0.8**	**4.5:1**	✓
[b]**I***	PBE-D3	**1.1**	**2.1**	**0.2**	**0.0**	**1:9**	✗

Table S1. Comparison of TS relative Gibbs energies obtained from optimizations with different density functionals. The basis set used in each case is def2-TZVP; [a] Optimization with the functional shown; [b] Single point calculation using TPSS optimized structures.

[10] Yang, L.; Adam, C.; Nichol, G. S.; Cockroft, S. L. *Nat. Chem.* **2013**, *5*, 1006-101.

8. Cartesian Coordinates

Geometries and absolute values (Hartree) for SCF energy, zero-point vibrational energy (ZPE), enthalpy, RRHO Gibbs energy and quasi-harmonic Gibbs energy (at 298.15 K) for all intermediates and transition structures. All *Gaussian* output files have been deposited and are available for download from **http://dx.doi.org/10.5281/zenodo.53736**

Cp* Intermediate A:

E: -1246.83862
ZPE: 0.444534
H: -1246.362439
G: -1246.45298
qh-G: -1246.447477

0 1
Rh 0.827974 -0.352512 -0.126413
N -0.670246 0.871004 -0.43171
C 2.221948 -1.729783 0.817333
C 2.294336 -1.87753 -0.599239
C 0.975386 -2.3198 -1.067006
C 0.112657 -2.472007 0.055693
C 0.851151 -2.024018 1.215412
O -2.03199 0.506332 -0.712013
C -0.527506 2.262245 -0.338937
C 0.91519 2.580403 -0.134686
C 1.786471 1.483112 -0.020173
C 1.357415 3.900229 -0.074301
C 3.147075 1.76181 0.144659
C 2.72035 4.154387 0.096762
H 0.632747 4.706107 -0.162985
C 3.609871 3.084775 0.200497
H 3.874259 0.957875 0.231236
H 3.085421 5.1768 0.145959
H 4.673545 3.276099 0.32758
O -1.445438 3.067388 -0.416512
C -2.821956 0.303271 0.397571
O -2.397374 0.238738 1.528849
C -4.30025 0.218885 -0.006339
C -5.04605 -0.641317 1.028562
H -6.119726 -0.623683 0.810815
H -4.884323 -0.261069 2.04014
H -4.709273 -1.683791 0.996419
C -4.500142 -0.353655 -1.422114
H -4.09957 -1.370325 -1.503008
H -4.017795 0.268738 -2.179204
H -5.57344 -0.396489 -1.640534
C -4.831677 1.674928 0.04246
H -4.275132 2.31762 -0.644968
H -4.738103 2.087595 1.051604
H -5.890967 1.678509 -0.239764
C 0.320735 -1.965538 2.613204
H 0.320163 -2.97217 3.055369
H -0.702583 -1.581507 2.62946
H 0.938152 -1.320703 3.242945
C -1.289676 -3.001332 0.053075
H -1.880974 -2.559133 0.858493
H -1.288817 -4.090393 0.197672
H -1.794901 -2.793603 -0.894184
C 0.63345 -2.614594 -2.494111
H -0.441206 -2.529529 -2.672847
H 0.940025 -3.638152 -2.751867
H 1.150218 -1.932898 -3.175149
C 3.507051 -1.734708 -1.467459
H 3.257157 -1.277751 -2.429137
H 3.952635 -2.718769 -1.66863
H 4.26902 -1.113392 -0.991299
C 3.339601 -1.411193 1.764225
H 4.221956 -1.046157 1.233945
H 3.634249 -2.313859 2.315827
H 3.045984 -0.651053 2.493545

Cp* *exo-trans* TS:

E: -1634.034324
ZPE: 0.609739
H: -1633.383899
G: -1633.493409
qh-G: -1633.485892

0 1
Rh -0.239922 -0.964479 -0.414788
N -1.372909 0.382044 0.663483
C -1.122861 -3.0728 -0.540008
C 0.073383 -3.027924 -1.355594
C -0.12301 -2.043719 -2.382966
C -1.4198 -1.447721 -2.193319
C -2.038944 -2.113856 -1.054659

O -2.294942 1.312539 0.084745
C -1.423586 0.339449 2.02854
C -0.414009 -0.652254 2.510452
C 0.524806 -1.16022 1.58645
C -0.402045 -1.06911 3.840364
C 1.469495 -2.098594 2.027884
C 0.535734 -2.009775 4.269176
H -1.141621 -0.645531 4.515518
C 1.463625 -2.52481 3.358675
H 2.225396 -2.479767 1.348286
H 0.547756 -2.338302 5.304766
H 2.202153 -3.252446 3.687826
O -2.160379 1.006701 2.761205
C 1.095897 0.605542 -0.810864
C 1.377213 0.313066 0.551602
C -1.917173 2.64045 0.207839
O -0.827615 2.999328 0.581745
C -3.087923 3.563995 -0.152966
C -3.991916 3.643464 1.102468
H -4.834711 4.313975 0.895595
H -3.434694 4.033472 1.959018
H -4.375907 2.656134 1.371525
C -3.906159 3.031885 -1.346
H -4.369352 2.068749 -1.116976
H -3.281321 2.916521 -2.238932
H -4.701766 3.748116 -1.58159
C -2.523891 4.95707 -0.480601
H -1.887343 4.927492 -1.371623
H -1.925056 5.340507 0.348884
H -3.351879 5.649318 -0.670743
C -3.429406 -1.852275 -0.560769
H -4.145867 -2.446112 -1.144832
H -3.696947 -0.798786 -0.665814
H -3.542951 -2.126097 0.490647
C -2.100106 -0.473908 -3.10941
H -2.741664 0.211587 -2.550597
H -2.727717 -1.00384 -3.839881
H -1.372487 0.122842 -3.66601
C 0.793973 -1.776341 -3.539885
H 0.747279 -0.731112 -3.854903
H 0.500854 -2.398558 -4.396607
H 1.83237 -2.010704 -3.296996
C 1.174631 -4.04589 -1.288662
H 2.051985 -3.744247 -1.865383
H 0.817595 -4.997005 -1.708027
H 1.487793 -4.243557 -0.259481
C -1.353775 -4.020062 0.598135
H -0.460331 -4.115049 1.22257
H -1.608396 -5.019618 0.220279
H -2.174304 -3.684651 1.236729
H 0.751746 1.554492 -1.205845
C 2.517646 0.224018 -0.433322
H 1.283668 0.993578 1.386684
C 3.184727 -1.07673 -0.841274
H 3.976263 -1.347091 -0.1326
H 2.449734 -1.881848 -0.87886
H 3.647274 -0.970608 -1.829565
C 3.502805 1.371789 -0.341797
C 3.758958 2.180178 -1.456519
C 4.206576 1.6195 0.843843
C 4.700197 3.207934 -1.390841
H 3.211996 2.004646 -2.380087
C 5.145905 2.648868 0.912739
H 4.012267 1.00499 1.719937
C 5.39717 3.445276 -0.205334
H 4.88492 3.827502 -2.264749
H 5.678835 2.831265 1.842365
H 6.125978 4.249472 -0.151254

Cp* *exo-cis* TS:

E: -1634.031315
ZPE: 0.609774
H: -1633.380824
G: -1633.490186
qh-G: -1633.4830

0 1
Rh 0.185443 0.059711 0.579903
N -1.707418 0.542868 -0.112895
C 1.033904 1.206765 2.373528
C 1.891281 0.075909 2.069756
C 1.11182 -1.121542 2.242913
C -0.234202 -0.740915 2.564872
C -0.266147 0.716015 2.662128
O -2.898617 -0.204246 0.17133
C -1.945187 1.715048 -0.771851
C -0.635488 2.396888 -1.012997
C 0.558443 1.672728 -0.795742
C -0.594457 3.726563 -1.427349
C 1.786245 2.324194 -0.978496
C 0.633193 4.36435 -1.61628
H -1.539461 4.240259 -1.586561
C 1.818314 3.662168 -1.379507
H 2.715836 1.787415 -0.822832

H 0.667554 5.400629 -1.941054
H 2.779007 4.152883 -1.519511
O -3.043038 2.158495 -1.123461
C 0.232706 -1.328706 -0.993051
C 0.474804 -0.088845 -1.669604
C -3.450252 -0.840144 -0.924114
O -2.901709 -0.942037 -1.995845
C -4.861171 -1.356274 -0.609815
C -5.066321 -2.683177 -1.364606
H -6.102853 -3.017135 -1.242616
H -4.406573 -3.466656 -0.974256
H -4.85678 -2.558551 -2.429688
C -5.832099 -0.283611 -1.165653
H -5.686139 -0.153862 -2.241813
H -5.667798 0.682321 -0.680009
H -6.864529 -0.605688 -0.984992
C -5.118428 -1.556228 0.893978
H -5.028656 -0.616731 1.44514
H -4.421607 -2.282076 1.32703
H -6.135616 -1.940431 1.034216
C -1.466654 1.524624 3.050398
H -1.552699 1.569737 4.144733
H -2.384395 1.08235 2.656017
H -1.399607 2.549036 2.675986
C -1.352217 -1.665112 2.945144
H -2.322053 -1.261767 2.645655
H -1.369452 -1.815135 4.034092
H -1.233635 -2.645184 2.475397
C 1.634275 -2.526417 2.250878
H 0.921505 -3.227539 1.808775
H 1.81195 -2.840744 3.288767
H 2.578068 -2.604265 1.709959
C 3.378964 0.146806 1.89634
H 3.765426 -0.705627 1.334384
H 3.870313 0.153891 2.880147
H 3.679061 1.059871 1.376238
C 1.471673 2.637794 2.435555
H 2.243444 2.856287 1.693448
H 1.887861 2.858202 3.428097
H 0.636874 3.321546 2.263374
H -0.61897 -1.977527 -1.166929
C 1.451351 -1.222378 -1.883826
H -0.150104 0.318711 -2.453594
C 1.264258 -1.878181 -3.262337
C 2.890018 -1.31422 -1.455142
C 3.345356 -2.470908 -0.807517
C 3.838558 -0.363913 -1.856865
C 4.700415 -2.654453 -0.528711
H 2.62451 -3.237619 -0.535853
C 5.194056 -0.537789 -1.571389
H 3.51155 0.50878 -2.415058
C 5.630095 -1.681253 -0.900687
H 5.032102 -3.560157 -0.026663
H 5.91216 0.215874 -1.884876
H 6.685656 -1.820208 -0.683283
H 1.539874 -2.938171 -3.214192
H 0.222556 -1.803854 -3.589496
H 1.906875 -1.398344 -4.009462

Cp* *endo-trans* TS:

E: -1634.032896
ZPE: 0.609701
H: -1633.382487
G: -1633.491588
qh-G: -1633.48445

0 1
Rh -0.02105 -0.941927 -0.457227
N -1.216529 0.329313 0.665271
C -0.364028 -3.174922 -0.676345
C 0.549107 -2.697787 -1.698481
C -0.16938 -1.759747 -2.523733
C -1.479335 -1.586358 -1.966274
C -1.599554 -2.4825 -0.820279
O -1.901266 1.468457 0.100819
C -1.039273 0.465804 2.013141
C -0.000024 -0.50131 2.465781
C 0.865849 -1.084659 1.517332
C 0.081717 -0.842329 3.819127
C 1.781512 -2.051411 1.97378
C 1.001309 -1.791212 4.254928
H -0.60627 -0.351247 4.502977
C 1.842399 -2.404391 3.319646
H 2.484961 -2.507817 1.28037
H 1.0639 -2.056904 5.306425
H 2.566603 -3.149283 3.641729
O -1.626512 1.261285 2.752537
C -3.268214 1.457616 0.225609
O -3.921548 0.495739 0.56324
C -3.835774 2.842801 -0.122922
C -5.355657 2.712738 -0.313023
H -5.781179 3.701436 -0.519295
H -5.829015 2.30498 0.583599
H -5.596333 2.050914 -1.151731

C -3.187637 3.40775 -1.404105
H -3.374302 2.755429 -2.264879
H -2.107833 3.526448 -1.286576
H -3.624382 4.388923 -1.623714
C -3.534642 3.783709 1.068782
H -2.458083 3.89283 1.220389
H -3.968175 3.394827 1.99459
H -3.968626 4.769739 0.863537
C -2.8409 -2.684788 -0.00617
H -3.571406 -3.276452 -0.574887
H -3.302714 -1.728717 0.257101
H -2.621228 -3.224553 0.918604
C -2.600528 -0.786757 -2.557619
H -3.320821 -0.497078 -1.790223
H -3.132598 -1.388191 -3.308043
H -2.234345 0.117386 -3.050949
C 0.345518 -1.128483 -3.781555
H -0.156732 -0.180372 -3.98889
H 0.16733 -1.797037 -4.634683
H 1.423221 -0.944428 -3.733035
C 1.92547 -3.227735 -1.975312
H 2.587658 -2.447423 -2.362542
H 1.88815 -4.030901 -2.723985
H 2.382679 -3.641814 -1.072867
C -0.086884 -4.269073 0.308552
H 0.983341 -4.394586 0.486551
H -0.47438 -5.220559 -0.080106
H -0.567231 -4.07772 1.270952
C 1.899228 -0.089923 0.142193
C 1.314658 0.595355 -0.989902
H 2.787784 -0.707777 0.141871
C 1.915543 1.429285 0.109005
H 1.667022 0.57072 -2.016442
C 1.195604 2.398576 1.040418
H 1.385546 3.417269 0.682681
H 0.121069 2.249306 1.065732
H 1.593093 2.328748 2.058823
C 3.307473 1.941721 -0.231606
C 4.398662 1.64745 0.5963
C 3.517531 2.750684 -1.35572
C 5.669757 2.145577 0.305576
H 4.249377 1.027044 1.477082
C 4.786931 3.251286 -1.647313
H 2.678207 2.991219 -2.004367
C 5.868433 2.948226 -0.818232
H 6.504921 1.907418 0.959494
H 4.931458 3.878642 -2.523339
H 6.857659 3.336119 -1.045886

Cp* *endo-cis* TS:

E: -1634.031554
ZPE: 0.609956
H: -1633.381401
G: -1633.487318
qh-G: -1633.481587

0 1
Rh -1.267731 -0.31529 0.310879
N 0.658589 -0.256509 -0.437629
C -3.134162 -1.127435 -0.699332
C -3.345421 -0.995388 0.729028
C -2.424894 -1.885491 1.391016
C -1.594766 -2.491057 0.39163
C -2.049298 -2.023825 -0.913646
O 1.744132 -1.037977 0.08899
C 1.071945 0.706918 -1.309114
C -0.088059 1.599563 -1.60807
C -1.243316 1.543503 -0.802742
C -0.041722 2.430926 -2.730367
C -2.35373 2.318276 -1.181568
C -1.143172 3.204956 -3.08412
H 0.87571 2.432699 -3.313892
C -2.306371 3.134438 -2.309433
H -3.255387 2.309934 -0.572819
H -1.104555 3.851301 -3.95663
H -3.177632 3.729059 -2.574599
O 2.195042 0.822993 -1.804185
C 2.264986 -1.968612 -0.774911
O 1.748295 -2.308056 -1.816785
C 3.607549 -2.50807 -0.258734
C 3.733277 -3.98285 -0.6838
H 4.731438 -4.353088 -0.423932
H 3.584593 -4.089239 -1.760974
H 2.993794 -4.608778 -0.170779
C 3.765804 -2.382417 1.26694
H 2.975168 -2.923368 1.798667
H 3.742694 -1.338837 1.587088
H 4.729046 -2.815168 1.561953
C 4.699387 -1.668628 -0.969586
H 4.586984 -0.605604 -0.741019
H 4.632134 -1.792031 -2.05441
H 5.687195 -2.008722 -0.635817
C -1.535699 -2.497629 -2.239204
H -1.901689 -3.513775 -2.441804

H -0.442604 -2.513483 -2.261235
H -1.884184 -1.850147 -3.047976
C -0.578542 -3.569336 0.618189
H 0.132555 -3.613898 -0.208159
H -1.079606 -4.545266 0.684114
H -0.023295 -3.414395 1.547169
C -2.393523 -2.178857 2.860411
H -1.406589 -2.521713 3.180531
H -3.117452 -2.969921 3.098876
H -2.664162 -1.30212 3.457055
C -4.448733 -0.228446 1.397461
H -4.139178 0.147557 2.377204
H -5.327611 -0.869 1.553074
H -4.768392 0.625683 0.794088
C -3.966107 -0.498884 -1.775759
H -4.544336 0.347917 -1.399494
H -4.675976 -1.238218 -2.170311
H -3.352148 -0.142997 -2.606726
C -1.248097 1.681592 1.18824
C -0.659053 0.683139 2.055797
H -2.18123 2.216721 1.316983
C 0.038917 2.00433 1.917068
H -1.063945 0.296266 2.986666
C -0.212411 2.932697 3.118746
C 1.395643 2.290569 1.332463
C 1.592485 3.410443 0.513831
C 2.512637 1.567368 1.764397
C 2.875504 3.782383 0.115172
H 0.733634 3.988353 0.180801
C 3.796995 1.945208 1.375623
H 2.365585 0.701787 2.404874
C 3.982544 3.053258 0.548508
H 3.009849 4.641058 -0.537437
H 4.655614 1.372845 1.718123
H 4.982834 3.341629 0.236954
H -1.206071 2.766977 3.550811
H 0.541328 2.749884 3.892808
H -0.129645 3.982199 2.815063

I* Intermediate A:

E: -1479.210159
ZPE: 0.54581
H: -1478.626928
G: -1478.733028
qh-G: -1478.725784

0 1

Rh -0.133296 0.007360 -0.245781
N 1.587344 -0.919348 -0.140887
C -0.888204 1.107548 -1.874660
C 0.145595 1.955084 -1.281578
C -0.210021 2.218210 0.060441
C -1.563642 1.666194 0.291084
O 2.864891 -0.335873 -0.437729
C 1.693448 -2.300671 0.043673
C 0.320138 -2.861008 0.184176
C -0.736945 -1.943497 0.072746
C 0.111741 -4.217439 0.425585
C -2.037509 -2.433278 0.220272
C -1.195489 -4.690144 0.561799
H 0.970914 -4.879231 0.505436
C -2.262937 -3.796634 0.462935
H -2.890930 -1.762660 0.157319
H -1.380339 -5.744820 0.748199
H -3.282853 -4.159769 0.575424
O 2.746441 -2.923512 0.102766
C 3.491085 0.249494 0.642927
O 2.955917 0.456622 1.706559
C 4.963659 0.536617 0.317937
C 5.414132 1.768912 1.121754
H 6.492610 1.914236 0.994526
H 5.196923 1.639971 2.184857
H 4.905850 2.676507 0.775763
C 5.221597 0.751512 -1.184492
H 4.658595 1.608022 -1.570982
H 4.950722 -0.131573 -1.767620
H 6.288325 0.953503 -1.336653
C 5.740586 -0.717058 0.798647
H 5.390996 -1.614490 0.280288
H 5.607989 -0.864917 1.874773
H 6.808403 -0.577412 0.593145
C -2.415467 1.687692 1.435286
C 0.634731 2.990071 1.030822
H 0.718671 2.479387 1.991942
H 0.211853 3.988281 1.205074
H 1.646477 3.118926 0.641770
C 1.377000 2.414537 -2.002477
H 2.132946 2.784270 -1.307007
H 1.130631 3.229436 -2.696244
H 1.827163 1.604410 -2.582734
C -0.826214 0.623146 -3.293100
H 0.214010 0.517009 -3.611194
H -1.309088 1.343895 -3.968604
H -1.317510 -0.342275 -3.415970

C -4.160424 0.599998 0.053529
C -3.340094 0.549348 -1.069549
C -2.009836 1.055160 -0.938466
C -3.679851 1.116717 1.309374
C -4.606500 1.025082 2.504536
H -4.062761 1.077753 3.448090
H -5.355915 1.828971 2.503808
H -5.154942 0.078880 2.499016
C -5.592718 0.109242 0.015666
H -5.694722 -0.857955 0.526210
H -6.262193 0.810021 0.524763
H -5.964228 -0.022075 -1.000143
C -1.951961 2.332651 2.721792
H -2.786142 2.556734 3.386892
H -1.251742 1.685356 3.265157
H -1.437624 3.275546 2.523915
C -3.792611 -0.002458 -2.399981
H -3.373303 -1.003010 -2.567867
H -4.876787 -0.090196 -2.467477
H -3.462818 0.635442 -3.224357

I* *endo-cis* TS:

E: -1866.402716
ZPE: 0.710477
H: -1865.645929
G: -1865.768507
qh-G: -1865.760847

0 1
Rh -0.700452 -0.057052 -0.371758
N 1.337504 -0.153834 -0.538465
C -1.235881 -2.118081 -0.9002
C -1.22327 -1.350988 -2.137629
C -2.221119 -0.340374 -2.066521
C -2.92698 -0.494361 -0.790359
O 2.135289 -1.182944 0.066549
C 2.07043 0.92127 -0.955447
C 1.137987 2.033924 -1.302282
C -0.205212 1.977942 -0.877693
C 1.595155 3.095097 -2.088064
C -1.070532 3.009591 -1.281037
C 0.728442 4.113809 -2.474492
H 2.639611 3.083079 -2.389653
C -0.610435 4.060862 -2.073125
H -2.105742 3.014645 -0.946808
H 1.085671 4.939073 -3.084183
H -1.300106 4.850438 -2.363278
O 3.296855 0.969044 -1.061826
C 2.847784 -1.961457 -0.81497
O 2.686898 -1.967094 -2.014719
C 3.898833 -2.802462 -0.074531
C 4.114386 -4.108683 -0.860631
H 4.934214 -4.676057 -0.40583
H 4.364541 -3.897243 -1.902848
H 3.215472 -4.735626 -0.844604
C 3.502039 -3.121055 1.378366
H 2.559148 -3.677528 1.423103
H 3.392975 -2.212555 1.974262
H 4.282566 -3.742303 1.833286
C 5.202791 -1.96548 -0.090456
H 5.059439 -1.007101 0.41501
H 5.518014 -1.763491 -1.118147
H 5.995531 -2.527464 0.417844
C -4.047876 0.190835 -0.219152
C -2.564898 0.574335 -3.20784
H -2.43608 1.631906 -2.959218
H -3.604057 0.418836 -3.520616
H -1.923686 0.36557 -4.066498
C -0.333933 -1.651149 -3.306528
H -0.364923 -0.852333 -4.050416
H -0.657859 -2.580385 -3.794559
H 0.706801 -1.771624 -2.992248
C -0.391288 -3.346687 -0.70489
H 0.46657 -3.321487 -1.379406
H -0.971269 -4.24964 -0.941244
H -0.017432 -3.440528 0.315426
C -3.937413 -1.373543 1.695139
C -2.876328 -2.084868 1.149531
C -2.351538 -1.641674 -0.104691
C -4.54001 -0.266816 0.995711
C -5.759786 0.384 1.615076
H -6.311214 0.987921 0.893379
H -6.450042 -0.368954 2.008865
H -5.489483 1.038652 2.455457
C -4.517376 -1.745854 3.045272
H -4.785576 -0.850785 3.614111
H -5.432569 -2.345393 2.942721
H -3.813644 -2.316578 3.651932
C -4.697213 1.366156 -0.91867
H -5.658458 1.083439 -1.368639
H -4.89747 2.18352 -0.218572
H -4.06847 1.76094 -1.713108
C -2.287307 -3.30373 1.821543
H -1.324213 -3.076205 2.297418

H -2.951161 -3.708535 2.586106
H -2.108658 -4.098931 1.094029
C -0.804412 1.597676 1.016753
C -0.549392 0.328694 1.673016
H -1.704725 2.200214 1.04026
C 0.229118 1.530988 2.118392
H -1.230598 -0.249346 2.29314
C 1.708596 1.786509 2.013841
C 2.18202 3.040871 1.607457
C 2.621838 0.849246 2.5082
C 3.541506 3.342507 1.673274
H 1.480155 3.780075 1.228858
C 3.980218 1.153303 2.584185
H 2.258443 -0.122893 2.830307
C 4.444531 2.400743 2.166114
H 3.895901 4.312877 1.335446
H 4.678755 0.414725 2.969724
H 5.504673 2.634116 2.215839
C -0.308134 2.117684 3.436675
H 0.184199 1.632831 4.287022
H -0.091547 3.190328 3.492604
H -1.390266 1.969256 3.526015

I* *endo-trans* TS:

E: -1866.404239
ZPE: 0.710784
H: -1865.647011
G: -1865.771219
qh-G: -1865.762626

0 1
Rh -0.052034 -0.694587 0.26559
N -1.89462 0.170781 0.495574
C -0.577188 -2.338632 -1.078052
C -0.802345 -2.805207 0.283355
C 0.435728 -2.814852 0.983096
C 1.484979 -2.404587 0.044218
O -2.668534 0.654132 -0.621681
C -2.223289 0.818444 1.658057
C -1.133275 0.633135 2.654921
C 0.140314 0.218513 2.211959
C -1.388272 0.849057 4.012461
C 1.131958 -0.005068 3.184213
C -0.392535 0.633289 4.960463
H -2.387335 1.172946 4.293485
C 0.866474 0.192952 4.538371
H 2.134205 -0.300687 2.882135
H -0.590262 0.800206 6.015522
H 1.656442 0.019975 5.265963
O -3.256014 1.464519 1.854387
C -3.924935 0.111087 -0.748675
O -4.320011 -0.859533 -0.143986
C -4.7475 0.928935 -1.756335
C -5.968297 0.095027 -2.179015
H -6.593718 0.686244 -2.857439
H -6.564923 -0.193795 -1.310209
H -5.664387 -0.819937 -2.698707
C -3.912524 1.311228 -2.995556
H -3.552589 0.42 -3.522299
H -3.050574 1.925566 -2.724905
H -4.541828 1.880954 -3.689149
C -5.216878 2.212623 -1.02893
H -4.364621 2.820303 -0.715211
H -5.800892 1.965586 -0.137693
H -5.844687 2.799989 -1.709736
C 2.907077 -2.295939 0.175096
C 0.593468 -3.330301 2.385306
H 0.992628 -2.577414 3.071656
H 1.262069 -4.19912 2.399999
H -0.373037 -3.648827 2.780856
C -2.133854 -3.2426 0.814888
H -2.128611 -3.30925 1.904996
H -2.391073 -4.232898 0.415161
H -2.922745 -2.540204 0.531763
C -1.663903 -2.33442 -2.11724
H -2.64155 -2.295203 -1.632991
H -1.625753 -3.256639 -2.713643
H -1.589819 -1.48613 -2.798799
C 3.007983 -1.673334 -2.217917
C 1.636993 -1.819789 -2.391079
C 0.854941 -2.176294 -1.248273
C 3.63883 -1.942858 -0.950771
C 5.148201 -1.848117 -0.876573
H 5.544704 -2.344315 0.009938
H 5.616817 -2.310429 -1.751231
H 5.485561 -0.802656 -0.849044
C 3.902408 -1.237276 -3.360549
H 4.650143 -0.517391 -3.014077
H 4.451186 -2.085485 -3.792499
H 3.341586 -0.760559 -4.164793
C 3.609737 -2.573379 1.48762
H 4.187503 -3.506017 1.440008
H 4.313807 -1.772401 1.73701
H 2.909746 -2.664784 2.314116

C 0.962835 -1.637869 -3.731828
H 0.416839 -0.686622 -3.783696
H 1.678675 -1.658555 -4.554049
H 0.238646 -2.436065 -3.91301
C 1.168544 1.04373 0.69227
C 0.755944 0.984168 -0.695374
H 2.166785 0.908333 1.089346
C 0.678867 2.309487 0.011185
H 1.373941 0.749407 -1.558034
C -0.574231 3.117788 0.329427
H -0.665555 3.916483 -0.416021
H -1.483265 2.525695 0.295744
H -0.490614 3.593034 1.313001
C 1.844694 3.230017 -0.319827
C 2.64567 3.7584 0.701023
C 2.113242 3.601969 -1.64319
C 3.692513 4.633365 0.406448
H 2.444105 3.484231 1.734044
C 3.158293 4.477896 -1.940082
H 1.495707 3.204107 -2.44515
C 3.953327 4.994961 -0.915912
H 4.30336 5.033956 1.211583
H 3.351425 4.757322 -2.972854
H 4.768337 5.675755 -1.146597

I* *exo-cis* TS:

E: -1866.404708
ZPE: 0.710875
H: -1865.647314
G: -1865.76863
qh-G: -1865.7600

0 1
Rh -0.194656 -0.147534 -0.384703
N 1.7026 -0.194597 0.479794
C -1.901251 0.707057 -1.617154
C -1.037171 -0.105493 -2.43104
C 0.287886 0.447644 -2.416216
C 0.209962 1.735194 -1.703592
O 2.898619 -0.702432 -0.134418
C 1.919734 0.348339 1.712592
C 0.605639 0.785366 2.280953
C -0.585784 0.361102 1.657244
C 0.560432 1.603546 3.409588
C -1.815135 0.775038 2.182183
C -0.667755 2.013145 3.929071
H 1.504368 1.902696 3.858626
C -1.850609 1.603607 3.307083
H -2.742113 0.453398 1.720932
H -0.705906 2.645234 4.812149
H -2.812474 1.918394 3.70637
O 3.004168 0.469933 2.292211
C -0.261523 -2.189579 -0.027357
C -0.504822 -1.633334 1.267832
C 3.388559 -1.864475 0.427477
O 2.787124 -2.534946 1.233448
C 4.807656 -2.160111 -0.078421
C 5.010081 -3.686179 -0.087339
H 6.048989 -3.914921 -0.350333
H 4.356612 -4.168223 -0.82343
H 4.790032 -4.111792 0.89447
C 5.768306 -1.511051 0.949979
H 5.614622 -1.938738 1.944893
H 5.60197 -0.43234 1.014753
H 6.803841 -1.697856 0.641347
C 5.082107 -1.582453 -1.478621
H 5.011497 -0.491929 -1.484718
H 4.38117 -1.980625 -2.220581
H 6.09614 -1.862049 -1.788059
C 1.209107 2.713091 -1.429958
C 1.414599 -0.081424 -3.260367
H 2.370549 -0.070026 -2.737433
H 1.518821 0.515609 -4.17675
H 1.209631 -1.112289 -3.561362
C -1.460608 -1.277312 -3.267267
H -0.709316 -2.070728 -3.267087
H -1.602496 -0.949849 -4.306391
H -2.403168 -1.700726 -2.918866
C -3.384309 0.494812 -1.508041
H -3.645819 -0.540577 -1.729652
H -3.916546 1.13463 -2.226272
H -3.764446 0.722186 -0.511518
H 0.583453 -2.8014 -0.326224
C -1.47603 -2.662621 0.740817
H 0.120074 -1.764333 2.141127
C -1.258736 -4.030502 1.413778
C -0.526473 4.019854 -0.264819
C -1.524382 3.08998 -0.554111
C -1.146825 1.909144 -1.25015
C 0.822099 3.850444 -0.720607
C 1.808012 4.956965 -0.406453
H 2.194332 4.866304 0.617911
H 2.664883 4.953151 -1.080442
H 1.334774 5.939606 -0.488349

C -0.847036 5.243832 0.568611
H -0.040977 5.44755 1.279615
H -0.966641 6.144728 -0.049827
H -1.762204 5.112272 1.146832
C 2.630971 2.469782 -1.875221
H 3.296489 3.287286 -1.601331
H 3.020569 1.562936 -1.401366
H 2.696755 2.334937 -2.960439
C -2.964749 3.349797 -0.172916
H -3.203857 2.933868 0.81466
H -3.178078 4.420513 -0.142211
H -3.650742 2.905459 -0.894416
C -2.922845 -2.493552 0.369657
C -3.846811 -1.945172 1.268407
C -3.409126 -3.072208 -0.811334
C -5.211147 -1.927616 0.972349
H -3.492351 -1.547204 2.214789
C -4.772596 -3.065343 -1.107342
H -2.707281 -3.543217 -1.495057
C -5.679032 -2.482325 -0.219346
H -5.911056 -1.490834 1.680436
H -5.128558 -3.519262 -2.028998
H -6.741346 -2.474321 -0.447798
H -1.54046 -4.836467 0.726242
H -0.21009 -4.163013 1.69771
H -1.883947 -4.119185 2.309497

I* *exo-trans* TS:

E: -1866.405501
ZPE: 0.710445
H: 1865.648433
G: -1865.773981
qh-G: -1865.764729

0 1
Rh 0.128564 -0.671696 -0.024029
N -1.817131 -0.934988 0.486549
C 0.519648 -1.814791 -1.848521
C 0.256419 -2.753977 -0.774548
C 1.275621 -2.65678 0.216342
C 2.29381 -1.744825 -0.299243
O -2.863062 -1.152218 -0.460623
C -2.221974 -0.975734 1.796531
C -1.049098 -0.681561 2.670166
C 0.11824 -0.166202 2.065196
C -1.100756 -0.913249 4.044222
C 1.238232 0.091042 2.869698
C 0.018862 -0.655451 4.836125
H -2.024273 -1.302075 4.466055
C 1.186431 -0.164438 4.242229
H 2.141465 0.50597 2.434166
H -0.014423 -0.835378 5.907064
H 2.062373 0.038547 4.854513
O -3.365874 -1.213371 2.19242
C -0.304438 1.300923 -0.567053
C -0.308943 1.348741 0.862242
C -3.750588 -0.095091 -0.595314
O -3.573403 0.999919 -0.121423
C -4.995399 -0.516934 -1.386691
C -5.340542 0.619145 -2.370336
H -6.294627 0.39919 -2.862386
H -4.572076 0.719589 -3.145692
H -5.425095 1.572636 -1.84305
C -6.125952 -0.656944 -0.336493
H -6.281086 0.290702 0.186676
H -5.877948 -1.42226 0.404617
H -7.055523 -0.939123 -0.844471
C -4.826848 -1.843692 -2.146047
H -4.631279 -2.674875 -1.463656
H -4.008897 -1.790296 -2.872602
H -5.750994 -2.059473 -2.695038
C 3.598934 -1.411224 0.174768
C 1.325995 -3.511629 1.453262
H 1.364051 -2.916129 2.370404
H 2.202569 -4.17021 1.438876
H 0.437862 -4.144531 1.513366
C -0.894292 -3.71534 -0.746944
H -1.193433 -3.963382 0.273235
H -0.60229 -4.645493 -1.253374
H -1.768982 -3.309778 -1.2568
C -0.275053 -1.783718 -3.126327
H -1.288491 -2.151294 -2.9483
H 0.18447 -2.430894 -3.886913
H -0.357837 -0.780466 -3.544264
H -1.13796 1.560193 -1.209109
C 0.656642 2.246962 0.127585
H -1.138976 1.659104 1.482286
C 2.163717 2.0567 0.144487
H 2.604466 2.543768 1.022556
H 2.42897 0.996391 0.154318
H 2.607035 2.514839 -0.746732
C 0.25688 3.709004 0.140342
C -0.170087 4.353139 -1.026736
C 0.34293 4.453986 1.324673

C -0.497859 5.709898 -1.013998
H -0.248528 3.786293 -1.951125
C 0.011158 5.808399 1.341368
H 0.66305 3.964392 2.241708
C -0.408797 6.441715 0.170203
H -0.828689 6.19362 -1.929595
H 0.075648 6.368635 2.270667
H -0.670779 7.496302 0.182466
C 3.959802 -0.161773 -1.932098
C 2.655359 -0.373122 -2.360728
C 1.82923 -1.234485 -1.573152
C 4.425727 -0.676697 -0.670629
C 5.858287 -0.384329 -0.281511
H 6.100482 -0.755558 0.713713
H 6.557065 -0.856479 -0.984037
H 6.070314 0.6915 -0.302658
C 4.943063 0.642386 -2.75488
H 5.141977 1.621536 -2.297799
H 5.906058 0.125756 -2.823506
H 4.594548 0.813695 -3.772992
C 4.088142 -1.889651 1.525938
H 4.637616 -1.101391 2.049251
H 3.261801 -2.183696 2.168719
H 4.764582 -2.749544 1.430522
C 2.077676 0.276415 -3.594997
H 1.124909 0.753285 -3.34133
H 2.73082 1.050444 -3.997898
H 1.882226 -0.448348 -4.394171
