# Supplementary material for: Heptamethylindenyl (Ind*) enables diastereoselective benzamidation of cyclopropenes via Rh(iii)-catalyzed C–H activation
Source: Chem Sci. 2016 Sep 23;8(2):1015–20. doi: 10.1039/c6sc02587k (PMC5354047; doi:10.1039/c6sc02587k)
Supplement: Supplementary file 2 [file SC-008-C6SC02587K-s002.pdf]

Electronic Supplementary Material (ESI) for Chemical Science.
This journal is © The Royal Society of Chemistry 2016

Supporting Information

Heptamethylindenyl (Ind*) Enables Diastereoselective Benzamidation of Cyclopropenes *via* Rh(III)-Catalyzed C-H Activation

Natthawat Semakul,[a] and Tomislav Rovis*[a]

[a] *Department of Chemistry, Colorado State University, Fort Collins, CO 80523, USA*

4th October 2016

Note added after first publication: This Supplementary Information file replaces that originally published on 23rd September 2016, and contains corrected ^{13}C NMR characterisation data for the reported structures.

Table of Contents

1. General methods 2

2. Preparation of starting materials and rhodium precatalysts 2

2.1 O-substituted Arylbenzhydroxamate Synthesis 2

2.2 3,3-disubstituted cyclopropene Synthesis 2

2.3 Catalyst Synthesis 3

3. General procedures for Rh(III)-catalyzed amidoarylation *O*-substituted arylhydroxamate with cyclopropene 4

4. Product characterizations 5

4.1 Amide scope (Table 2) 5

4.2 Cyclcopropene scope (Table 3) 12

5. Mechanistic studies 17

5.1 C-H activation reversibility 17

5.2 Electronic preference of the reaction 17

5.3 Kinetic isotope study 18

5.4 Epimerization study 22

5.5 The role of an acyl directing group 23

6. Derivatizations of dihydroisoquinolone products 23

7. X-ray Structure of 3m 25

8. Spectra 31

1. General methods

Flash column chromatography was performed on SiliCycle Inc.® silica gel 60 (230-400 mesh). Thin Layer chromatography was performed on SiliCycle Inc.® 0.25 mm silica gel 60-F plates. Visualization was accomplished with UV light (254 nm), $KMnO_4$, or CAM.

^{1}H-NMR and ^{13}C-NMR spectra were recorded on a Varian 400 MHz spectrometers or a Bruker Avance III 500 (500 MHz) at ambient temperature. ^{1}H-NMR data are reported as the following: chemical shift in parts per million (δ, ppm) from chloroform ($CHCl_3$) taken as 7.26 ppm, integration, multiplicity (s=singlet, d=doublet, t=triplet, q=quartet, m=multiplet, dd=doublet of doublets) and coupling constant (*J* in Hz unit). ^{13}C-NMR is reported as the following: chemical shifts are reported in ppm from $CDCl_3$ taken as 77.0 ppm. Several spectra contain a probe-shielding artifact that consistently appeared on all spectra taken at that instrument over a period of months.

Mass spectra were obtained on an Agilent Technologies 6130 Quadrupole Mass Spec (LRMS, ESI+APCI).

Infrared spectra (IR) were obtained on Bruker Tensor 27 FT-IR spectrometer.

2. Preparation of starting materials and rhodium precatalysts

2.1 O-substituted Arylbenzhydroxamate Synthesis

O-pivaloyl arylhydroxamates[1] and *O*-Boc arylhydroxamates[2] were prepared by previously reported procedure.

2.2 3,3-disubstituted cyclopropene Synthesis

3,3-disubstituted cyclopropenes were synthesized according to literature procedures[3-4].

---

[1] (a) Guimond, N,; Gorelsky, S. I.; Fagnou, K. *J. Am. Chem. Soc.* **2011**, *133*, 6449. (b) Wang, H.; Glorius, F. *Angew. Chem. Int. Ed.* **2012**, *51*, 7318 (c) Hyster, T. K.; Ruhl, K. E.; Rovis, T. *J. Am. Chem. Soc.* **2013**, *135*, 5364.

[2] (a) Guimond, N,; Gorelsky, S. I.; Fagnou, K. *J. Am. Chem. Soc.* **2011**, *133*, 6449. (b) Ye, B.; Cramer, N. *Science* **2012,** *338*, 504

[3] Rubin, M.; Gevorgyan, V. *Org. Lett.* **2004**, 796.

[4] Phan, D. H. T.; Kou, K. G. M.; Dong, V. M. *J. Am. Chem. Soc.* **2010**, *132*, 16354.

2.3 Catalyst Synthesis

$[Cp^*RhCl_2]_2$[5], $[Cp^{*t\text{-}Bu}RhCl_2]_2$[6], $[Cp^{*CF3}RhCl_2]_2$[7], $[Cp^{*bisCF3Ar}RhCl_2]_2$[8] and $[Cp^{iPr}RhCl_2]_2$[9] were synthesized by reported procedures. $[Cp^{t}RhCl_2]_2$ was purchased from Sigma-Aldrich (RNI00147).

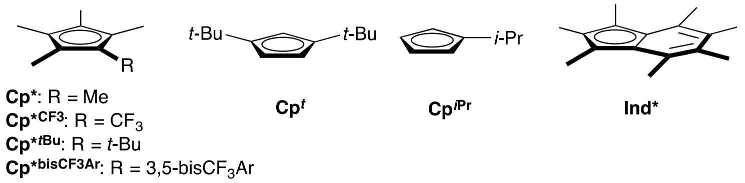

Synthesis of heptamethylindenyl rhodium chloride dimer $[Ind^*RhCl_2]_2$

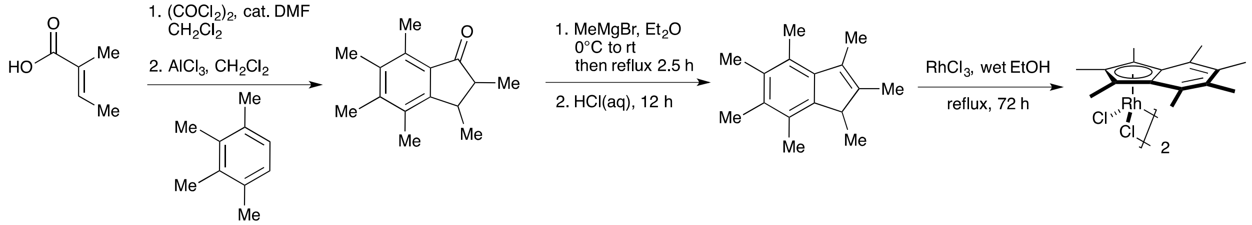

To a flame-dried round bottom flask charged with a stir bar, 2,3,4,5,6,7-hexamethylindan-1-one[10] (700 mg, 3.23 mmol, 1 equiv.), Et_2O (11 mL, 0.3 M) were added and cooled to 0°C. 3 M MeMgBr (2.16 mL, 6.47 mmol, 2 equiv.) was added to the solution dropwise. The reaction was mixture was warmed to room temperature and refluxed for 2.5 h. At 0°C, H_2O was added to the reaction mixture following by conc HCl and stirred at room temperature for 12 h. The reaction was extracted with Et_2O (×3 times), washed with satd $NaHCO_3$, H_2O, brine, dried over $MgSO_4$, filtered and solvent was evaporated under reduced pressure. The crude product was purified by column chromatography using 5% Et_2O/hexane as an eluent to afford Heptamethylindene (Ind*H)[11] (334 mg, 48% yield). The characterizations were agreed with the previously report. **^{1}H-NMR** ($CDCl_3$, 400 MHz) δ 3.28 (m, 1H), 2.61 (s, 3H), 2.42 (s, 3H), 2.35 (s, 9H), 2.05 (s, 3H), 1.33 (d, J = 4 Hz, 3H). **^{13}C-NMR** ($CDCl_3$, 100 MHz) δ 144.9, 142.9, 140.9, 133.8, 132.0, 130.8, 128.4, 126.5, 46.4, 16.7, 16.4, 16.2, 16.1, 16.0, 15.3, 12.3.

[5] Fujita, K.; Takahashi, Y.; Owaki, M.; Yamamoto, K.; Yamaguchi, R. *Org. Lett.* **2004**, *6*, 2785.
[6] Piou, T.; Rovis, T. *Nature* **2015,** *527,* 86
[7] Gassman, P. G.; Mickelson, J. W.; Sowa, J. R. *J. Am. Chem. Soc.* **1992**, *114,* 6942
[8] Davis, T. A.; Wang, C. Q.; Rovis, T., *Synlett* **2015,** *26,* 1520
[9] Piou, T.; Rovis, T. *J. Am. Chem. Soc.* **2014,** *136,* 11292
[10] Arnold, T. A.-Q.; Buffet, J.-C.; Turner, Z. R.; O'Hare, D. *J. Organomet. Chem.* **2015,** *792,* 5565
[11] O'Hare, D.; Green, J. C.; Marder, T.; Collins, S.; Stringer, G.; Kakkar, A. K.; Kaltsoyannis, N.; Kuhn, A.; Lewis, R.; Mehnert, C.; Scott, P.; Kurmoo, M.; Pugh, S. *Organometallics* **1992,** *11,* 48-55

Heptamethylindene (Ind*H) (1.26 equiv., 1.2 mmol, 260 mg) was dissolved in EtOH (0.063 M, 15 mL) and a few drops of water. $RhCl_3 \cdot H_2O$ (1 equiv., 0.95 mmol, 250 mg) was added to the reaction mixture which was subsequently refluxed for 72 h. The mixture was cooled to room temperature and the solvent was evaporated to dryness. The crude product was washed several times with hexane to remove excess ligand. The crude solid was redissolved in CH_2Cl_2 and filtered through Celite. The filtrate was concentrated which give $[Ind^*RhCl_2]_2$ complex as a red-brown solid (60 mg, 48% yield). The characterizations were agreed with the previously report[12]. **^{1}H-NMR** ($CDCl_3$, 400 MHz) δ 2.54 (s, 6H), 2.07 (s, 6H), 1.97 (s, 6H), 1.59 (s, 3H). **^{13}C-NMR** ($CDCl_3$, 126 MHz) δ 143.7, 130.4, 102.6, 95.2 (d), 81.0(d), 18.5, 17.8, 13.4, 10.3. **IR** (neat, cm^{-1}) 2922.6, 2852.3

3. General procedures for Rh(III)-catalyzed amidoarylation *O*-substituted arylhydroxamate with cyclopropene

Without any precaution of air and moisture, *O*-substituted arylhydroxamate (0.1 mmol, 1 eq), $[ind^*RhCl_2]_2$ (0.001 mmol, 1 mol%), CsOPiv (0.025 mmol, 0.25 equiv) and MeOH (1 mL, 0.1 M) were weighed into a dram vial charged with a stir bar. The mixture was stirred for 30 seconds and cyclopropene (0.11 mmol, 1.1 equiv) was then added. The reaction was stirred at room temperature for 16 h until the starting material was consumed (monitoring by TLC). The reaction was quenched with saturated $NaHCO_3$ and extracted 3 times with EtOAc. The organic layer was washed with brine, dried over $MgSO_4$, filtered, and solvent was evaporated to obtain crude product. The crude product was purified by column chromatography using 1/3 to 2/1 EtOAc/hexane to obtain the desired product.

Condition A: using *O*-pivaloyl arylhydroxamate

Condition B: using *O*-Boc arylhydroxamate

[12] Kakkar, A. K.; Stringer, G.; Taylor, N. J.; Marder, T. B. *Can. J. Chem.* **1995,** *73,* 981

4. Product characterizations

(1*S*,1a*R*,7b*R*)-1-methyl-1-phenyl-1,1a,2,7b-tetrahydro-3*H*-cyclopropa[*c*]isoquinolin-3-one (3c)

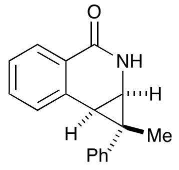

3c

General procedure B, *O*-Boc arylhydroxamate **1c'** (0.1 mmol, 23.7 mg) reacted with cyclopropene **2c** (0.11 mmol, 14.3 mg) gives the desired dihydroisoquinolone (>20:1 dr of the crude reaction mixture). The major diastereomer was obtained after purification by column chromatography using 1/3 to 2/1 EtOAc/hexane as a white solid (17.9 mg, 68% yield).

^{1}H-NMR ($CDCl_3$, 400 MHz): δ 8.20 (d, *J* = 8.0 Hz, 1H), 7.51 (t, *J* = 8.0 Hz, 1H), 7.44-7.32 (m, 6H), 7.28-7.24 (m, 1H), 6.99 (br. s, NH), 3.49 (dd, *J* = 8.0, 4.0 Hz, 1H), 2.72 (d, *J* = 8.0 Hz, 1H), 1.13 (s, 3H)

^{13}C- NMR ($CDCl_3$, 100 MHz): δ 164.1, 144.8, 136.4, 132.5, 129.5, 128.7, 128.2, 126.95, 126.86, 126.78, 126.5, 40.7, 26.7, 24.9, 13.1

IR (neat, cm^{-1}) 3174, 2923, 1658, 1599

LRMS m/z (ESI+APCI) calcd for $C_{17}H_{15}NO$ [M+H]: 250.1; Found: 250.1

NOESY

NOE interaction

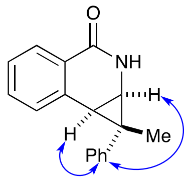

3c

4.1 Amide scope (Table 2)

(1*S*,1a*R*,7b*R*)-1,6-dimethyl-1-phenyl-1,1a,2,7b-tetrahydro-3*H*-cyclopropa[*c*]isoquinolin-3-one (3d)

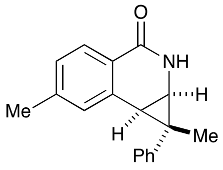

3d

General procedure A, *O*-Piv arylhydroxamate **1d** (0.1 mmol, 23.5 mg) reacted with cyclopropene **2c** (0.11 mmol, 14.3 mg) gives the desired dihydroisoquinolone (>20:1 dr of the crude reaction mixture). The major diastereomer was obtained after purification by column chromatography using 1/3 to 2/1 EtOAc/hexane as an off-white solid (21.0 mg, 90 % yield).

General procedure B, *O*-Boc arylhydroxamate **1d'** (0.1 mmol, 25.1 mg) reacted with cyclopropene **2c** (0.11 mmol, 14.3 mg) gives the desired dihydroisoquinolone (>20:1 dr of the crude reaction mixture). The major diastereomer was obtained after purification by column chromatography using 1/3 to 2/1 EtOAc/hexane as a white solid (17.9 mg, 68% yield).

^{1}H-NMR ($CDCl_3$, 400 MHz): δ 8.05 (d, *J* = 8.0 Hz, 1H), 7.39-7.32 (m, 4H), 7.28-2.22 (m, 2H), 7.17 (d, *J* = 8.0 Hz, 1H), 6.89 (br. s, 1H), 3.47 (dd, *J* = 8.0, 4.0 Hz, 1H), 2.67 (d, *J* = 8.0 Hz, 1H), 2.41 (s, 3H), 1.13 (s, 3H)

^{13}C- NMR ($CDCl_3$, 100 MHz): δ 164.3, 144.9, 143.1, 136.3, 130.0, 128.7, 128.2, 127.9, 126.8, 126.4, 124.3, 40.7, 26.7, 25.0, 21.6, 13.2

IR (neat, cm^{-1}) 3176, 3024, 2919, 1575, 1614

LRMS m/z (ESI+APCI) calcd for $C_{18}H_{17}NO$ [M+H]: 264.1; Found: 264.2

(1*S*,1a*R*,7b*R*)-6-methoxy-1-methyl-1-phenyl-1,1a,2,7b-tetrahydro-3*H*-cyclopropa[*c*]isoquinolin-3-one (3e)

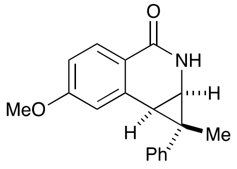

3e

General procedure A, *O*-Piv arylhydroxamate **1e** (0.1 mmol, 25.1 mg) reacted with cyclopropene **2c** (0.11 mmol, 14.3 mg) gives the desired dihydroisoquinolone (>20:1 dr of the crude reaction mixture). The major diastereomer was obtained after purification by column chromatography using 1/3 to 2/1 EtOAc/hexane as an off-white solid (24.9 mg, 89 % yield).

General procedure B, using *O*-Boc arylhydroxamate **1e'** (0.1 mmol, 26.7 mg) gives the desired dihydroisoquinolone (>20:1 dr of the crude reaction mixture). The major diastereomer was obtained after purification by column chromatography using 1/3 to 2/1 EtOAc/hexane as an off-white solid (17.9 mg, 64% yield).

¹H-NMR ($CDCl_3$, 400 MHz): δ 8.13 (d, *J* = 8.0 Hz, 1H), 7.39-7.31 (m, 4H), 7.27-7.23 (m, 1H), 6.89-6.83 (m, 2H), 6.83 (br. s, 1H), 3.88 (s, 3H), 3.47 (dd, *J* = 8.0, 4.0 Hz, 1H), 2.67 (d, *J* = 8 Hz), 1.15 (s, 3H)

¹³C- NMR ($CDCl_3$, 100 MHz): δ 164.1, 162.8, 144.9, 138.5, 130.4, 128.7, 126.7, 126.4, 119.9, 114.0, 112.9, 55.4, 41.0, 27.0, 25.2, 13.1

IR (neat, cm^{-1}) 3189, 3024, 2927, 1445, 1603, 1445, 1266

LRMS m/z (ESI+APCI) calcd for $C_{18}H_{17}NO_2$ [M+H]: 280.1; Found: 280.1

(1*S*,1a*R*,7b*R*)-1-methyl-1-phenyl-6-(trifluoromethyl)-1,1a,2,7b-tetrahydro-3*H*-cyclopropa[*c*]isoquinolin-3-one (3f)

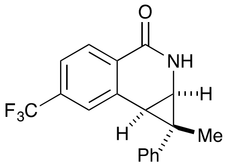

3f

General procedure A, *O*-Piv arylhydroxamate **1f** (0.1 mmol, 28.9 mg) reacted with cyclopropene **2c** (0.11 mmol, 14.3 mg) gives the desired dihydroisoquinolone (10.2:1 dr of the crude reaction mixture). The major diastereomer was obtained after purification by column chromatography using 1/3 to 2/1 EtOAc/hexane as an off-white solid (25.1 mg, 77 % yield).

General procedure B, using *O*-Boc arylhydroxamate **1f’** (0.1 mmol, 30.0 mg) gives the desired dihydroisoquinolone (17:1 dr of the crude reaction mixture). The major diastereomer was obtained after purification by column chromatography using 1/3 to 2/1 EtOAc/hexane as an off-white solid (21.2 mg, 67% yield).

¹H-NMR ($CDCl_3$, 400 MHz): δ 8.32 (d, J = 8.0 Hz, 1H), 7.70 (s, 1H), 7.62 (d, *J* = 8.0 Hz, 1H), 7.41-7.35 (m, 5H), 7.30-7.27 (m, 1H), 3.56 (dd, *J* = 8.0, 4.0 Hz, 1H), 2.76 (d, *J* = 8.0 Hz, 1H), 1.14 (s, 3H)

¹³C- NMR ($CDCl_3$, 100 MHz): δ 163.1, 144.1, 140.4, 130.1, 129.4 (q), 128.8, 128.8 (q), 127.5, 126.9, 126.8, 126.3 (q), 125.4 (q), 40.8, 26.4, 25.9, 13.2

¹⁹F-NMR ($CDCl_3$, 386 MHz): δ -63.1

IR (neat, cm^{-1}) 3200, 1560, 1311, 1168, 1127

LRMS m/z (ESI+APCI) calcd for $C_{18}H_{14}F_3NO$ [M+H]: 318.1; Found: 318.1

(1*S*,1a*R*,7b*R*)-6-chloro-1-methyl-1-phenyl-1,1a,2,7b-tetrahydro-3*H*-cyclopropa[*c*]isoquinolin-3-one (3g)

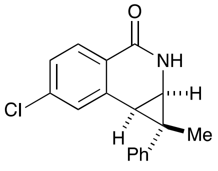

3g

General procedure A, *O*-Piv arylhydroxamate **1g** (0.1 mmol, 25.6 mg) reacted with cyclopropene **2c** (0.11 mmol, 14.3 mg) gives the desired dihydroisoquinolone (8.5:1 dr of the crude reaction mixture). The major diastereomer was obtained after purification by column chromatography using 1/3 to 2/1 EtOAc/hexane as an off-white solid (20.8 mg, 73 % yield).

General procedure B, using *O*-Boc arylhydroxamate **1g'** (0.1 mmol, 27.2 mg) gives the desired dihydroisoquinolone (>20:1 dr of the crude reaction mixture). The major diastereomer was obtained after purification by column chromatography using 1/3 to 2/1 EtOAc/hexane as an off-white solid (17.6 mg, 63% yield).

^{1}H-NMR ($CDCl_3$, 400 MHz): δ 8.11 (d, *J* = 8.0 Hz), 7.41-7.23 (m, 7H), 6.79 (br. s, 1H), 3.48 (dd, *J* = 8.0, 4.0 Hz, 1H), 2.64 (d, *J* = 8 Hz, 1H), 1.11 (s, 3H)

^{13}C- NMR ($CDCl_3$, 100 MHz): δ 163.2, 144.3, 138.7, 138.2, 129.9, 129.3, 128.8, 127.4, 126.9, 126.7, 125.4, 40.7, 26.2, 25.7, 13.3

IR (neat, cm^{-1}) 3173, 2921, 1490, 1596

LRMS m/z (ESI+APCI) calcd for $C_{17}H_{14}ClNO$ [M+H]: 284.1; Found: 284.1

(1*S*,1a*R*,7b*R*)-6-bromo-1-methyl-1-phenyl-1,1a,2,7b-tetrahydro-3*H*-cyclopropa[*c*]isoquinolin-3-one (3h)

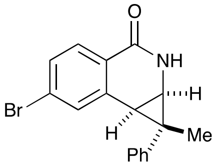

3h

General procedure A, *O*-Piv arylhydroxamate **1h** (0.1 mmol, 30.0 mg) reacted with cyclopropene **2c** (0.11 mmol, 14.3 mg) gives the desired dihydroisoquinolone (11.2:1 dr of the crude reaction mixture). The major diastereomer was obtained after purification by column chromatography using 1/3 to 2/1 EtOAc/hexane as an off-white solid (27.3 mg, 83 % yield).

^{1}H-NMR ($CDCl_3$, 400 MHz): δ 8.05 (d, *J* = 8.0 Hz, 1H), 7.60 (s, 1H), 7.50 (d, *J* = 8.0 Hz, 1H), 7.40-7.30 (m, 4H), 7.27 (t, *J* = 8.0 Hz, 1H), 6.82 (br. s, 1H), 3.50 (dd, *J* = 8.0, 4.0 Hz, 1H), 2.66 (d, *J* = 8.0 Hz, 1H), 1.14 (s, 3H)

^{13}C- NMR ($CDCl_3$, 100 MHz): δ 163.4, 144.3, 138.4, 132.2, 130.4, 130.0, 128.8, 127.3, 126.9, 126.7, 125.8, 40.7, 26.2, 25.7, 13.3

IR (neat, cm^{-1}) 2924, 1668, 1591, 1469, 1444, 1371, 765, 700

LRMS m/z (ESI+APCI) calcd for $C_{17}H_{14}BrNO$ [M+H]: 328.0; Found: 328.0, 330.0

(1*S*,1a*R*,7b*S*)-5,6,7-trimethoxy-1-methyl-1-phenyl-1,1a,2,7b-tetrahydro-3*H*-cyclopropa[*c*]isoquinolin-3-one (3i)

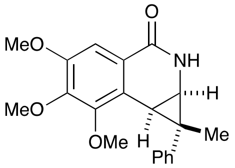

3i

General procedure A, *O*-Piv arylhydroxamate **1i** (0.1 mmol, 31.1 mg) reacted with cyclopropene **2c** (0.11 mmol, 14.3 mg) gives the desired dihydroisoquinolone (>20:1 dr of the crude reaction mixture). The major diastereomer was obtained after purification by column chromatography using 1/3 to 2/1 EtOAc/hexane as an off-white solid (20.5 mg, 62 % yield).

^{1}H-NMR ($CDCl_3$, 400 MHz): δ 7.55 (s, 1H), 7.42-7.34 (m, 4H), 7.26-7.23 (m, 1H), 6.87 (br. s, 1H), 3.97 (s, 3H), 3.93 (s, 3H), 3.91 (s, 3H), 3.39 (dd, *J* = 8.0, 4.0 Hz, 1H), 2.87 (d, *J* = 8.0 Hz, 1H), 1.13 (s, 3H)

^{13}C- NMR ($CDCl_3$, 100 MHz): δ 163.7, 152.3, 151.9, 145.9, 144.9, 128.7, 127.1, 126.5, 123.7, 122.3, 106.7, 61.1, 61.0, 56.1, 40.6, 23.9, 21.2, 13.6

IR (neat, cm^{-1}) 3200, 2937, 1575, 1597, 1576, 1479, 1113

LRMS m/z (ESI+APCI) calcd for $C_{20}H_{21}NO_4$ [M+H]: 340.2; Found: 340.2

(1*S*,1a*R*,7b*R*)-1-methyl-1-phenyl-5-(trifluoromethyl)-1,1a,2,7b-tetrahydro-3*H*-cyclopropa[*c*]isoquinolin-3-one (3j)

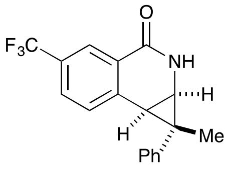

3j

General procedure A, *O*-Piv arylhydroxamate **1j** (0.1 mmol, 28.5 mg) reacted with cyclopropene **2c** (0.11 mmol, 14.3 mg) gives the desired dihydroisoquinolone (single regioselectivity, >20:1 dr of the crude reaction mixture). The major diastereomer was obtained after purification by column chromatography using 1/3 to 2/1 EtOAc/hexane as an off-white solid (28.3 mg, 85 % yield).

^{1}H-NMR ($CDCl_3$, 400 MHz): δ 8.50 (s, 1H), 7.80 (br. s, NH), 7.75 (d, *J* = 8 Hz, 1H), 7.57 (d, *J* = 8 Hz, 1H), 7.42-7.33 (m, 4H), 7.29 (d, *J* = 8 Hz, 1H), 3.58 (dd, *J* = 8.0, 4.0 Hz, 1H), 2.75 (d, *J* = 8.0 Hz), 1.14 (*s*, 3H)

^{13}C- NMR ($CDCl_3$, 100 MHz): δ 163.1, 144.1, 140.4, 130.1, 129.4 (q), 128.8, 128.8 (q), 127.5 (q), 126.9 (q), 126.8, 125.4, 123.8, 40.8, 26.4, 25.9, 13.2.

^{19}F-NMR ($CDCl_3$, 386 MHz): δ -61.8

IR (neat, cm^{-1}) 3193, 3060, 2928, 1669, 1617, 1502, 1167, 1125

LRMS m/z (ESI+APCI) calcd for $C_{18}H_{14}F_3NO$ [M+H]: 318.1; Found: 318.1

(1*S*,1a*R*,9b*R*)-1-methyl-1-phenyl-1,1a,2,5,6,7,8,9b-octahydro-3*H*-benzo[*g*]cyclopropa[*c*]isoquinolin-3-one (3k)

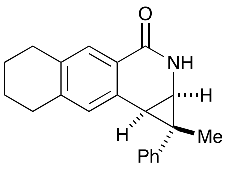

3k

General procedure A, *O*-Piv arylhydroxamate **1k** (0.1 mmol, 27.5 mg) reacted with cyclopropene **2c** (0.11 mmol, 14.3 mg) gives the desired dihydroisoquinolone (8.5:1 regioselectivity, >20:1 dr of the crude reaction mixture). The mixture of product (8.5:1 regioselectivity, >20:1 dr) was obtained after purification by column chromatography using 1/3 to 2/1 EtOAc/hexane as an off-white solid (28.6 mg, 94 % yield).

^{1}H-NMR ($CDCl_3$, 400 MHz): δ 7.90 (s, 1H), 7.38-7.32 (m, 4H), 7.25-7.22 (m, 1H), 7.11 (s, 1H), 3.45 (dd, J = 8.0, 4.0 Hz, 1H), 2.81 (m, 4H), 2.64 (d, J = 8.0 Hz, 1H), 1.82 (m, 4H), 1.13 (s, 3H)

^{13}C- NMR ($CDCl_3$, 100 MHz): δ 164.7, 145.1, 142.4, 136.2, 133.1, 129.9, 128.7, 128.6, 126.8, 126.7, 126.3, 40.7, 29.5, 29.0, 26.5, 24.6, 23.0, 22.9, 13.1

IR (neat, cm^{-1}) 3190, 2926, 1660, 1613, 1445, 909, 731

LRMS m/z (ESI+APCI) calcd for $C_{21}H_{21}NO$ [M+H]: 303.2; Found: 303.2

3l+3l'

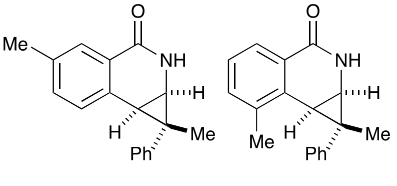

3l 3l'

General procedure A, O-Piv arylhydroxamate **1l** (0.1 mmol, 23.5 mg) reacted with cyclopropene **2c** (0.11 mmol, 14.3 mg) gives the desired dihydroisoquinolone (3:1 regioselectivity, >20:1 dr of the crude reaction mixture). The mixture of products (3:1 regioselectiviy, >20:1 dr) was obtained after purification by column chromatography using 1/3 to 2/1 EtOAc/hexane as an off-white solid (25.5 mg, 92 % yield).

^{1}H-NMR ($CDCl_3$, 400 MHz): (See spectra)

^{13}C- NMR ($CDCl_3$, 100 MHz): (See spectra)

IR (neat, cm^{-1}) 3196, 3025, 2923, 1665, 1613, 1500

LRMS m/z (ESI+APCI) calcd for $C_{18}H_{17}NO$ [M+H]: 264.1; Found: 264.1

3m+3m'

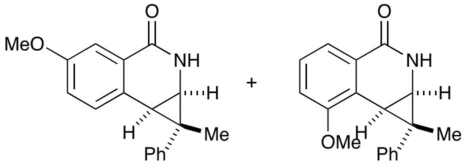

3m 3m'

General procedure A, O-Piv arylhydroxamate **1m** (0.1 mmol, 25.1 mg) reacted with cyclopropene **2c** (0.11 mmol, 14.3 mg) gives the desired dihydroisoquinolone (1:1 regioselectivity, >20:1 dr of the crude reaction mixture). The mixture of product was obtained

after purification by column chromatography using 1/3 to 2/1 EtOAc/hexane as an off-white solid (25.1 mg, 86 % yield).

^{1}H-NMR ($CDCl_3$, 400 MHz): (See spectra)

^{13}C- NMR ($CDCl_3$, 100 MHz): (See spectra)

IR (neat, cm^{-1}) 3187, 2932, 1668, 1581, 1494, 1263, 1057, 1032, 752

LRMS m/z (ESI+APCI) calcd for $C_{18}H_{17}NO_2$ [M+H]: 280.1; Found: 280.1

4.2 Cyclcopropene scope (Table 3)

(1*S*,1a*R*,7b*R*)-1-methyl-1-(*p*-tolyl)-1,1a,2,7b-tetrahydro-3*H*-cyclopropa[*c*]isoquinolin-3-one (4a)

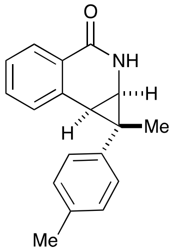

4a

General procedure B, *O*-Boc arylhydroxamate **1d** (0.1 mmol, 23.7 mg) reacted with cyclopropene **2d** (0.11 mmol, 15.9 mg) gives the desired dihydroisoquinolone (>20:1 dr of the crude reaction mixture). The major diastereomer was obtained after purification by column chromatography using 1/3 to 2/1 EtOAc/hexane as an off-white solid (15.3 mg, 58 % yield).

^{1}H-NMR ($CDCl_3$, 400 MHz): δ 8.19 (d, J = 8.0 Hz, 1H), 7.50 (t, J = 8.0 Hz, 1H), 7.42 (d, J = 8.0 Hz, 1H), 7.36 (t, J = 8.0 Hz, 1H), 7.24-7.15 (m, 4H), 6.91 (br. s, 1H), 3.45 (dd, J = 8.0, 4.0 Hz, 1H), 2.68 (d, J = 8.0 Hz, 1H), 2.35 (s, 3H), 1.11 (s, 3H)

^{13}C- NMR ($CDCl_3$, 100 MHz): δ 164.1, 141.8, 136.5, 136.2, 132.4, 129.44, 129.39, 128.2, 126.87, 126.85, 126.7, 40.6, 26.6, 24.7, 21.0, 13.2

IR (neat, cm^{-1}) 3188, 3028, 2919, 1662, 1597, 1479, 1341, 777

LRMS m/z (ESI+APCI) calcd for $C_{18}H_{17}NO$ [M+H]: 264.1; Found: 264.2

(1*S*,1a*R*,7b*R*)-1-(4-methoxyphenyl)-1-methyl-1,1a,2,7b-tetrahydro-3*H*-cyclopropa[*c*]isoquinolin-3-one (4b)

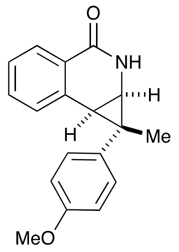

4b

General procedure B, *O*-Boc arylhydroxamate **1d** (0.1 mmol, 23.7 mg) reacted with cyclopropene **2e** (0.11 mmol, 17.6 mg) gives the desired dihydroisoquinolone (>20:1 dr of the crude reaction mixture). The major diastereomer was obtained after purification by column chromatography using 1/3 to 2/1 EtOAc/hexane as an off-white solid (18.7 mg, 67 % yield).

^{1}H-NMR ($CDCl_3$, 400 MHz): δ 8.19 (d, J = 8 Hz, 1H), 7.50 (t, J = 8 Hz, 1H), 7.42 (d, J = 8 Hz, 1H), 7.36 (t, J = 8 Hz, 1H), 7.27 (d, J = 8 Hz, 2H), 7.11 (brs, 1H), 6.90 (d, J = 8 Hz, 2H), 3.81 (s, 3H), 3.44 (dd, J = 4, 8 Hz, 1H), 2.65 (d, J = 8 Hz, 1H), 1.09 (s, 3H)

^{13}C- NMR ($CDCl_3$, 100 MHz): δ 164.2, 158.2, 137.0, 136.6, 132.4, 129.4, 128.1, 128.1, 126.8, 114.1, 55.3, 40.5, 26.4, 24.5, 13.6

IR (neat, cm^{-1}) 3196, 3039, 2929, 2830, 1662, 1513, 1240, 1178, 1030, 777, 737

LRMS m/z (ESI+APCI) calcd for $C_{18}H_{17}NO_2$ [M+H]: 280.1; Found: 280.1

(1*S*,1a*R*,7b*R*)-1-(4-bromophenyl)-1-methyl-1,1a,2,7b-tetrahydro-3*H*-cyclopropa[*c*]isoquinolin-3-one (4c)

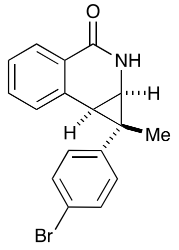

4c

General procedure B, *O*-Boc arylhydroxamate **1d** (0.1 mmol, 23.7 mg) reacted with cyclopropene **2f** (0.11 mmol, 23.0 mg) gives the desired dihydroisoquinolone (>20:1 dr of the crude reaction mixture). The major diastereomer was obtained after purification by column chromatography using 1/3 to 2/1 EtOAc/hexane as an off-white solid (17.1 mg, 52 % yield).

^{1}H-NMR ($CDCl_3$, 400 MHz): δ 8.19 (d, *J* = 8.0 Hz, 1H), 7.51 (t, *J* = 8.0 Hz, 1H), 7.48 (d, *J* = 8.0 Hz, 2H), 7.42-7.34 (m, 2H), 7.20 (d, *J* = 8.0 Hz, 2H), 7.10 (br. s, 1H), 6.90 (d, *J* = 8.0 Hz, 2H), 3.46 (dd, *J* = 8.0, 4.0 Hz, 1H), 2.67 (d, *J* = 8.0 Hz, 1H), 1.10 (s, 3H)

^{13}C- NMR ($CDCl_3$, 100 MHz): 164.0, 143.9, 136.0, 132.5, 131.8, 129.4, 128.6, 128.2, 127.1, 126.8, 120.3, 40.6, 26.7, 24.6, 13.0

IR (neat, cm^{-1}) 3171, 3028, 2921, 1662, 1490, 1437

LRMS m/z (ESI+APCI) calcd for $C_{17}H_{14}BrNO$ [M+H]: 328.0, 330.0; Found: 328.0, 330.1

(1*S*,1a*R*,7b*R*)-1-(3-methoxyphenyl)-1-methyl-1,1a,2,7b-tetrahydro-3*H*-cyclopropa[*c*]isoquinolin-3-one (4d)

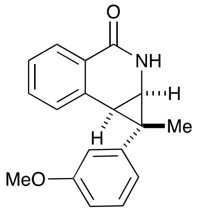

4d

General procedure B, *O*-Boc arylhydroxamate **1d** (0.1 mmol, 23.7 mg) reacted with cyclopropene **2g** (0.11 mmol, 17.6 mg) gives the desired dihydroisoquinolone (13.5:1 dr of the crude reaction mixture). The major diastereomer was obtained after purification by column chromatography using 1/3 to 2/1 EtOAc/hexane as an off-white solid (16.2 mg, 58 % yield).

^{1}H-NMR ($CDCl_3$, 400 MHz): δ 8.20 (d, *J* = 8.0 Hz, 1H), 7.50 (t, *J* = 8.0 Hz, 1H), 7.41 (d, *J* = 8.0 Hz, 1H), 7.36 (t, *J* = 8.0 Hz, 1H), 7.30 (t, *J* = 8.0 Hz, 1H), 6.92 (d, *J* = 8.0 Hz, 1H), 6.88 (s, 1H), 6.80 (d, *J* = 8.0 Hz, 1H), 3.84 (s, 3H), 3.45 (dd, *J* = 8.0, 4.0 Hz, 1H), 2.71 (d, *J* = 8.0 Hz, 1H), 1.12 (s, 3H)

^{13}C- NMR ($CDCl_3$, 100 MHz): δ 164.1, 159.9, 146.5, 136.3, 132.5, 129.7, 129.5, 128.2, 126.95, 126.89, 119.0, 113.2, 111.2, 55.3, 40.8, 26.8, 24.9, 13.0

IR (neat, cm^{-1}) 2952, 1633, 1560, 1482, 1341, 1291, 1039, 773, 699

LRMS m/z (ESI+APCI) calcd for $C_{18}H_{17}NO_2$ [M+H]: 280.1; Found: 280.1

(1*S*,1a*R*,7b*R*)-1-methyl-1-(naphthalen-2-yl)-1,1a,2,7b-tetrahydro-3*H*-cyclopropa[*c*]isoquinolin-3-one (4e)

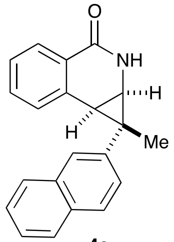

4e

General procedure B, *O*-Boc arylhydroxamate **1d** (0.1 mmol, 23.7 mg) reacted with cyclopropene **2h** (0.11 mmol, 19.8 mg) gives the desired dihydroisoquinolone (>20:1 dr of the crude reaction mixture). The major diastereomer was obtained after purification by column chromatography using 1/3 to 2/1 EtOAc/hexane as an off-white solid (23.3 mg, 78% yield).

^{1}H-NMR ($CDCl_3$, 400 MHz): δ 8.23 (d, J = 8 Hz), 7.87-7.79 (m, 4H), 7.55-7.45 (m, 5H), 7.39 (t, J = 8 Hz, 1H), 6.99 (brs, 1H), 3.61 (dd, J = 4, 8 Hz, 1H), 2.82 (d, J = 8 Hz, 1H), 1.22 (s, 3H)

^{13}C- NMR ($CDCl_3$, 100 MHz): δ 164.1, 142.1, 136.4, 133.4, 132.5, 132.1, 129.5, 128.6, 128.2, 127.6, 127.0, 126.9, 126.4, 125.8, 125.38, 125.36, 40.5, 26.5, 25.4, 13.4

IR (neat, cm^{-1}) 3185, 3042, 2924, 1665, 1600, 777

LRMS m/z (ESI+APCI) calcd for $C_{21}H_{17}NO$ [M+H]: 300.1; Found: 300.2

methyl (1*R*,1a*R*,7b*R*)-3-oxo-1-phenyl-1a,2,3,7b-tetrahydro-1*H*-cyclopropa[*c*]isoquinoline-1-carboxylate (4f)

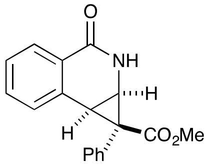

4f

General procedure B, *O*-Boc arylhydroxamate **1d** (0.1 mmol, 23.7 mg) reacted with cyclopropene **2b** (0.11 mmol, 19.2 mg) gives the desired dihydroisoquinolone (8:1 dr of the crude reaction mixture). The major diastereomer (>10:1 dr purity) was obtained after purification by column chromatography using 1/3 to 2/1 EtOAc/hexane as an off-white solid (22.0 mg, 75% yield).

^{1}H-NMR ($CDCl_3$, 400 MHz): δ 8.19 (d, *J* = 8 Hz, 1 Hz), 7.56-7.50 (m, 2H), 7.48-7.30 (m, 5H), 3.48 (dd, *J* = 8.0, 4.0 Hz), 3.36 (s, 3H), 3.11 (d, *J* = 8.0 Hz, 1H)

^{13}C- NMR ($CDCl_3$, 100 MHz): δ 167.5, 163.8, 138.0, 134.2, 133.4, 132.5, 129.2, 129.1, 128.8, 127.9, 127.7, 127.0, 52.2, 41.7, 35.6, 28.2

IR (neat, cm^{-1}) 3057, 2950, 1726, 1670, 1445, 909, 731

LRMS m/z (ESI+APCI) calcd for $C_{18}H_{15}NO_3$ [M+H]: 293.1; Found: 293.1

NOESY

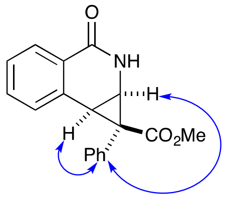

4f

(1*S*,1a*R*,7b*R*)-1a,3',4',7b-tetrahydro-2'*H*-spiro[cyclopropa[*c*]isoquinoline-1,1'-naphthalen]-3(2*H*)-one (4g)

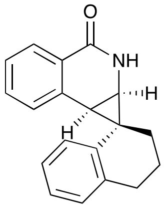

4g

General procedure B, *O*-Boc arylhydroxamate **1d** (0.1 mmol, 23.7 mg) reacted with cyclopropene **2i** (0.11 mmol, 17.2 mg) gives the desired dihydroisoquinolone (>20:1 dr of the crude reaction mixture). The major diastereomer was obtained after purification by column chromatography using 1/3 to 2/1 EtOAc/hexane as an off-white solid (19.8 mg, 72% yield).

^{1}H-NMR ($CDCl_3$, 400 MHz): δ 8.18 (d, J = 8.0 Hz, 1H), 7.47 (t, J = 8.0 Hz, 1H), 7.36 (t, J = 8.0 Hz, 1H), 7.32 (d, J = 8.0 Hz, 1H), 7.24-7.12 (m, 3H), 6.79 (br. s, NH), 6.68 (d, J = 8.0 Hz), 3.50 (dd, J = 8.0, 4.0 Hz), 2.83 (t, J = 8.0 Hz, 2H), 2.78 (d, J = 8.0 Hz, 1H), 1.75-1.55 (m, 2H), 1.35-1.26 (m, 2H)

^{13}C- NMR ($CDCl_3$, 100 MHz): δ 164.0, 139.1, 137.9, 136.0, 132.5, 129.5, 129.0, 128.1, 127.04, 127.00, 126.6, 125.3, 119.6, 43.7, 30.7, 30.2, 22.9, 21.6, 21.3

IR (neat, cm^{-1}) 2920, 1657, 1598, 1479, 1344, 751

LRMS m/z (ESI+APCI) calcd for $C_{19}H_{17}NO$ [M+H]: 275.1; Found: 276.1

(1*R*,1a*R*,7b*R*)-1-benzyl-1-methyl-1,1a,2,7b-tetrahydro-3*H*-cyclopropa[*c*]isoquinolin-3-one (4h)

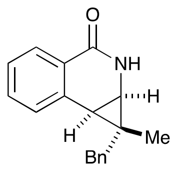

4h

General procedure B, *O*-Boc arylhydroxamate **1d** (0.1 mmol, 23.7 mg) reacted with cyclopropene **2j** (0.11 mmol, 15.9 mg) gives the desired dihydroisoquinolone (2.3:1 dr of the crude reaction mixture). The mixture of product was obtained after purification by column chromatography using 1/3 to 2/1 EtOAc/hexane as an off-white solid (16.8 mg, 64% yield).

^{1}H-NMR ($CDCl_3$, 400 MHz): (See spectra)

^{13}C- NMR ($CDCl_3$, 100 MHz): (See spectra)

LRMS m/z (ESI+APCI) calcd for $C_{18}H_{17}NO$ [M+H]: 264.1; Found: 264.1

5. Mechanistic studies

5.1 C-H activation reversibility

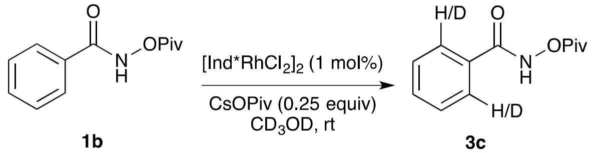

A 1-dram vial was charged with a stir bar, *O*-pivaloyl benzhydroxamate (0.1 mmol, 1 equiv., 22.1 mg), [Ind*$RhCl_2$]$_2$ (0.001 mmol, 1 mol%, 0.7 mg) and CsOPiv (0.025 mmol, 0.25 equiv., 5.9 mg) were weighed. The mixture was dissolved in CD_3OD (0.1 M, 1 mL) and stirred for 1 min. The mixture was determined the deuterium incorporation by ^{1}H-NMR spectroscopy using 1,3,5-trimethoxybenzene as an internal standard.

Another vial was stirred for 24 h at room temperature.

5.2 Electronic preference of the reaction

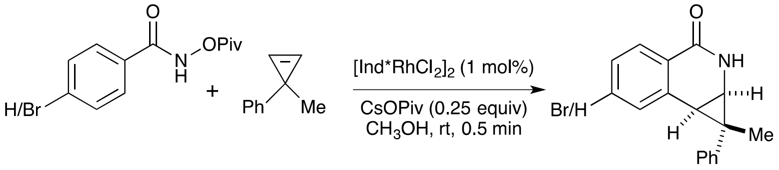

A 1-dram vial charged with a stir bar, *O*-pivaloyl benzhydroxamate (0.05 mmol, 0.5 equiv., 11.1 mg), *O*-pivaloyl p-bromo benzhydroxamate (0.05 mmol, 0.5 equiv., 15.0 mg), $[Ind^{*}RhCl_2]_2$ (0.001 mmol, 1 mol%, 0.7 mg) and CsOPiv (0.025 mmol, 0.25 equiv., 5.9 mg) were weighed. The mixture was dissolved in CH_3OH and stirred for 30 sec at 0°C. Then, cyclopropene (0.1 mmol, 0.5 equiv., 5.9 mg) was added and stirred for 30 sec at 0°C. The reaction was quenched using satd. $NaHCO_3$ and extracted 3 times with EtOAc. The combined organic layers were washed with brine, dried over $MgSO_4$, filtered and solvent was evaporated. The crude mixture was characterized by ^{1}H-NMR spectroscopy using 1,3,5-trimethoxybenzene as an internal standard.

5.3 Kinetic isotope study

D_5-benzoic acid was prepared according to the reported procedure.[13] Then, *O*-pivaloyl D_5-benzhydroxamate was prepared.

O-pivaloyl D_5-benzhydroxamate (0.1 mmol, 0.1 equiv., 22.6 mg), cyclopropene (0.11 mmol, 0.11 equiv., 14.3 mg), $[Ind^{*}RhCl_2]_2$ (0.001 mmol, 1 mol%, 0.7 mg) and CsOPiv (0.025 mmol, 0.25 equiv., 5.9 mg) were weighed in a dram vial. The reaction mixture was stirred at room temperature for 16 h. The major diastereomer was obtained after purification by column chromatography using 1/3 to 2/1 EtOAc/hexane as an off-white solid (82% yield, >20:1 dr).

^{1}H-NMR ($CDCl_3$, 400 MHz): δ 7.37-7.31 (m, 4H), 7.23 (t, J = 8.0 Hz, 1H), 3.45 (dd, J = 8.0, 4.0 Hz, 1H), 2.70 (d, J = 8.0 Hz), 1.11 (s, 3H)

LRMS m/z (ESI+APCI) calcd for $C_{17}H_{11}D_4NO$ [M+H]: 254.1; Found: 254.1

[13] Chioung, H. A.; Pham, Q.-N.; Daugulis, O. *J. Am. Chem. Soc.*, **2007**, *129*, 9879

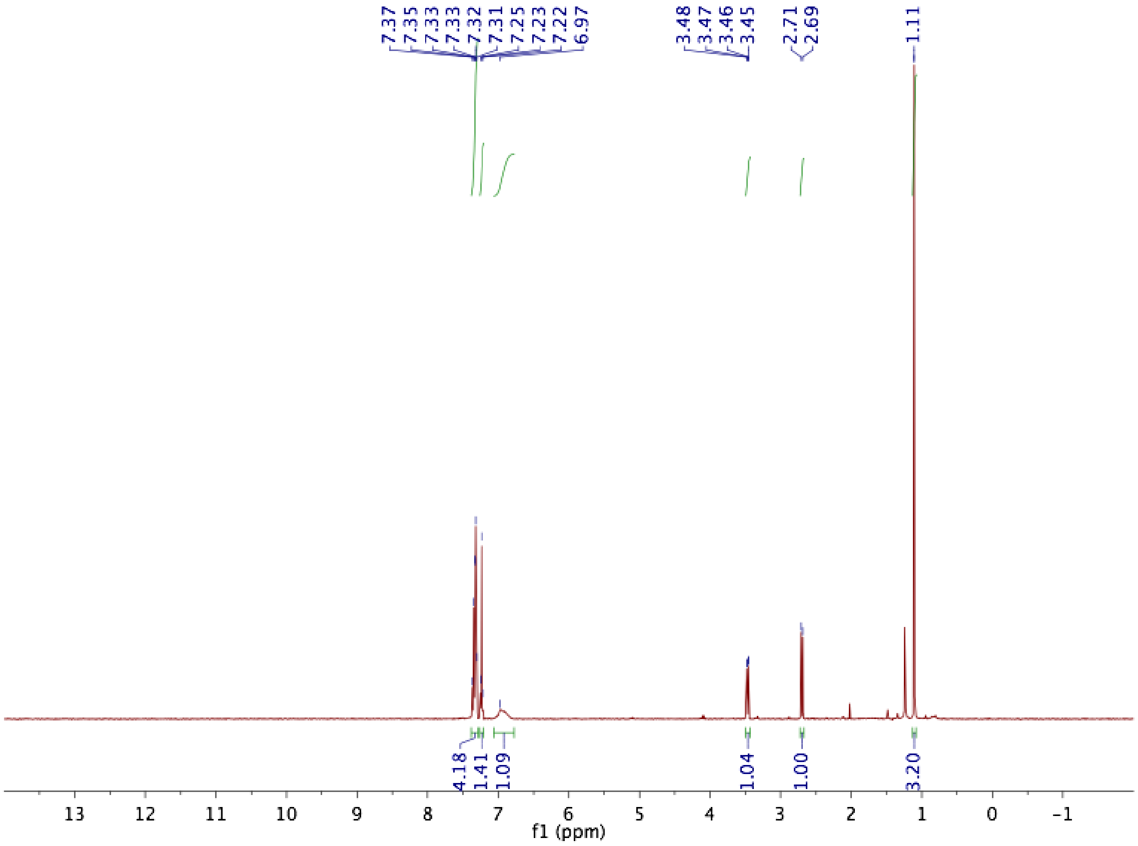

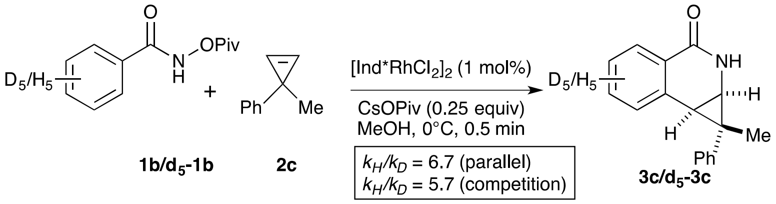

Parallel experiment: Two 1-dram vials were charged with a stir bar, proteo- and deutero-benzamide substrate (0.1 mmol, 1 equiv), [Ind*$RhCl_2$]$_2$ (1 mol%) and CsOPiv (0.25 equiv) were weighed. The mixture was dissolved in CD_3OD (0.1 M, 1 mL) and stirred for 30 sec at 0°C. Then, cyclopropene (0.1 mmol, 0.5 equiv., 5.9 mg) was added and stirred for 30 sec. The reaction was quenched using satd. $NaHCO_3$ and extracted 3 times with EtOAc. The combined organic layers were washed with brine, dried over $MgSO_4$, filtered and solvent was evaporated. The crude mixture was characterized by ^{1}H-NMR spectroscopy using 1,3,5-trimethoxybenzene as an internal standard.

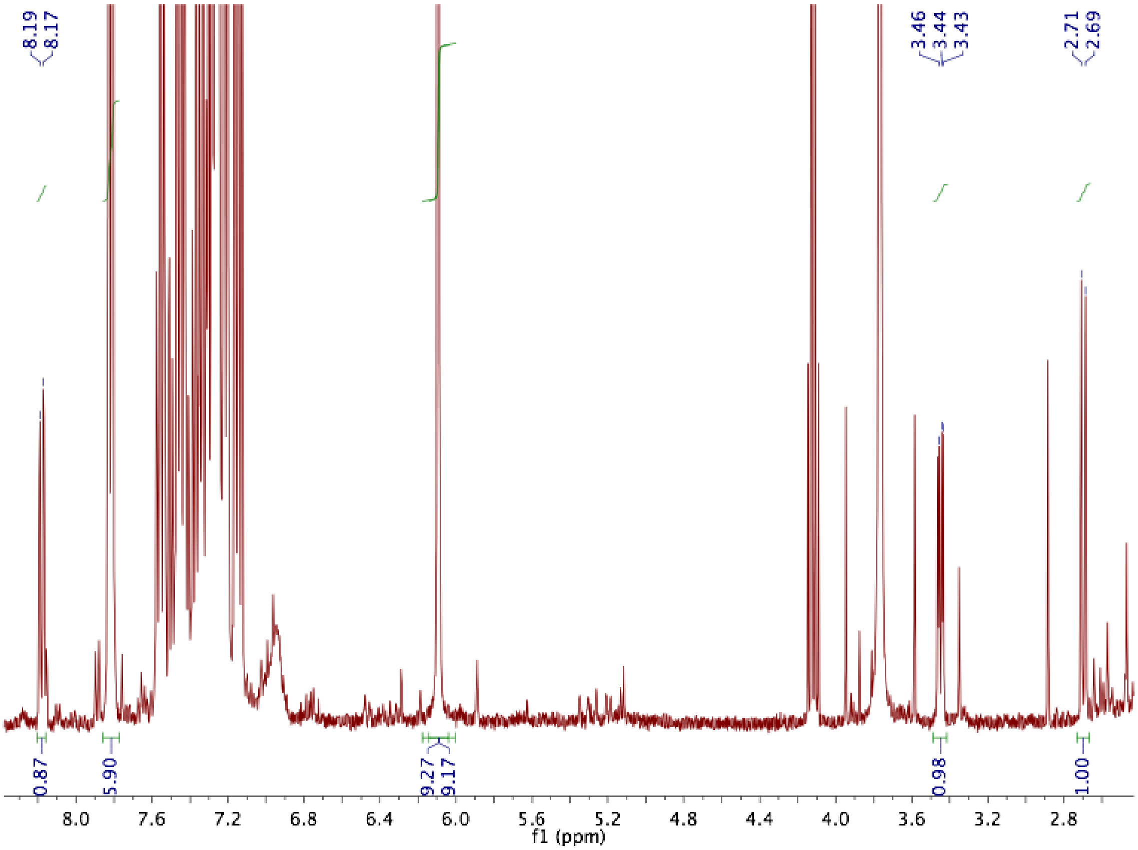

Competition experiment: A 1-dram vial were charged with a stir bar, proteo- and deutero-benzamide substrate (0.05 mmol, 0.5 equiv), [Ind*$RhCl_2$]$_2$ (0.001 mmol, 1 mol%, 0.7 mg) and CsOPiv (0.025, 0.25 equiv., 5.9 mg) were weighed. The mixture was dissolved in CD_3OD (0.1 M, 1 mL) and stirred for 30 sec at 0°C. Then, cyclopropene (0.1 mmol, 0.5 equiv., 5.9 mg) was added and stirred for 30 sec. The reaction was quenched using satd. $NaHCO_3$ and extracted 3 times with EtOAc. The combined organic layers were washed with brine, dried over $MgSO_4$, filtered and solvent was evaporated. The crude mixture was characterized by ^{1}H-NMR spectroscopy using 1,3,5-trimethoxybenzene as an internal standard.

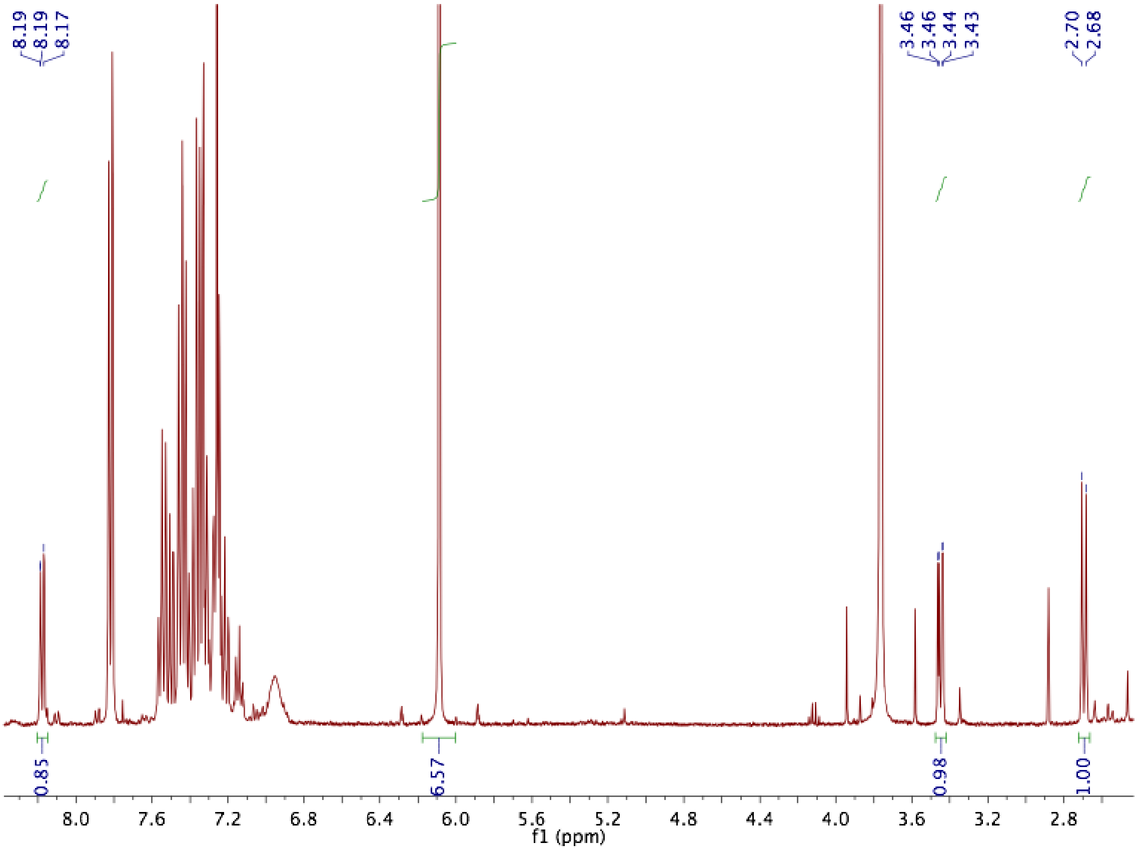
8.19
8.19
8.17
3.46
3.46
3.44
3.43
2.70
2.68
0.85
6.57
0.98
1.00
8.0
7.6
7.2
6.8
6.4
6.0
5.6
5.2
4.8
4.4
4.0
3.6
3.2
2.8
f1 (ppm)

5.4 Epimerization study

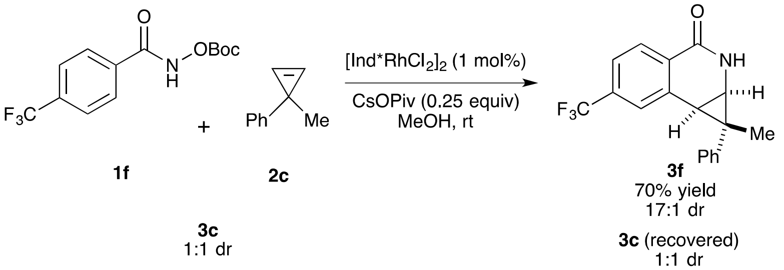

A diastereomeric mixture of dihydroisoquinolone **3c** (1:1 dr), substrate **1e** (0.1 mmol, 1 equiv., 30.2 mg), [ind*$RhCl_2$]$_2$ (0.001, 1 mol%, 0.8 mg) CsOPiv (0.025 mmol, 0.25 equiv., 5.9 mg) were weighed in a dram vial charged with a stir bar. MeOH (1mL, 0.1 M) was added and the mixture was stirred for 30 seconds and cyclopropene **2c** (0.11 mmol, 14.9 µL, 1.1 equiv to **1e**) was then added. The reaction was stirred at room temperature for 16 hours and the starting material **1e** was monitored that it was consumed monitoring by TLC. The reaction was quenched using satd. $NaHCO_3$ and extracted 3 times with EtOAc. The combined organic layers were washed with brine, dried over $MgSO_4$, filtered and the solvent was evaporated. The crude mixture was characterized by ^{1}H-NMR spectroscopy using 1,3,5-trimethoxybenzene as an internal standard.

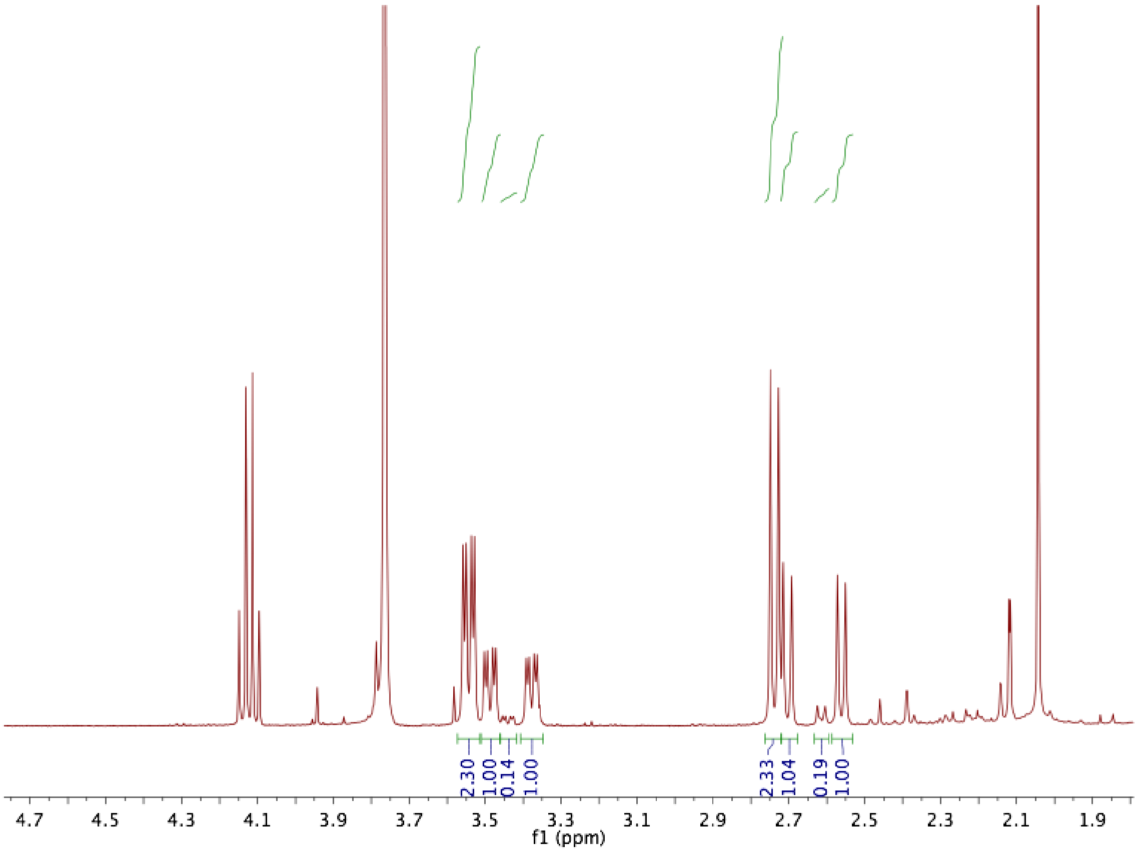

5.5 The role of an acyl directing group

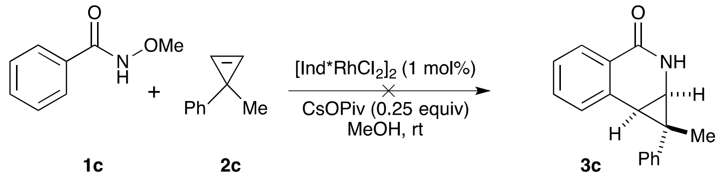

1c 2c 3c

6. Derivatizations of dihydroisoquinolone products

(1*S*,1a*R*,7b*R*)-3-chloro-1-methyl-1-phenyl-1a,7b-dihydro-1*H*-cyclopropa[*c*]isoquinoline (5a)

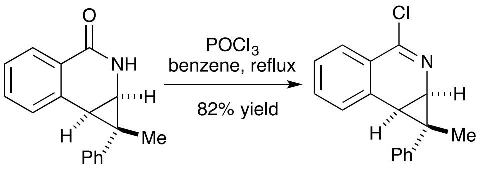

3c 5a

To a flamed dried round-bottom flask equipped with a stir bar and reflux condenser, dihydroisoquinolone (0.39 mmol, 97 mg), dry benzene and $POCl_3$ were added. The reaction mixture was refluxed for 6 h. After the completion, the reaction mixture was evaporated under reduced pressure at 60°C and treated with 5% Et_3N/Et_2O at -30°C. After stirring for 10 minutes, the crude was passed through alumina column chromatography using 1:1 Et_2O/hexane as an eluent to give a crude imidoyl chloride as a white solid (85 mg, 82% yield).

^{1}H-NMR ($CDCl_3$, 400 MHz): δ 7.99 (d, *J* = 8.0 Hz, 1H), 7.55-7.45 (m, 4H), 7.42-7.36 (m, 3H), 7.28 (t, *J* = 8.0 Hz, 1H), 4.08 (d, *J* = 8.0 Hz, 1H), 2.75 (d, *J* = 8.0 Hz, 1H), 0.93 (s, 3H)

^{13}C- NMR ($CDCl_3$, 100 MHz): δ 151.9, 145.3, 136.2, 132.5, 129.2, 128.8, 127.9, 127.8, 127.0, 126.8, 125.0, 50.7, 31.4, 22.2, 13.4

IR (neat, cm^{-1}) 3024, 1614, 1599, 1567, 1218

LRMS m/z (ESI+APCI) calcd for $C_{17}H_{14}ClN$ [M+H]: 268.1; Found: 268.1

(1*S*,1a*R*,7b*R*)-1-methyl-1-phenyl-1a,7b-dihydro-1*H*-cyclopropa[*c*]isoquinolin-3-yl trifluoromethanesulfonate (5b)

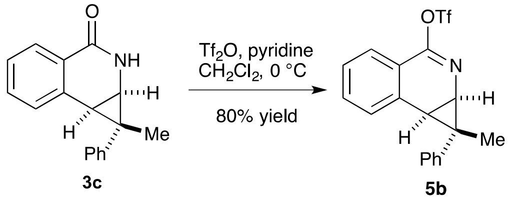

To a flamed-dried round equipped with a stir bar, dihydroisoquinolone (0.5 mmol, 125 mg) in CH_2Cl_2 (5 mL) was added. The reaction mixture was cooled at -0°C. Then, Tf_2O (0.77 mmol, 0.13 mL) and pyridine (0.765 mmol, 61 μL) was slowly added to the reaction mixture which was then stirred at the same temperature for 10 mins. The reaction was diluted with CH_2Cl_2 and washed with satd. $NaHCO_3$. The combined organic extracts were dried over $MgSO_4$ and concentrated to give a crude product. The crude was purified by alumina column chromatography using EtOAc as an eluent to give the desired product as a purple solid (152 mg, 80% yield).

^{1}H-NMR ($CDCl_3$, 400 MHz): δ 8.19 (d, J = 8.0 Hz, 1H), 7.66 (t, J = 8.0 Hz, 1H), 7.53 (t, J = 8 Hz, 3H), 7.47-7.38 (m, 3H), 7.30 (t, J = 8.0 Hz, 1H), 4.05 (d, J = 8.0 Hz, 1H), 2.86 (d, J = 8.0 Hz, 1H), 1.23 (s, 3H)

^{13}C- NMR ($CDCl_3$, 100 MHz): δ 161.7, 143.4, 136.1, 134.8, 129.8, 129.7, 129.0, 128.0, 127.9, 127.5, 125.4, 121.2 (q), 44.7, 29.2, 25.9, 15.5

^{19}F- NMR ($CDCl_3$, 381 MHz): 72.3

IR (neat, cm^{-1}) 3061, 1078, 1603, 1492, 1466, 1291, 1093, 792, 720, 688

LRMS m/z (ESI+APCI) calcd for $C_{18}H_{14}F_3NO_3S$ [M+H]: 382.1; Found: 382.1

7. X-ray Structure of 3m

Rovis227-1 (CCDC 1472771)

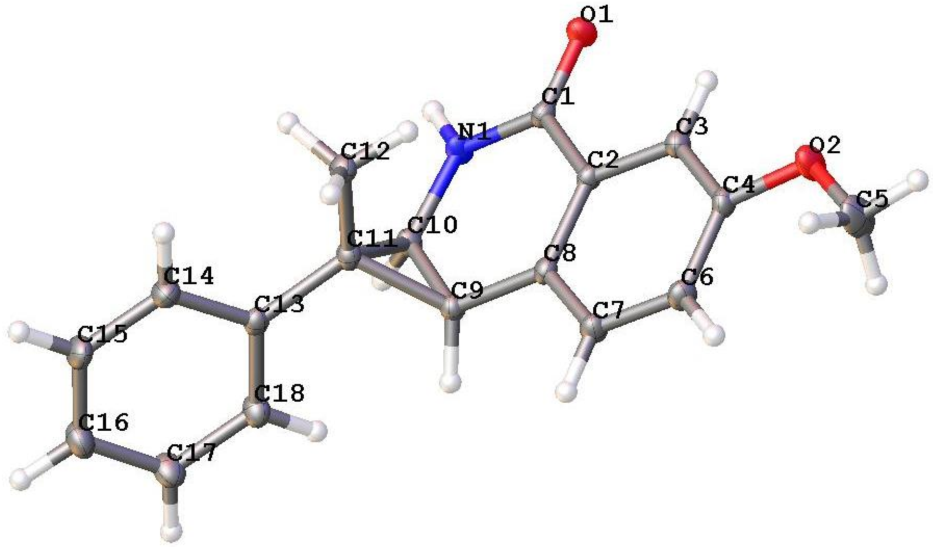

Table 1 Crystal data and structure refinement for Rovis227-1.

Identification code	Rovis227-1
Empirical formula	$C_{18}H_{17}NO_2$
Formula weight	279.32
Temperature/K	99.77
Crystal system	monoclinic
Space group	$P2_1/n$
a/Å	8.5035(2)
b/Å	7.1303(2)
c/Å	23.6793(6)
α/°	90
β/°	95.7420(12)
γ/°	90
Volume/Å^3	1428.53(6)
Z	4
ρ_{calc}g/cm^3	1.299
μ/mm^{-1}	0.085
F(000)	592.0

Crystal size/mm^3	0.25 × 0.124 × 0.107
Radiation	MoKα (λ = 0.71073)
2Θ range for data collection/°	5.97 to 66.776
Index ranges	-13 ≤ h ≤ 12, -7 ≤ k ≤ 10, -36 ≤ l ≤ 36
Reflections collected	30307
Independent reflections	5464 [R_{int} = 0.0926, R_{sigma} = 0.0945]
Data/restraints/parameters	5464/0/192
Goodness-of-fit on F^2	1.033
Final R indexes [I>=2σ (I)]	R_1 = 0.0685, wR_2 = 0.1558
Final R indexes [all data]	R_1 = 0.1350, wR_2 = 0.1811
Largest diff. peak/hole / e Å^{-3}	0.54/-0.29

Table 2 Fractional Atomic Coordinates (×10^4) and Equivalent Isotropic Displacement Parameters (Å^2×10^3) for Rovis227-1. U_{eq} is defined as 1/3 of of the trace of the orthogonalised U_{IJ} tensor.

**Atom**	***x***	***y***	***z***	**U(eq)**
O1	6707.2(13)	3443.8(17)	54.8(5)	18.0(3)
O2	9731.6(14)	-2398.8(19)	511.4(6)	23.2(3)
N3	4408.4(16)	3146(2)	448.1(6)	15.5(3)
C4	7791.1(18)	-66(2)	452.3(7)	15.0(3)
C5	6334.4(18)	637(2)	575.3(6)	13.0(3)
C6	5347.7(18)	-415(2)	898.4(6)	13.7(3)
C7	3777.6(18)	313(2)	1014.8(6)	13.6(3)
C8	3308.6(18)	2188(2)	768.8(7)	14.4(3)
C9	5837.4(18)	2508(2)	341.3(7)	13.5(3)
C10	8283.4(19)	-1830(2)	653.3(7)	15.8(3)
C11	3616.3(18)	2012(2)	1415.3(7)	14.6(3)
C12	2168.9(18)	2069(2)	1732.1(7)	14.2(3)
C13	7307.4(19)	-2900(3)	968.6(7)	17.5(3)
C14	5853.7(19)	-2186(2)	1085.9(7)	16.7(3)
C15	5107.6(19)	2856(3)	1709.6(7)	17.1(3)

C16	765.3(19)	1151(3)	1531.6(7)	18.4(4)
C17	2171(2)	3097(3)	2237.5(8)	19.7(4)
C18	-578(2)	1240(3)	1825.7(8)	23.0(4)
C19	-552(2)	2286(3)	2321.1(8)	24.0(4)
C20	826(2)	3196(3)	2525.4(8)	23.1(4)
C21	10328(2)	-4149(3)	736.9(10)	31.4(5)

Table 3 Anisotropic Displacement Parameters (Å^2×10^3) for Rovis227-1. The Anisotropic displacement factor exponent takes the form: $-2\pi^2[h^2a^{*2}U_{11}+2hka^*b^*U_{12}+\ldots]$.

**Atom**	**U_{11}**	**U_{22}**	**U_{33}**	**U_{23}**	**U_{13}**	**U_{12}**
O1	18.0(6)	14.9(6)	21.8(6)	4.6(5)	5.8(5)	2.3(5)
O2	19.8(6)	20.0(7)	31.6(7)	5.7(5)	10.4(5)	7.9(5)
N3	17.4(6)	12.7(7)	16.8(7)	6.0(5)	4.3(5)	4.8(5)
C4	14.7(7)	14.6(8)	16.0(7)	-1.0(6)	2.9(6)	0.7(6)
C5	15.9(7)	10.4(8)	12.8(7)	0.5(6)	1.5(6)	1.2(6)
C6	15.9(7)	12.2(8)	13.1(7)	-2.1(6)	2.0(6)	1.0(6)
C7	14.0(7)	13.2(8)	13.8(7)	0.3(6)	2.4(6)	1.0(6)
C8	14.7(7)	14.4(8)	14.3(7)	2.5(6)	3.5(5)	2.7(6)
C9	16.1(7)	10.8(8)	13.6(7)	-0.8(6)	1.8(6)	1.1(6)
C10	14.7(7)	15.7(9)	17.2(8)	-2.0(6)	2.8(6)	3.3(6)
C11	16.3(7)	14.5(8)	13.3(7)	0.7(6)	2.9(6)	1.9(6)
C12	16.9(7)	11.9(8)	14.1(7)	0.6(6)	2.7(6)	2.1(6)
C13	19.9(8)	13.8(8)	19.1(8)	2.6(7)	3.0(6)	3.2(7)
C14	19.1(7)	14.1(8)	17.5(8)	2.2(6)	5.0(6)	1.6(6)
C15	17.1(7)	17.8(9)	16.5(8)	-2.5(7)	2.9(6)	-0.7(7)
C16	18.0(8)	22.7(10)	14.7(8)	-2.7(7)	1.9(6)	1.2(7)
C17	19.7(8)	18.4(9)	21.5(8)	-4.2(7)	4.5(6)	-2.4(7)
C18	16.5(8)	29.6(11)	23.1(9)	-3.8(8)	2.4(7)	-0.6(7)
C19	20.8(8)	29.1(11)	23.4(9)	-2.4(8)	8.5(7)	1.6(8)
C20	26.5(9)	23.8(10)	20.4(9)	-7.9(7)	8.9(7)	-2.8(7)

C21	24.9(9)	26.1(11)	45.0(12)	8.2(10)	12.8(8)	14.1(8)

Table 4 Bond Lengths for Rovis227-1.

Atom	Atom	Length/Å	Atom	Atom	Length/Å
O1	C9	1.2461(19)	C7	C11	1.553(2)
O2	C10	1.3700(19)	C8	C11	1.532(2)
O2	C21	1.430(2)	C10	C13	1.398(2)
N3	C8	1.435(2)	C11	C12	1.505(2)
N3	C9	1.345(2)	C11	C15	1.510(2)
C4	C5	1.394(2)	C12	C16	1.402(2)
C4	C10	1.394(2)	C12	C17	1.403(2)
C5	C6	1.407(2)	C13	C14	1.390(2)
C5	C9	1.489(2)	C16	C18	1.397(2)
C6	C7	1.484(2)	C17	C20	1.391(2)
C6	C14	1.392(2)	C18	C19	1.388(3)
C7	C8	1.497(2)	C19	C20	1.384(3)

Table 5 Bond Angles for Rovis227-1.

Atom	Atom	Atom	Angle/°	Atom	Atom	Atom	Angle/°
C10	O2	C21	117.45(13)	O2	C10	C13	124.44(15)
C9	N3	C8	125.98(14)	C4	C10	C13	120.07(14)
C10	C4	C5	119.75(15)	C8	C11	C7	58.03(10)
C4	C5	C6	120.82(15)	C12	C11	C7	116.89(14)
C4	C5	C9	118.01(14)	C12	C11	C8	115.43(13)
C6	C5	C9	121.15(14)	C12	C11	C15	116.76(14)
C5	C6	C7	120.72(15)	C15	C11	C7	118.13(13)
C14	C6	C5	118.35(14)	C15	C11	C8	118.68(14)
C14	C6	C7	120.88(14)	C16	C12	C11	121.99(15)
C6	C7	C8	116.74(13)	C16	C12	C17	117.46(15)
C6	C7	C11	121.50(14)	C17	C12	C11	120.53(15)
C8	C7	C11	60.29(11)	C14	C13	C10	119.60(16)

N3	C8	C7	117.95(13)	C13	C14	C6	121.41(15)
N3	C8	C11	120.84(14)	C18	C16	C12	121.38(16)
C7	C8	C11	61.67(11)	C20	C17	C12	120.91(16)
O1	C9	N3	121.19(15)	C19	C18	C16	120.08(17)
O1	C9	C5	121.36(14)	C20	C19	C18	119.22(16)
N3	C9	C5	117.45(14)	C19	C20	C17	120.94(17)
O2	C10	C4	115.48(14)				

Table 6 Hydrogen Atom Coordinates (Å×10^4) and Isotropic Displacement Parameters (Å^2×10^3) for Rovis227-1.

Atom	*x*	*y*	*z*	U(eq)
H3	4122	4252	309	19
H4	8446	653	232	18
H7	2913	-640	1006	16
H8	2172	2310	618	17
H13	7635	-4109	1102	21
H14	5192	-2922	1299	20
H15A	5395	2196	2068	26
H15B	4930	4186	1787	26
H15C	5966	2735	1465	26
H16	726	455	1189	22
H17	3104	3734	2385	24
H18	-1509	584	1687	28
H19	-1470	2376	2518	29
H20	854	3899	2867	28
H21A	11371	-4387	609	47
H21B	9603	-5160	604	47
H21C	10420	-4100	1153	47

Experimental

Single crystals of $C_{18}H_{17}NO_2$ were obtained by the vapor diffusion method using CH_3Cl and pentane. A suitable crystal was selected and collected on a Bruker APEX-II CCD diffractometer. The crystal was kept at 99.77 K during data collection. Using Olex2[14], the structure was solved with the XS[15] structure solution program using Direct Methods and refined with the XL[16] refinement package using Least Squares minimisation.

Crystal structure determination of [Rovis227-1]

Crystal Data for $C_{18}H_{17}NO_2$ (M =279.32 g/mol): monoclinic, space group $P2_1/n$ (no. 14), a = 8.5035(2) Å, b = 7.1303(2) Å, c = 23.6793(6) Å, β = 95.7420(12)°, V = 1428.53(6) Å^3, Z = 4, T = 99.77 K, μ(MoKα) = 0.085 mm^{-1}, $Dcalc$ = 1.299 g/cm^3, 30307 reflections measured (5.97° ≤ 2Θ ≤ 66.776°), 5464 unique (R_{int} = 0.0926, R_{sigma} = 0.0945) which were used in all calculations. The final R_1 was 0.0685 (I > 2σ(I)) and wR_2 was 0.1811 (all data).

[14] Dolomanov, O.V., Bourhis, L.J., Gildea, R.J, Howard, J.A.K. & Puschmann, H. *J. Appl. Cryst.* **2009**, *42*, 339
[15] Sheldrick, G.M. *Acta Cryst.* **2008**, *A64*, 112
[16] Sheldrick, G.M. *Acta Cryst.* **2008**, *A64*, 112

8. Spectra

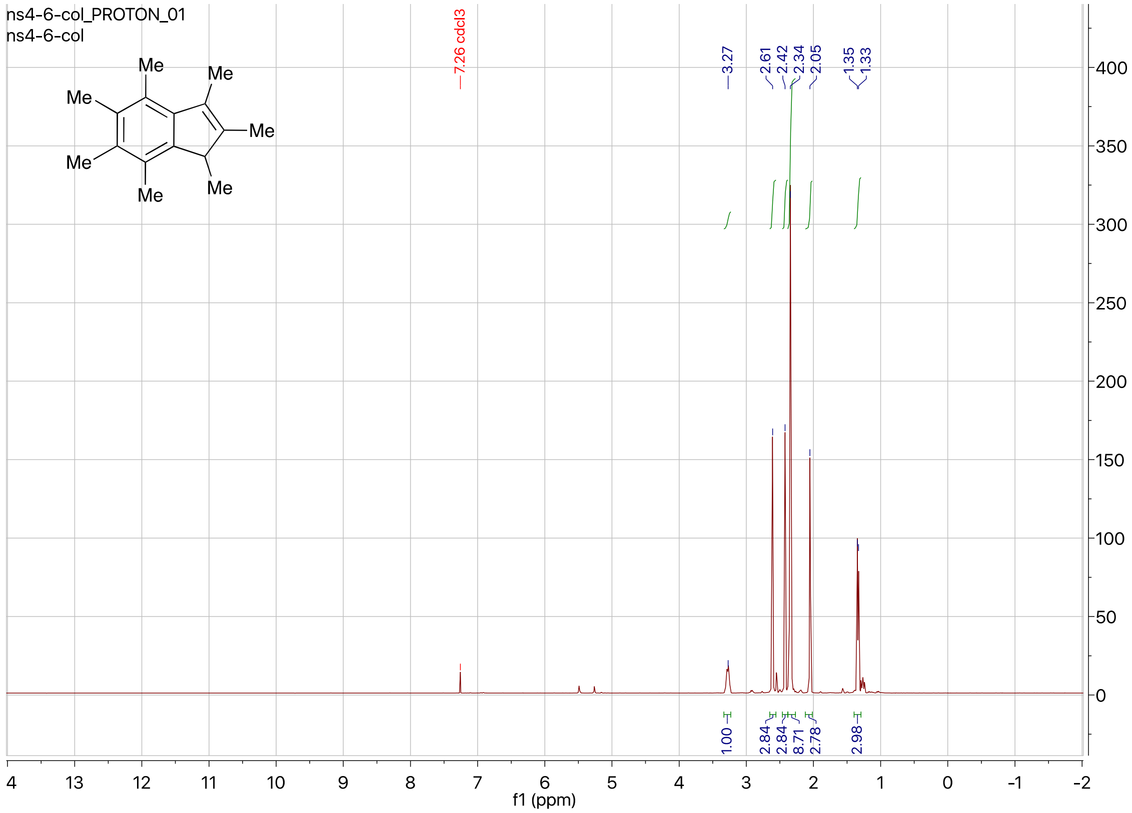
ns4-6-col_PROTON_01
ns4-6-col
Me
Me
Me
Me
Me
Me
Me
7.26 cdcl3
3.27
2.61
2.42
2.34
2.05
1.35
1.33
1.00
2.84
2.84
8.71
2.78
2.98
f1 (ppm)

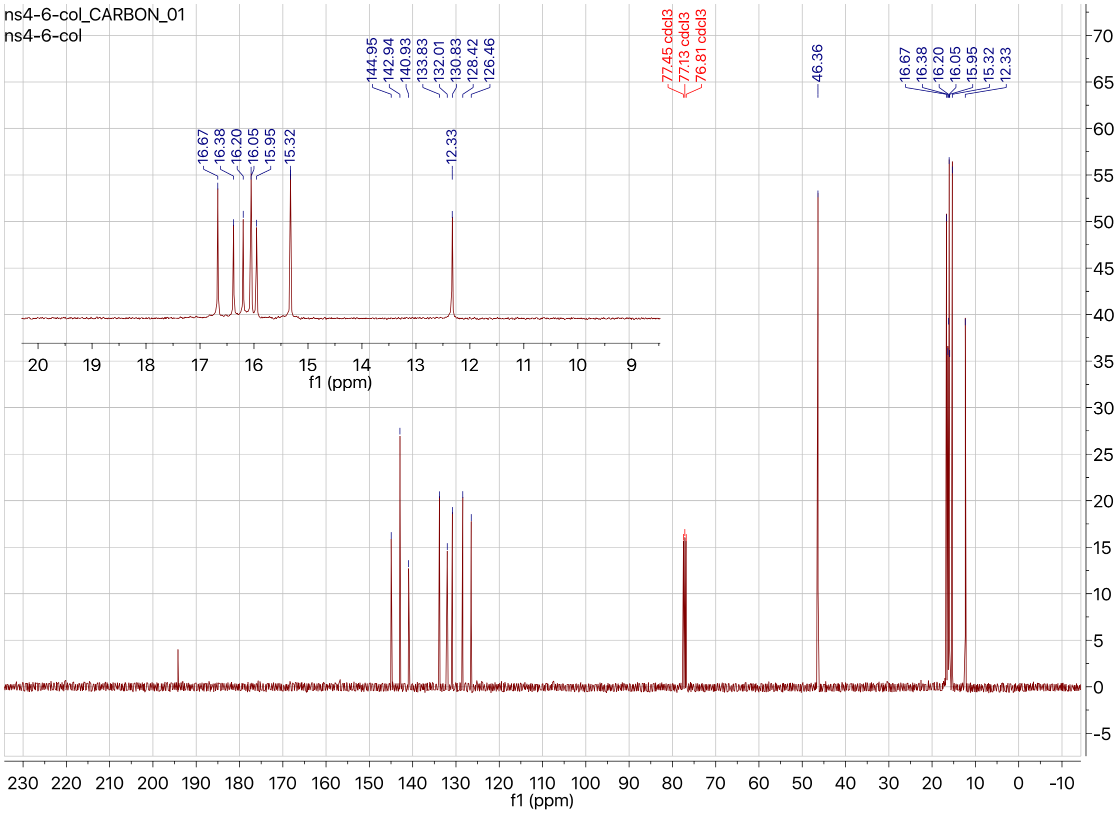
ns4-6-col_CARBON_01
ns4-6-col
144.95
142.94
140.93
133.83
132.01
130.83
128.42
126.46
77.45 cdcl3
77.13 cdcl3
76.81 cdcl3
46.36
16.67
16.38
16.20
16.05
15.95
15.32
12.33
f1 (ppm)

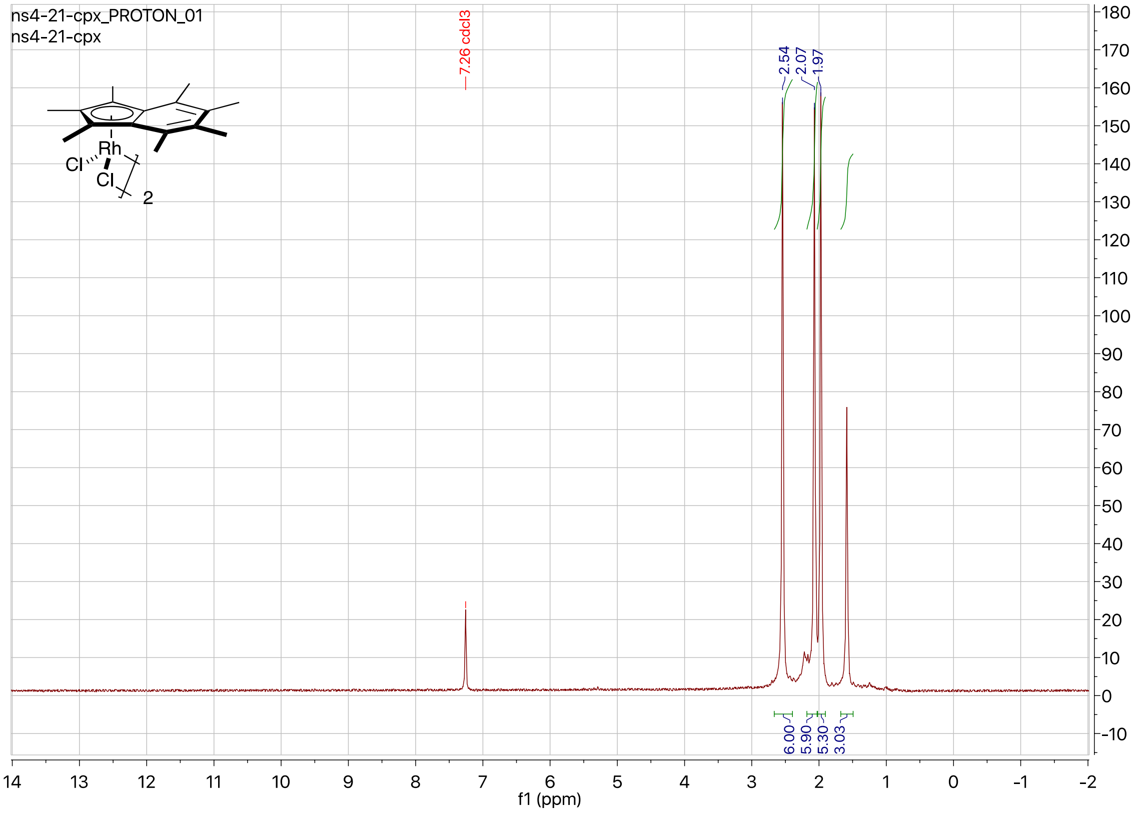

ns4-21-cpx_PROTON_01
ns4-21-cpx
Rh
Cl
Cl
2
7.26 cdcl3
2.54
2.07
1.97
6.00
5.90
5.30
3.03
f1 (ppm)

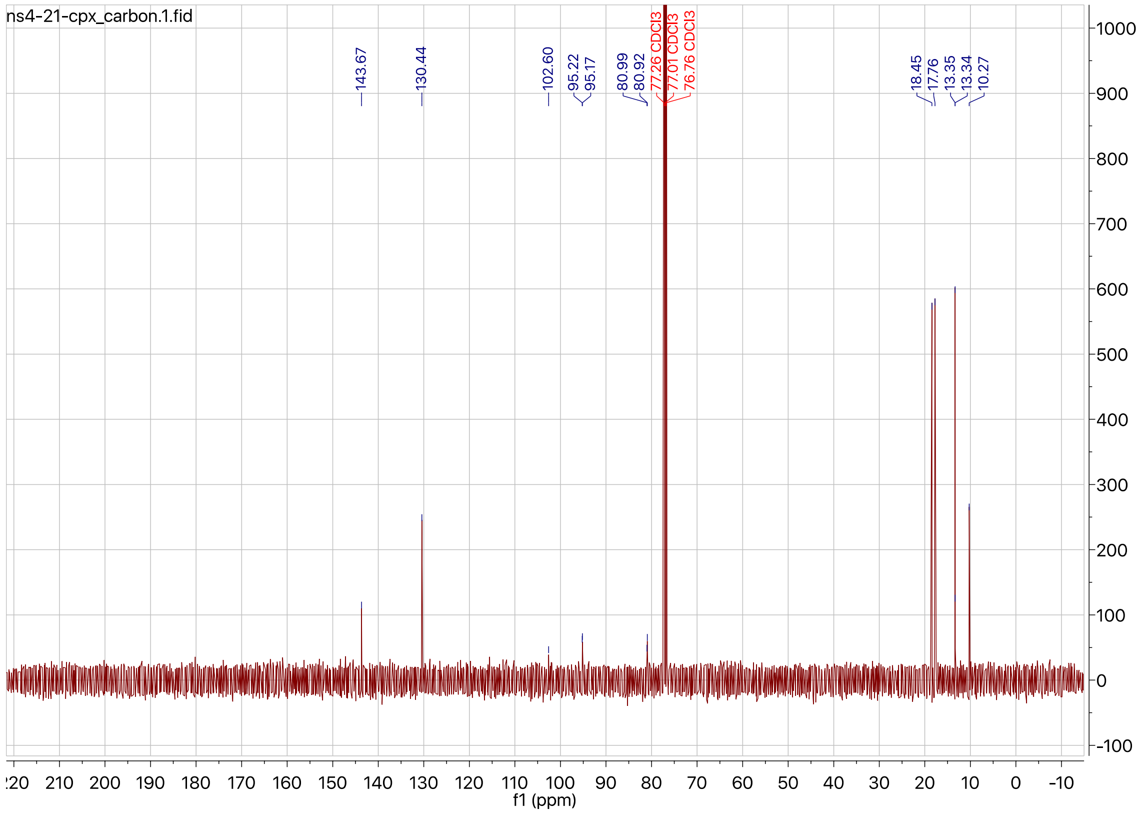

ns4-21-cpx_carbon.1.fid
143.67
130.44
102.60
95.22
95.17
80.99
80.92
77.26 CDCl3
77.01 CDCl3
76.76 CDCl3
18.45
17.76
13.35
13.34
10.27
f1 (ppm)

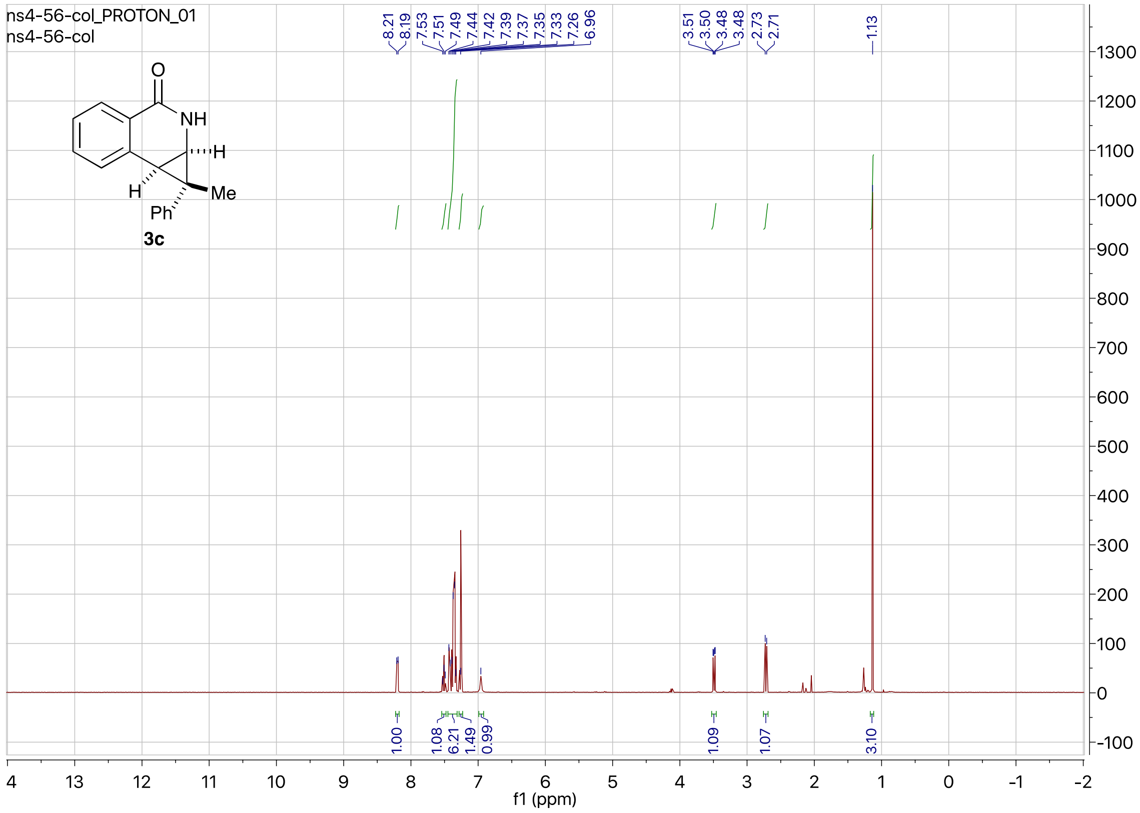
ns4-56-col_PROTON_01
ns4-56-col
3c
f1 (ppm)

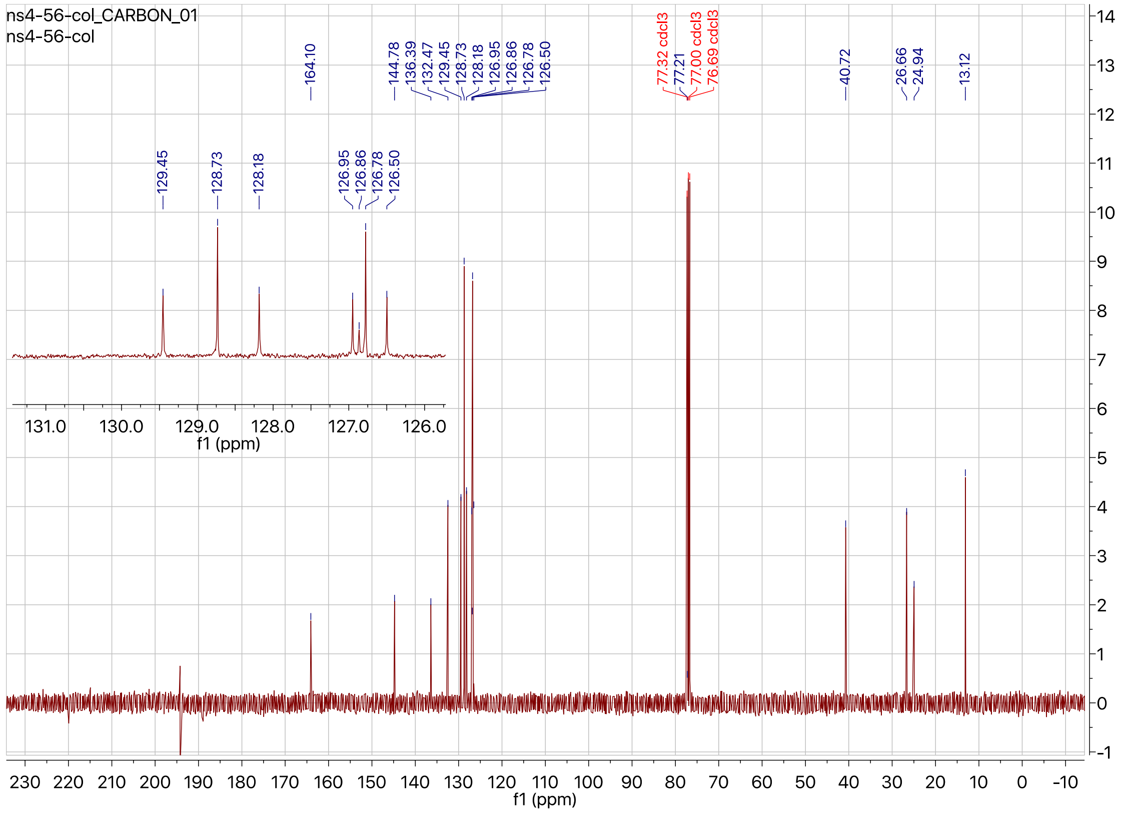
ns4-56-col_CARBON_01
ns4-56-col
f1 (ppm)

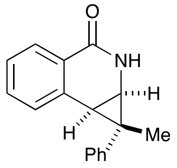

3c

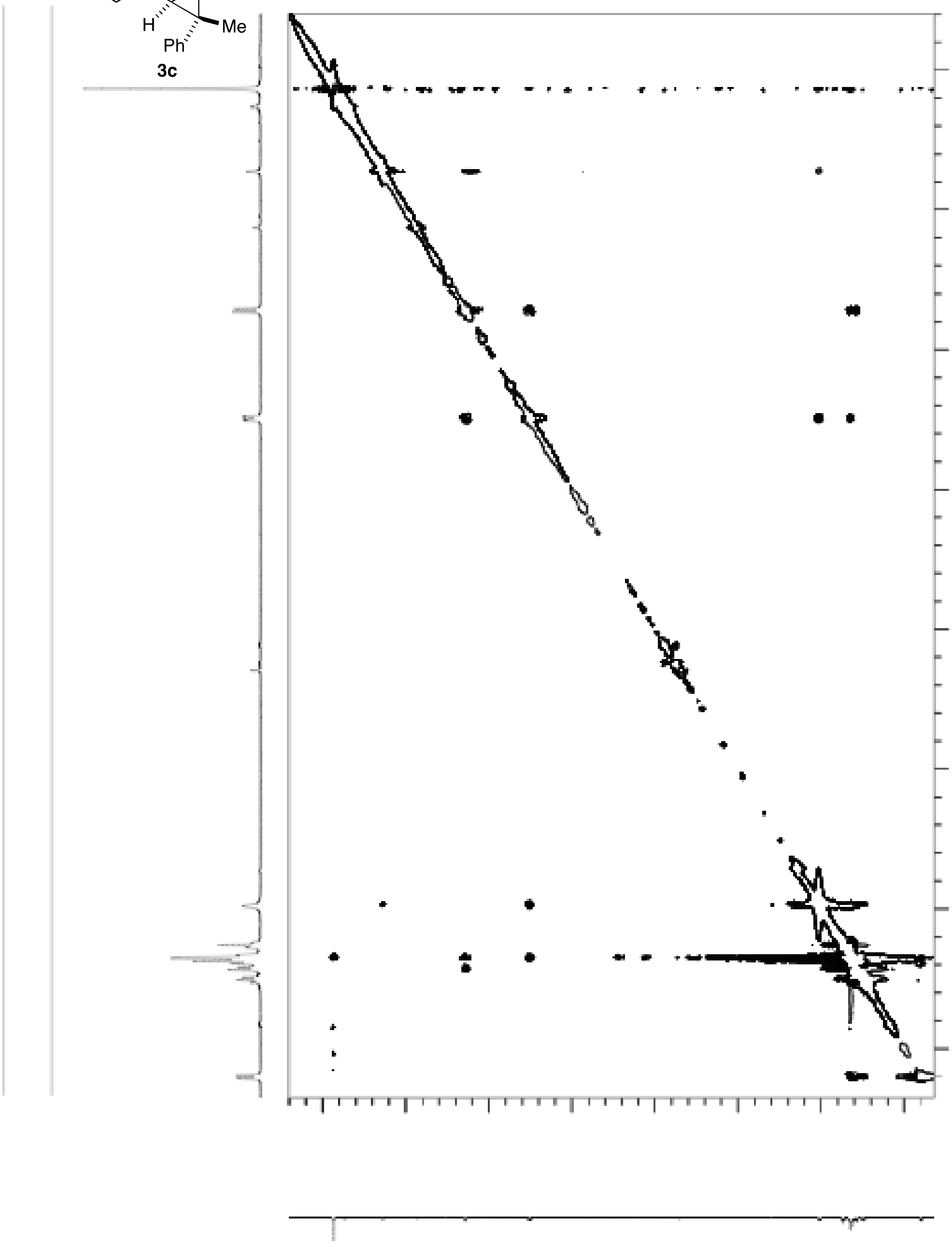

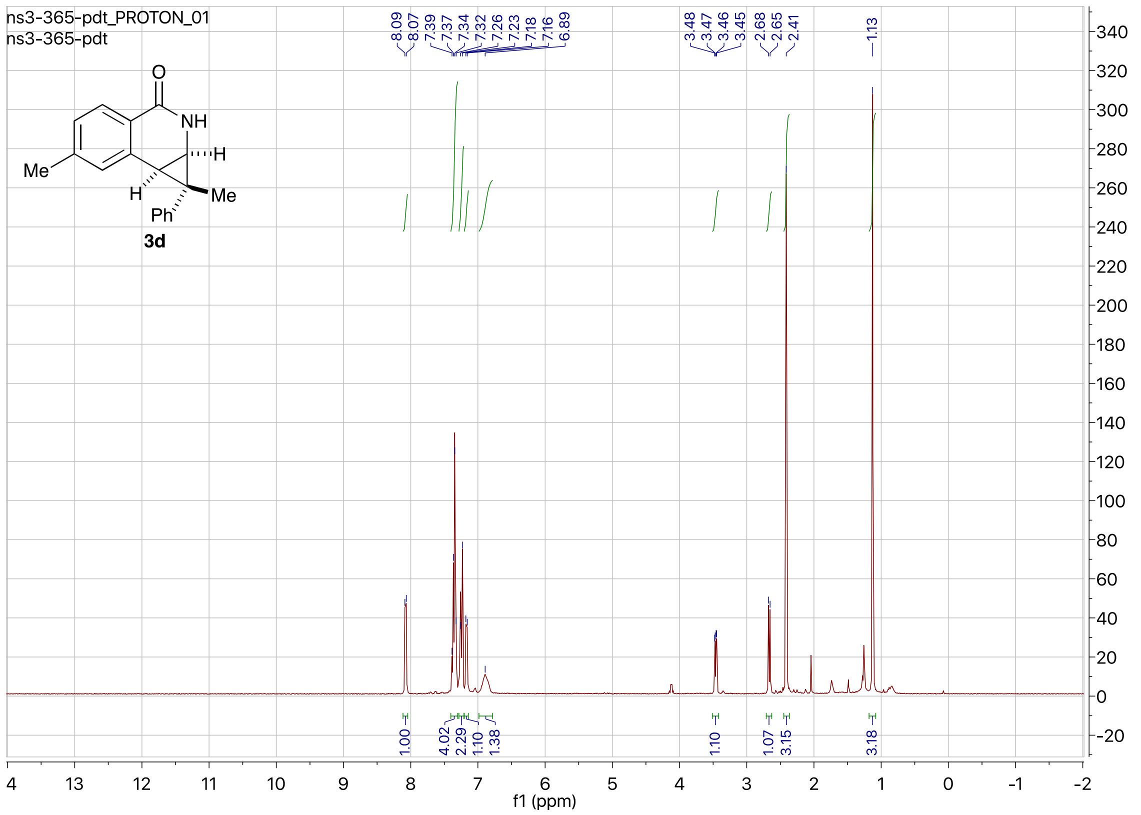
ns3-365-pdt_PROTON_01
ns3-365-pdt
O
NH
H
Me
H
Me
Ph
3d
8.09
8.07
7.39
7.37
7.34
7.32
7.26
7.23
7.18
7.16
6.89
3.48
3.47
3.46
3.45
2.68
2.65
2.41
1.13
1.00
4.02
2.29
1.10
1.38
1.10
1.07
3.15
3.18
f1 (ppm)

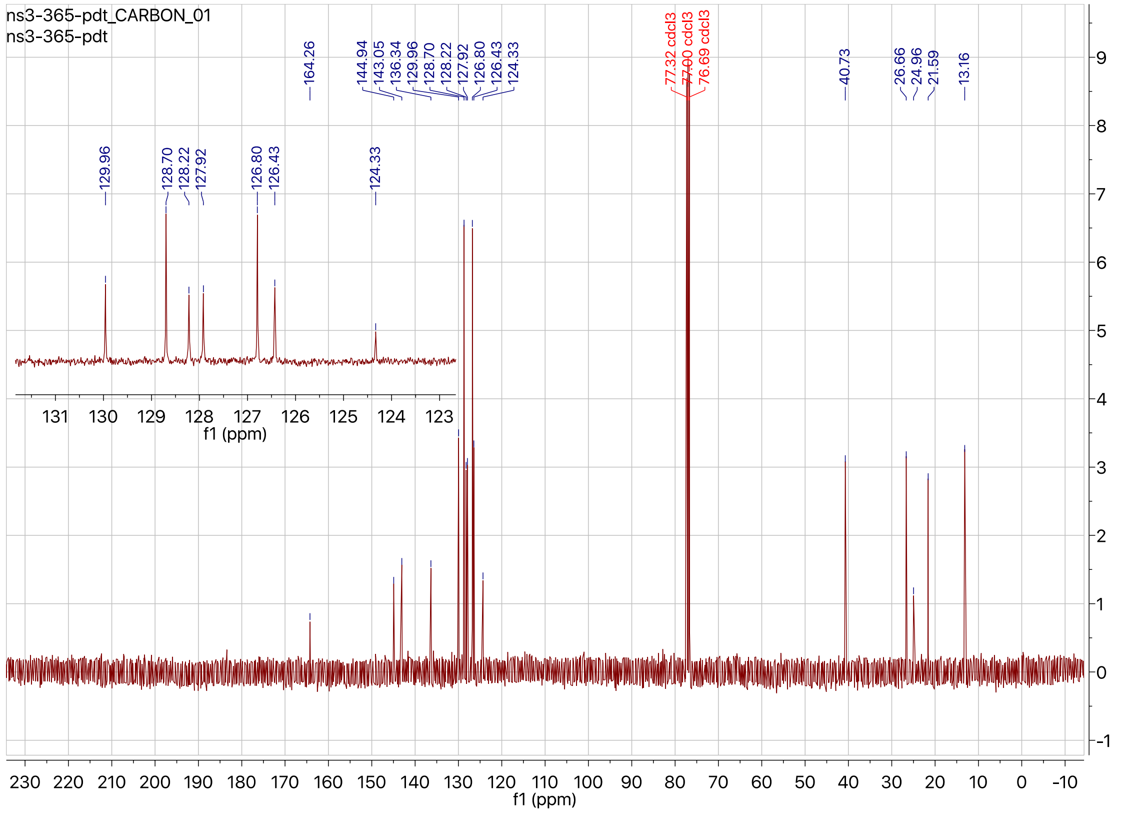
ns3-365-pdt_CARBON_01
ns3-365-pdt
164.26
144.94
143.05
136.34
129.96
128.70
128.22
127.92
126.80
126.43
124.33
77.32 cdcl3
77.00 cdcl3
76.69 cdcl3
40.73
26.66
24.96
21.59
13.16
f1 (ppm)

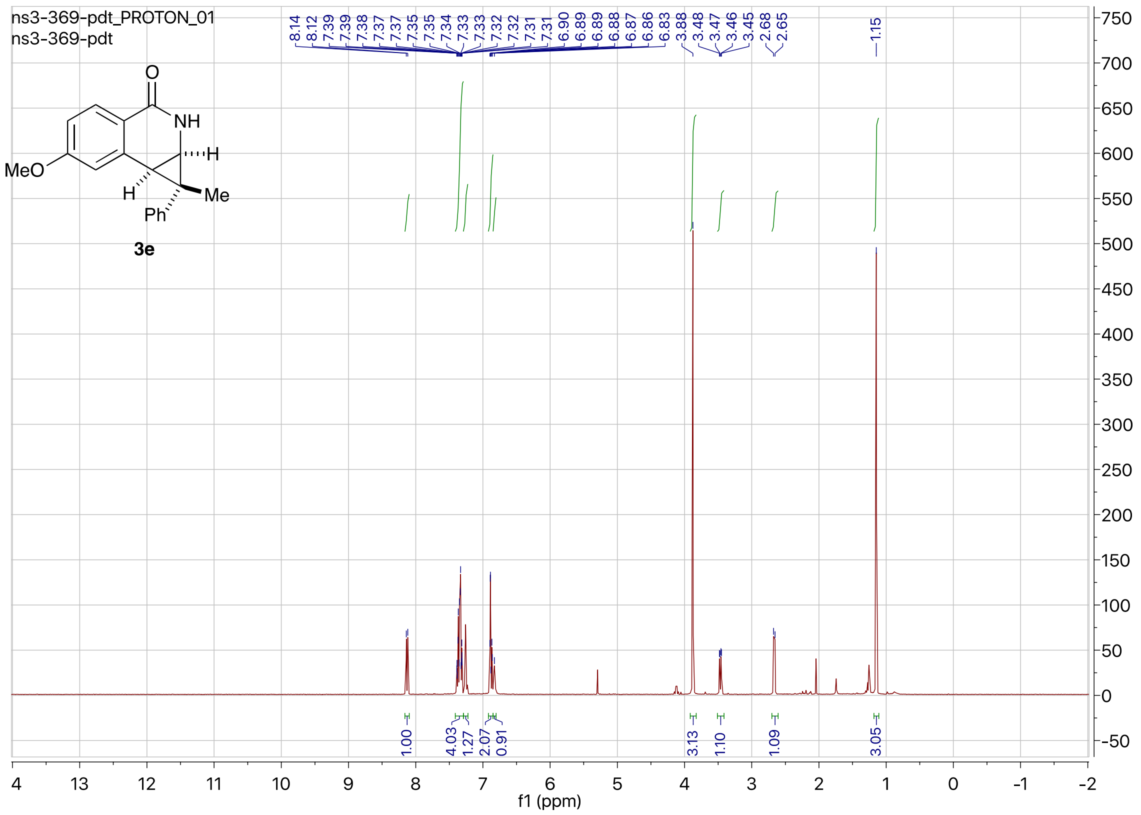
ns3-369-pdt_PROTON_01
ns3-369-pdt
O
NH
H
MeO
H
Me
Ph
3e
8.14 8.12 7.39 7.39 7.38 7.37 7.37 7.35 7.35 7.34 7.33 7.33 7.32 7.32 7.31 6.90 6.89 6.89 6.88 6.87 6.86 6.83 3.88 3.48 3.47 3.46 3.45 2.68 2.65
1.15
1.00 4.03 1.27 2.07 0.91 3.13 1.10 1.09 3.05
f1 (ppm)

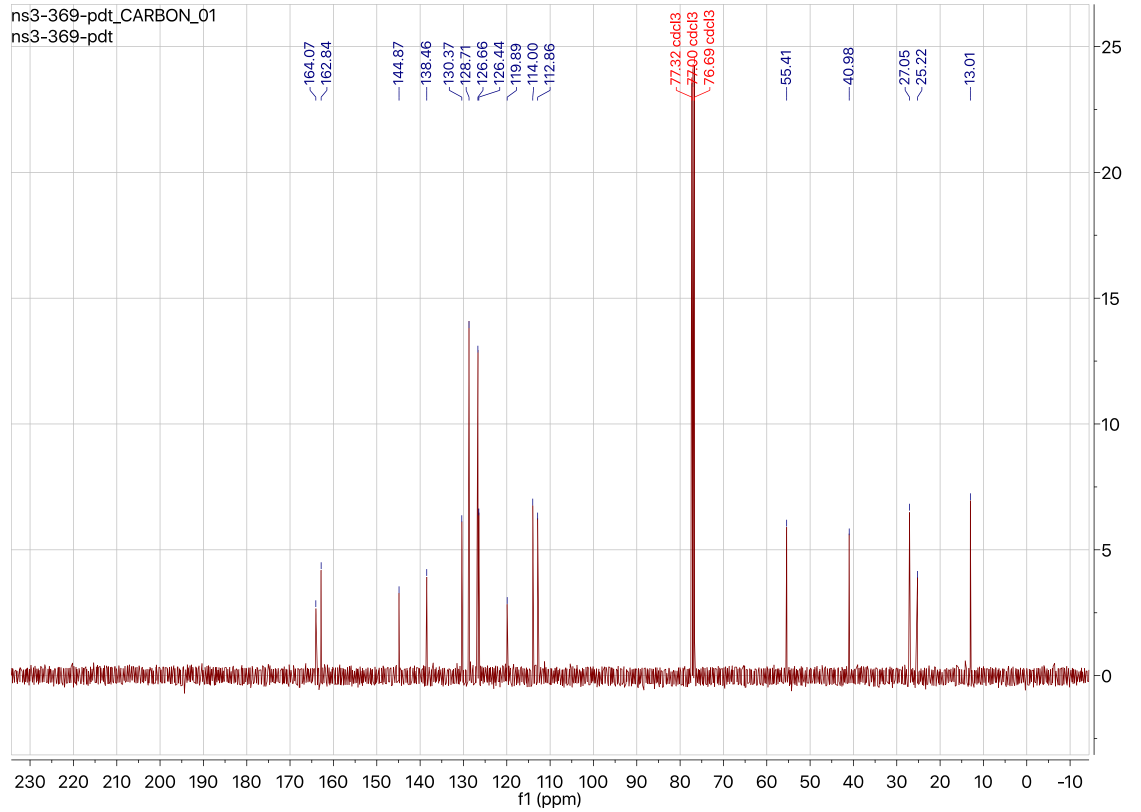
ns3-369-pdt_CARBON_01
ns3-369-pdt
164.07 162.84
144.87 138.46 130.37 128.71 126.66 126.44 119.89 114.00 112.86
77.32 cdcl3 77.00 cdcl3 76.69 cdcl3
55.41
40.98
27.05 25.22
13.01
f1 (ppm)

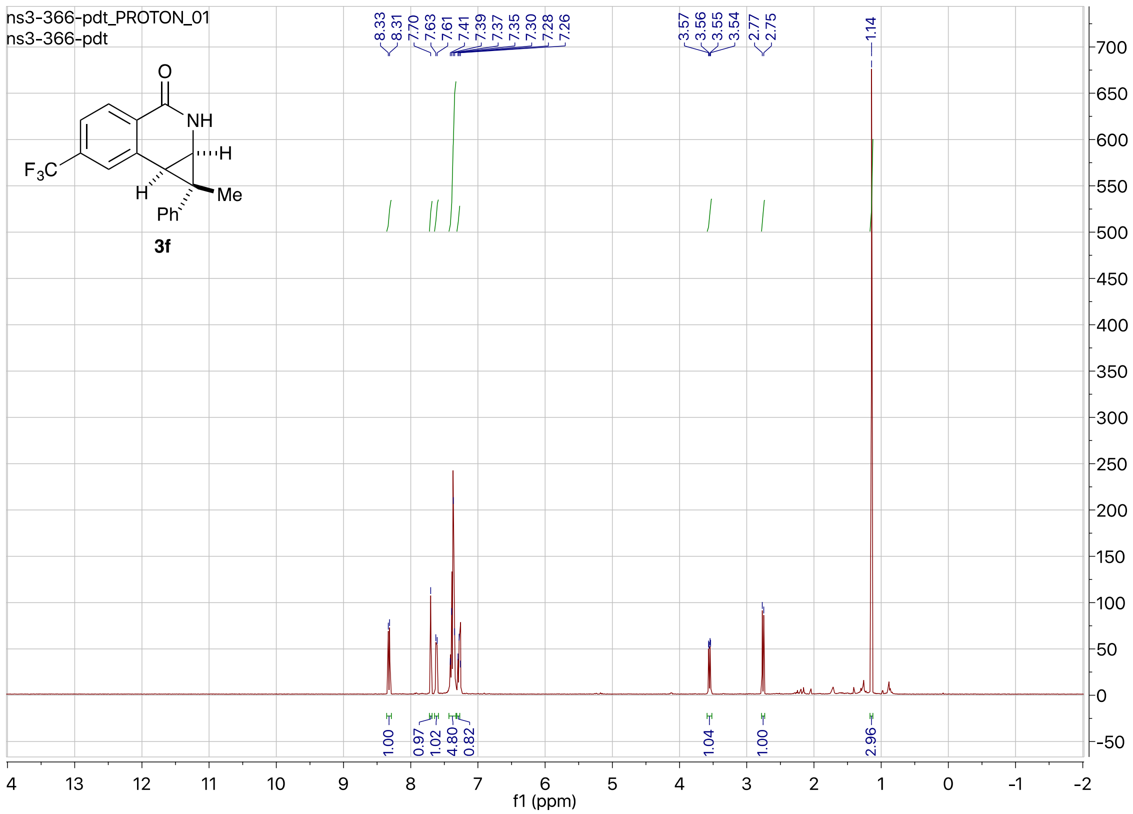
ns3-366-pdt_PROTON_01
ns3-366-pdt
O
NH
H
F_3C
H
Me
Ph
3f
8.33
8.31
7.70
7.63
7.61
7.41
7.39
7.37
7.35
7.30
7.28
7.26
3.57
3.56
3.55
3.54
2.77
2.75
1.14
1.00
0.97
1.02
4.80
0.82
1.04
1.00
2.96
f1 (ppm)

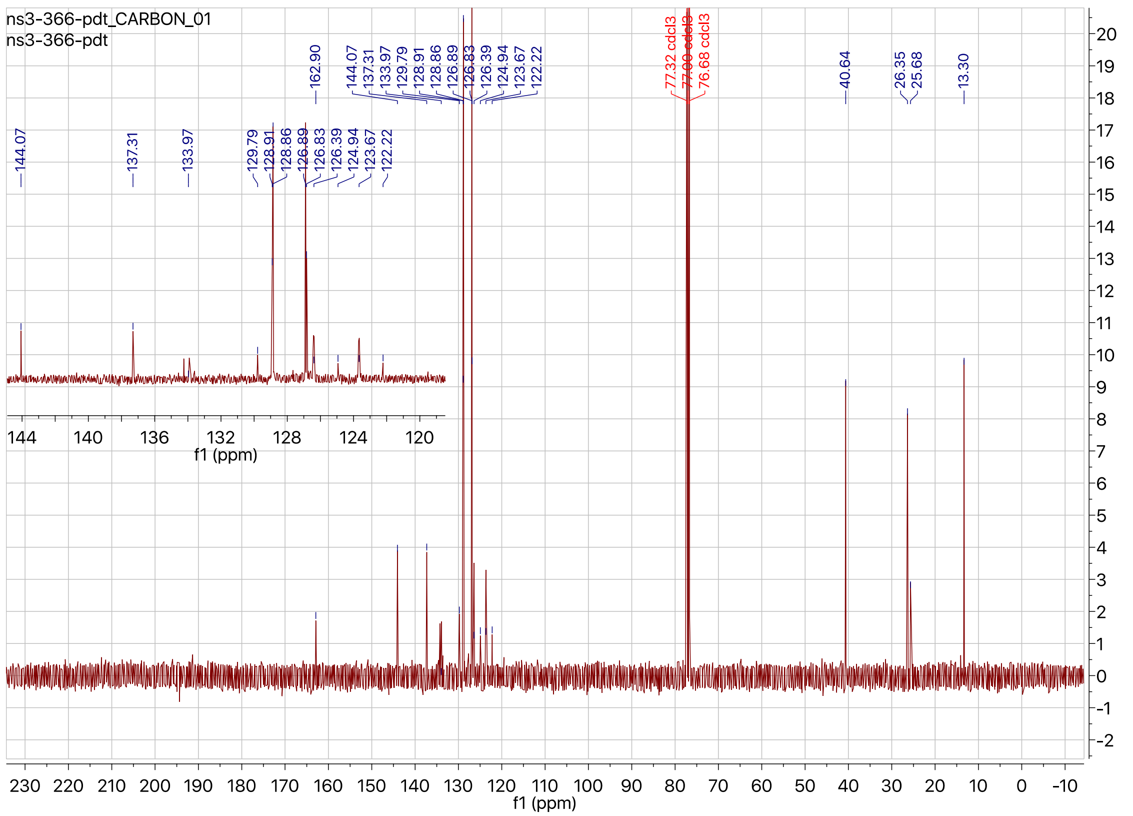
ns3-366-pdt_CARBON_01
ns3-366-pdt
162.90
144.07
137.31
133.97
129.79
128.91
128.86
126.89
126.83
126.39
124.94
123.67
122.22
77.32 cdcl3
77.00 cdcl3
76.68 cdcl3
40.64
26.35
25.68
13.30
f1 (ppm)

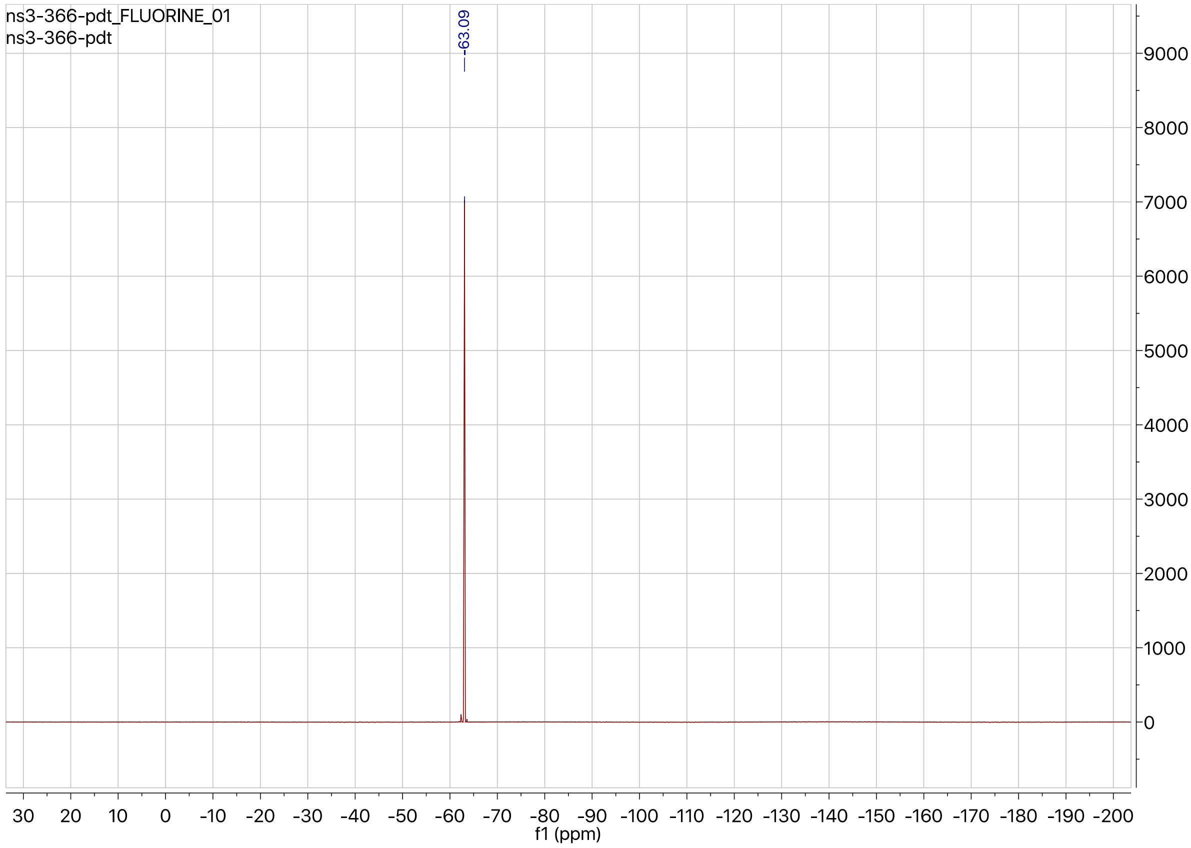
ns3-366-pdt_FLUORINE_01
ns3-366-pdt
-63.09
9000
8000
7000
6000
5000
4000
3000
2000
1000
0
30 20 10 0 -10 -20 -30 -40 -50 -60 -70 -80 -90 -100 -110 -120 -130 -140 -150 -160 -170 -180 -190 -200
f1 (ppm)

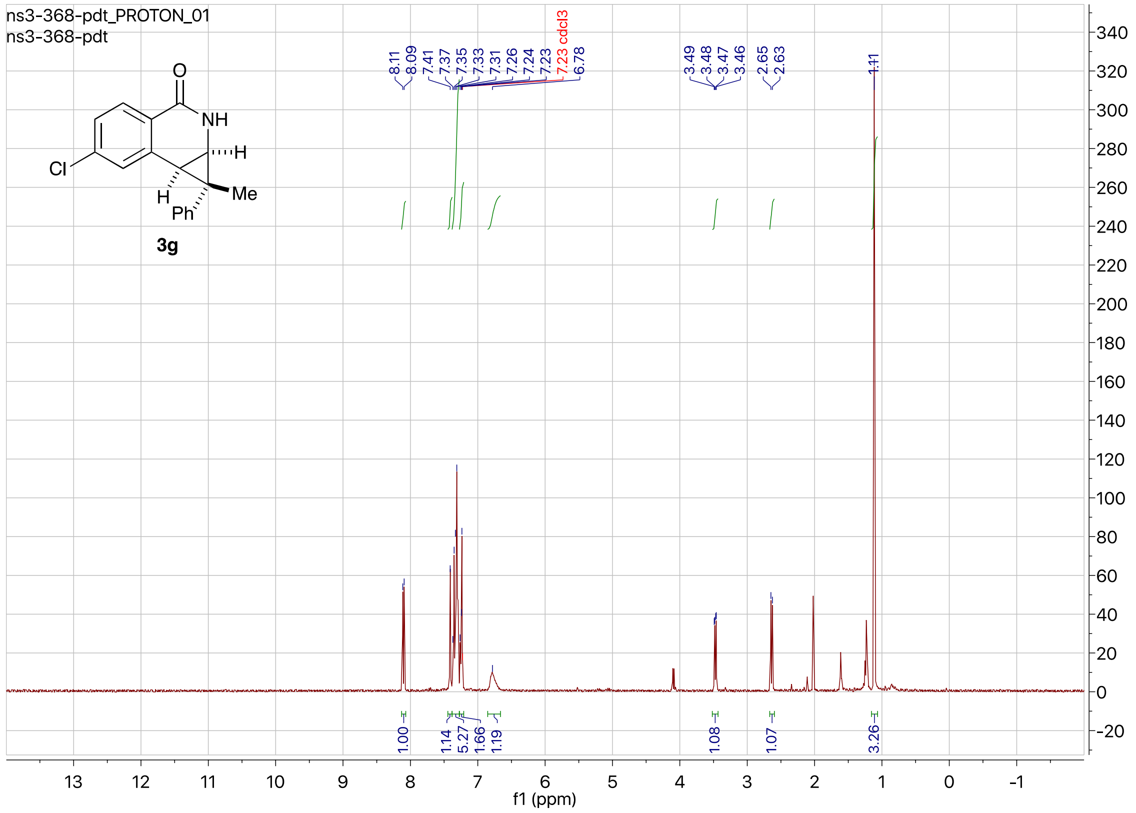
ns3-368-pdt_PROTON_01
ns3-368-pdt
3g
O
NH
H
Cl
H
Me
Ph
8.11
8.09
7.41
7.37
7.35
7.33
7.31
7.26
7.24
7.23
7.23 cdcl3
6.78
3.49
3.48
3.47
3.46
2.65
2.63
1.11
1.00
1.14
5.27
1.66
1.19
1.08
1.07
3.26
f1 (ppm)

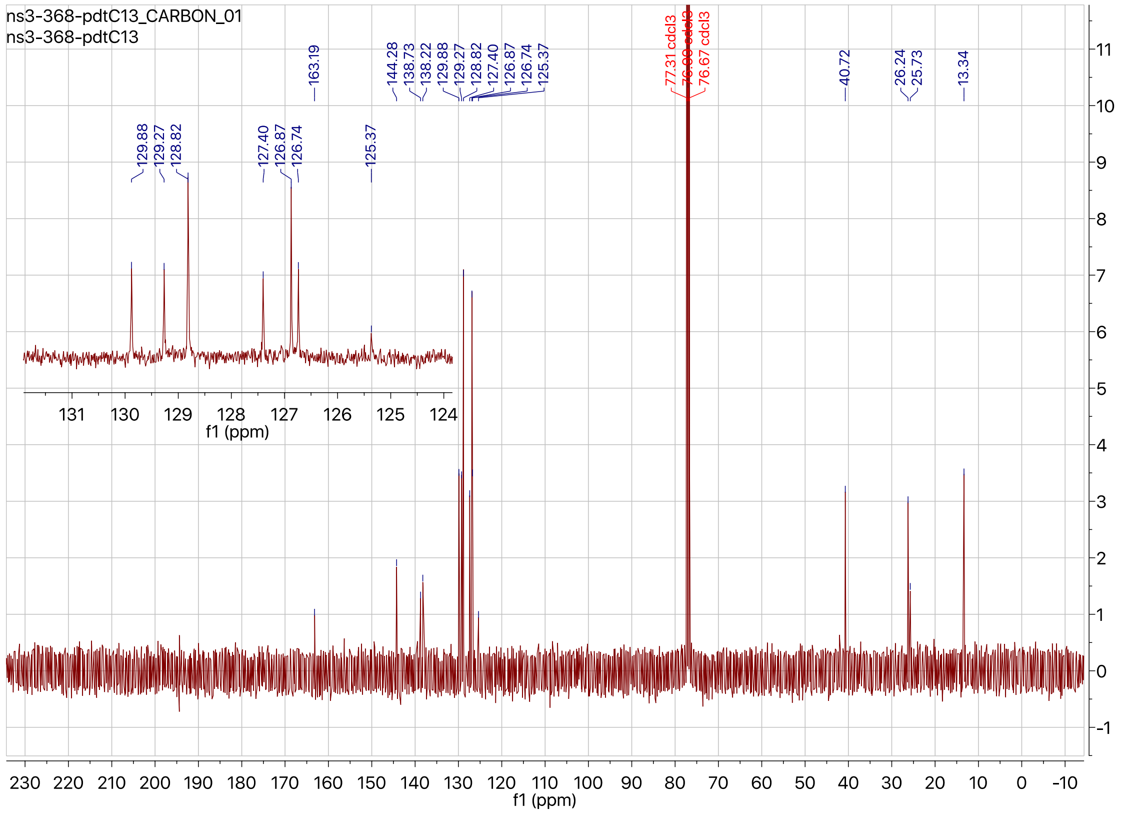
ns3-368-pdtC13_CARBON_01
ns3-368-pdtC13
163.19
144.28
138.73
138.22
129.88
129.27
128.82
127.40
126.87
126.74
125.37
77.31 cdcl3
76.67 cdcl3
40.72
26.24
25.73
13.34
f1 (ppm)

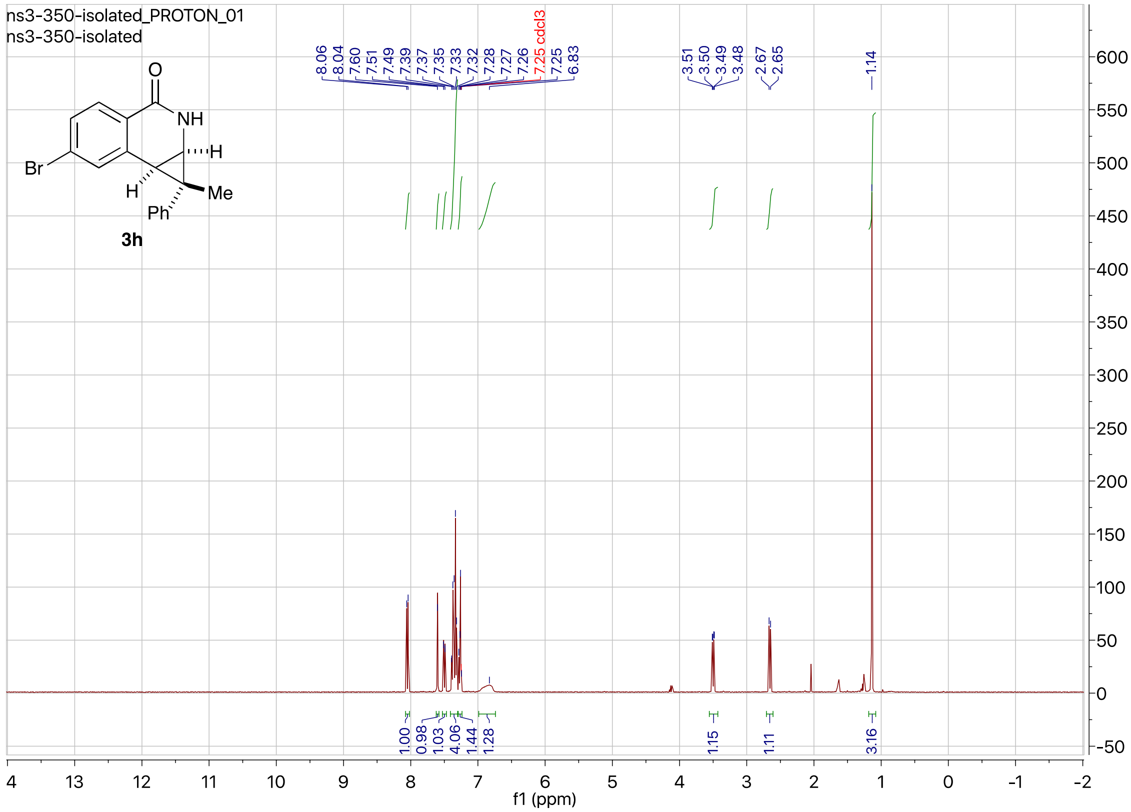
ns3-350-isolated_PROTON_01
ns3-350-isolated
8.06
8.04
7.60
7.51
7.49
7.39
7.37
7.35
7.33
7.32
7.28
7.27
7.26
7.25 cdcl3
7.25
6.83
3.51
3.50
3.49
3.48
2.67
2.65
1.14
O
NH
H
Br
H
Me
Ph
3h
1.00
0.98
1.03
4.06
1.44
1.28
1.15
1.11
3.16
f1 (ppm)

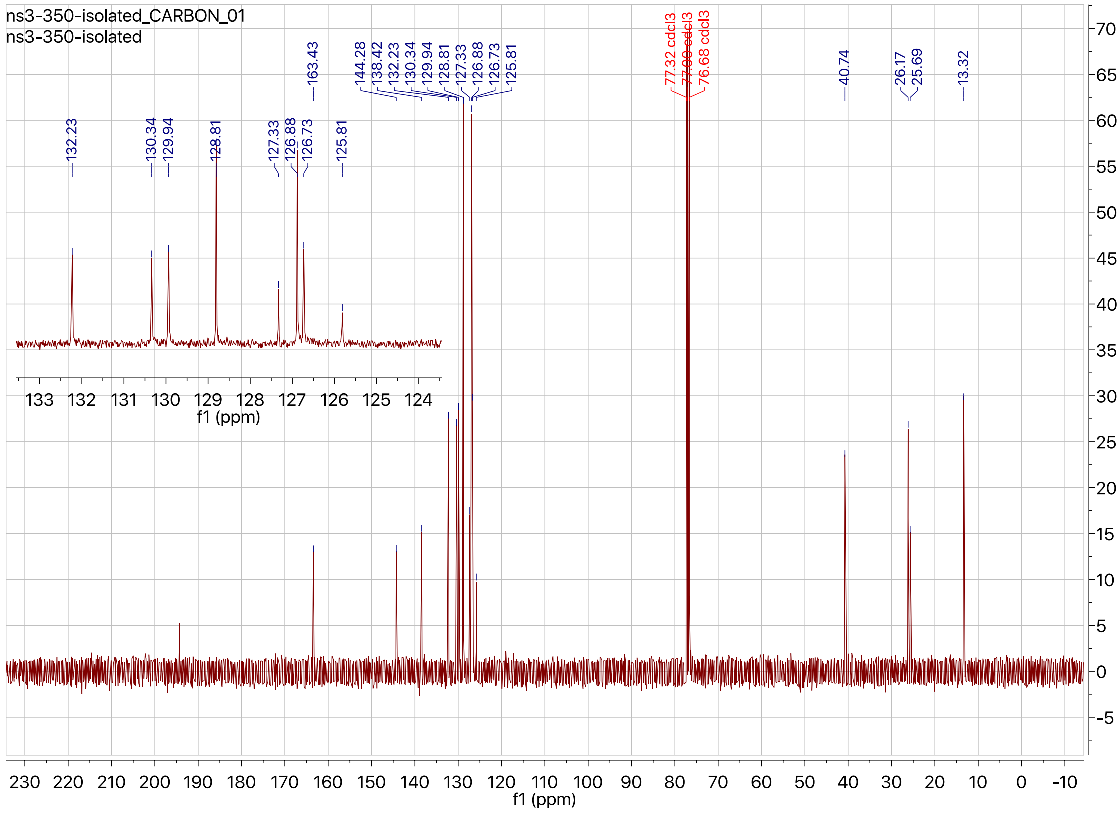
ns3-350-isolated_CARBON_01
ns3-350-isolated
163.43
144.28
138.42
132.23
130.34
129.94
128.81
127.33
126.88
126.73
125.81
77.32 cdcl3
77.00 cdcl3
76.68 cdcl3
40.74
26.17
25.69
13.32
f1 (ppm)

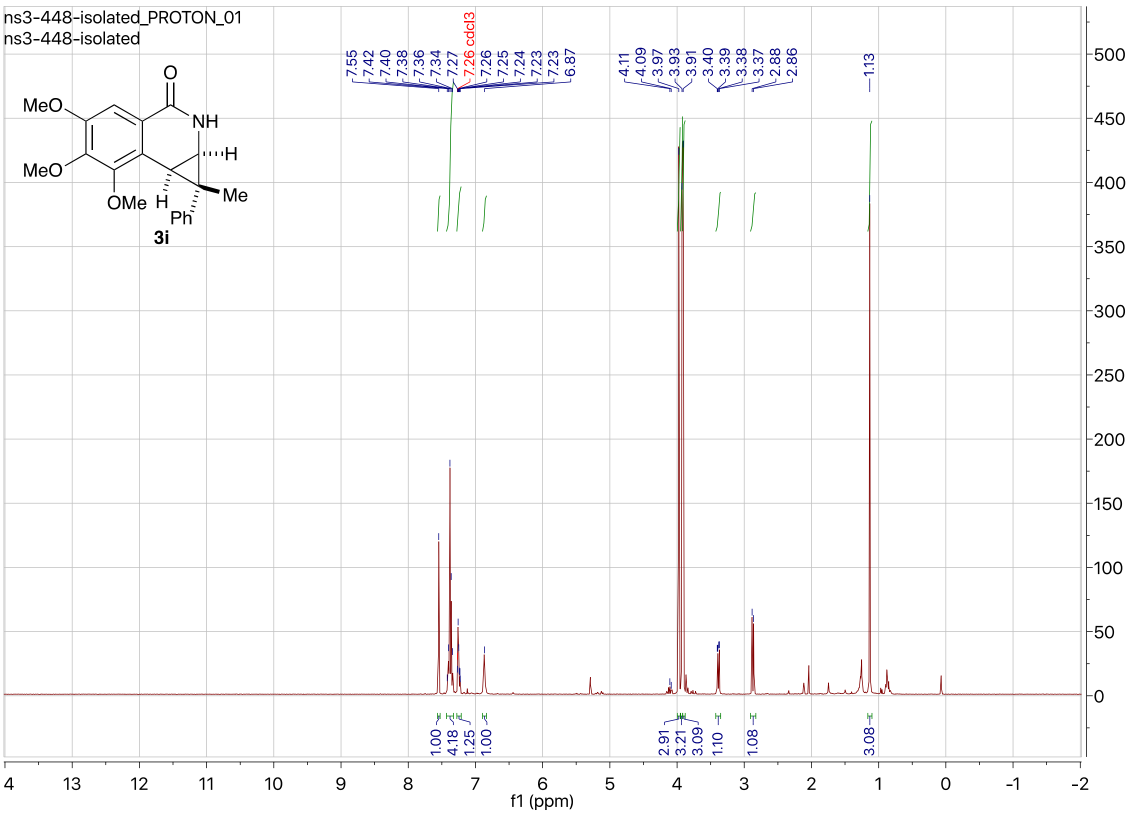

ns3-448-isolated_PROTON_01
ns3-448-isolated
7.55 7.42 7.40 7.38 7.36 7.34 7.27 7.26 cdcl3 7.26 7.25 7.24 7.23 7.23 6.87
4.11 4.09 3.97 3.93 3.91 3.40 3.39 3.38 3.37 2.88 2.86
1.13
MeO
MeO
OMe
O
NH
H
Me
H
Ph
3i
1.00 4.18 1.25 1.00 2.91 3.21 3.09 1.10 1.08 3.08
f1 (ppm)

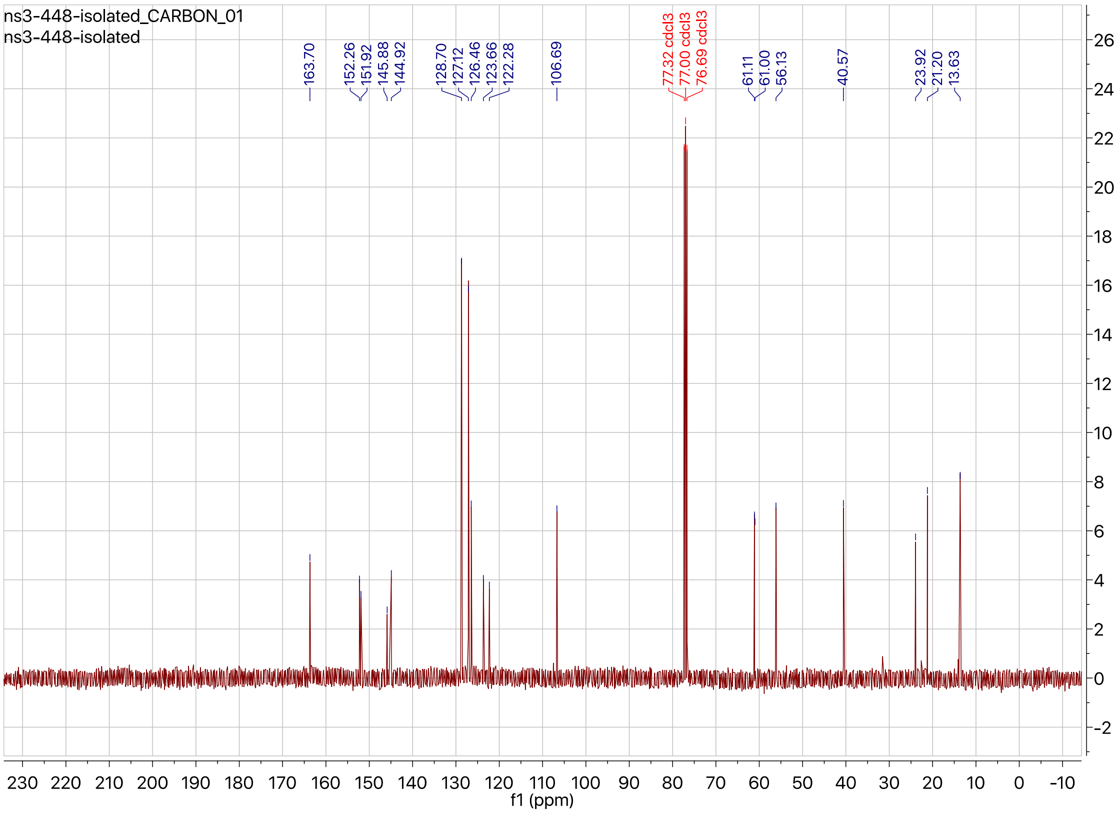

ns3-448-isolated_CARBON_01
ns3-448-isolated
163.70
152.26 151.92 145.88 144.92
128.70 127.12 126.46 123.66 122.28
106.69
77.32 cdcl3 77.00 cdcl3 76.69 cdcl3
61.11 61.00 56.13
40.57
23.92 21.20 13.63
f1 (ppm)

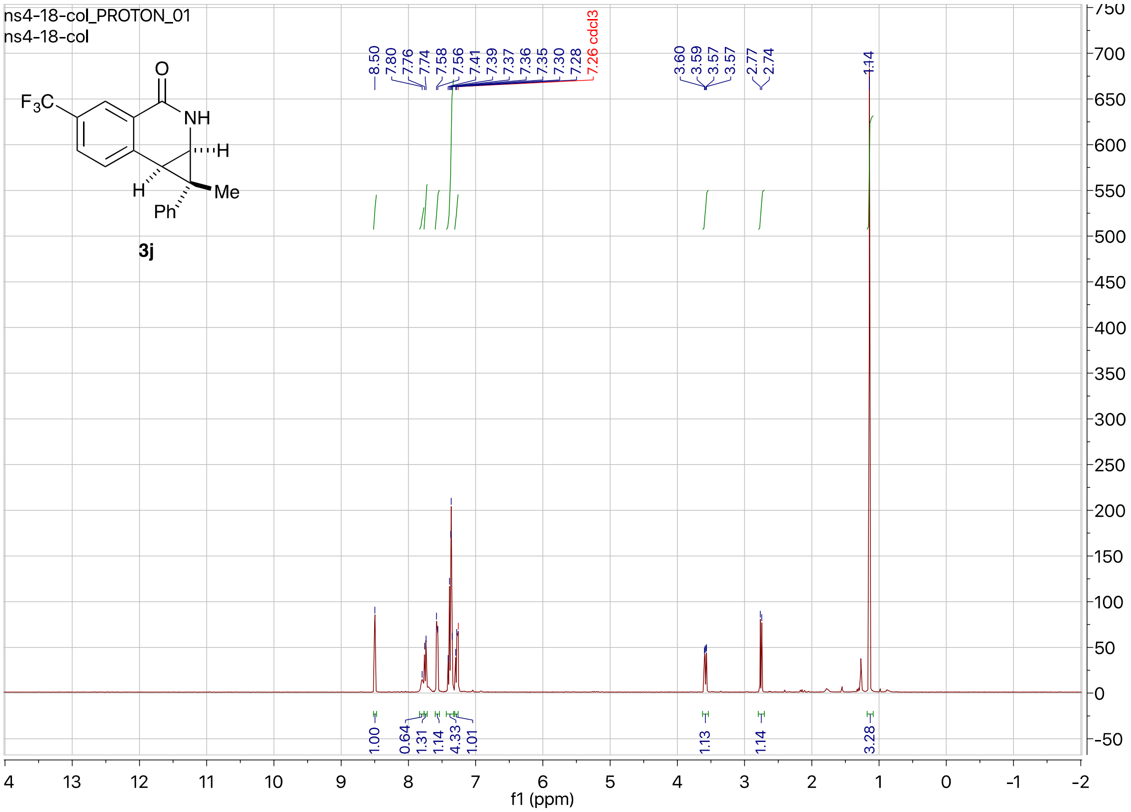
ns4-18-col_PROTON_01
ns4-18-col
3j
8.50
7.80
7.76
7.74
7.58
7.56
7.41
7.39
7.37
7.36
7.35
7.30
7.28
7.26 cdcl3
3.60
3.59
3.57
3.57
2.77
2.74
1.14
1.00
0.64
1.31
1.14
4.33
1.01
1.13
1.14
3.28
f1 (ppm)

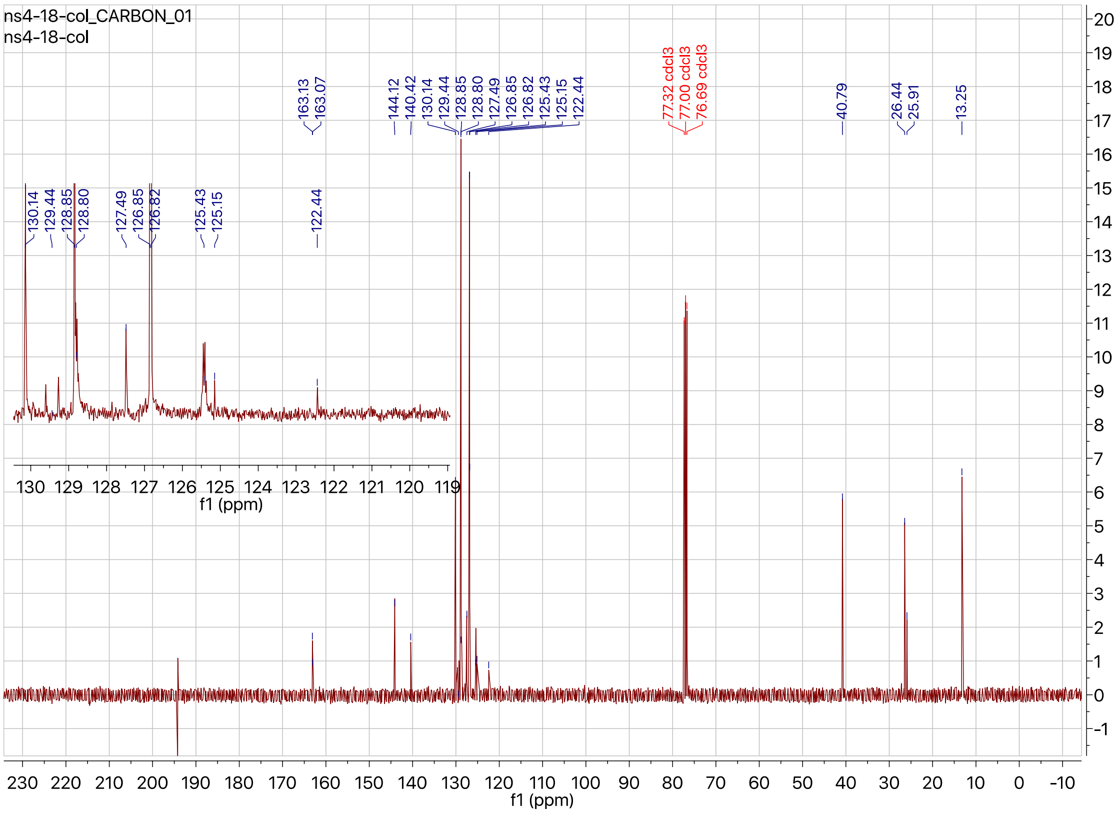
ns4-18-col_CARBON_01
ns4-18-col
163.13
163.07
144.12
140.42
130.14
129.44
128.85
128.80
127.49
126.85
126.82
125.43
125.15
122.44
77.32 cdcl3
77.00 cdcl3
76.69 cdcl3
40.79
26.44
25.91
13.25
f1 (ppm)

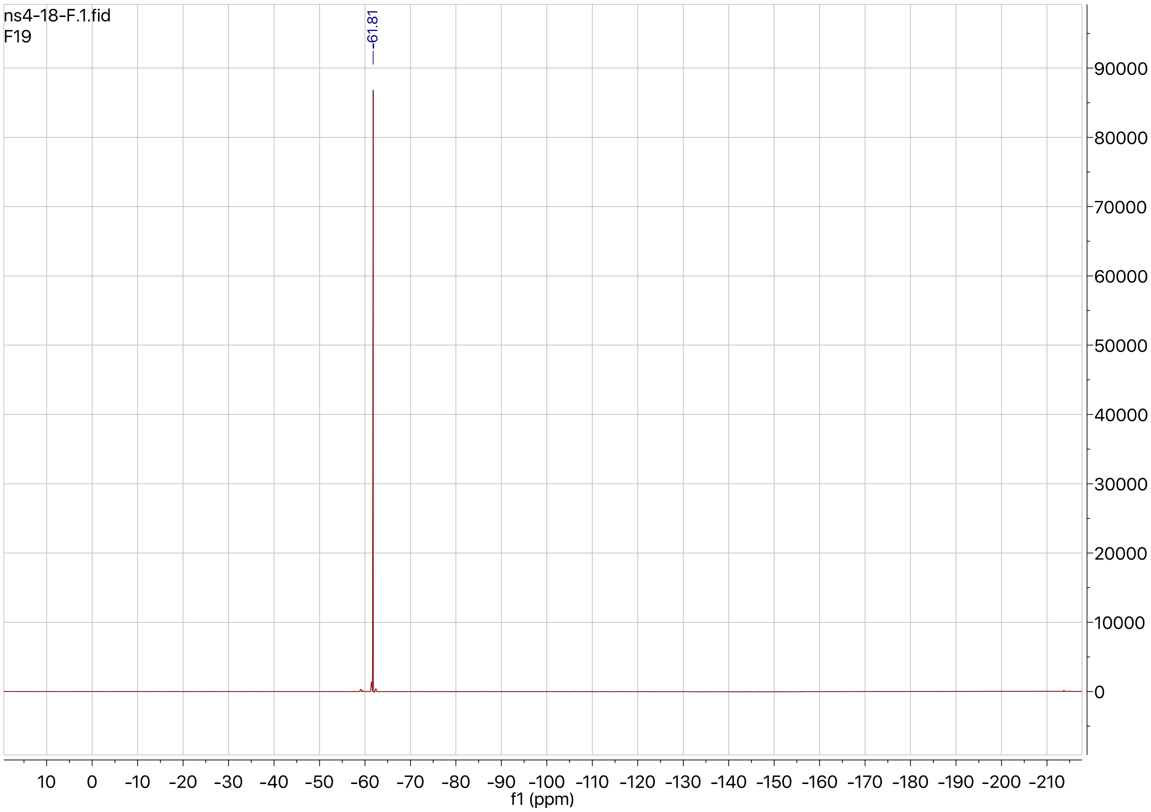
ns4-18-F.1.fid
F19
-61.81
90000
80000
70000
60000
50000
40000
30000
20000
10000
0
10 0 -10 -20 -30 -40 -50 -60 -70 -80 -90 -100 -110 -120 -130 -140 -150 -160 -170 -180 -190 -200 -210
f1 (ppm)

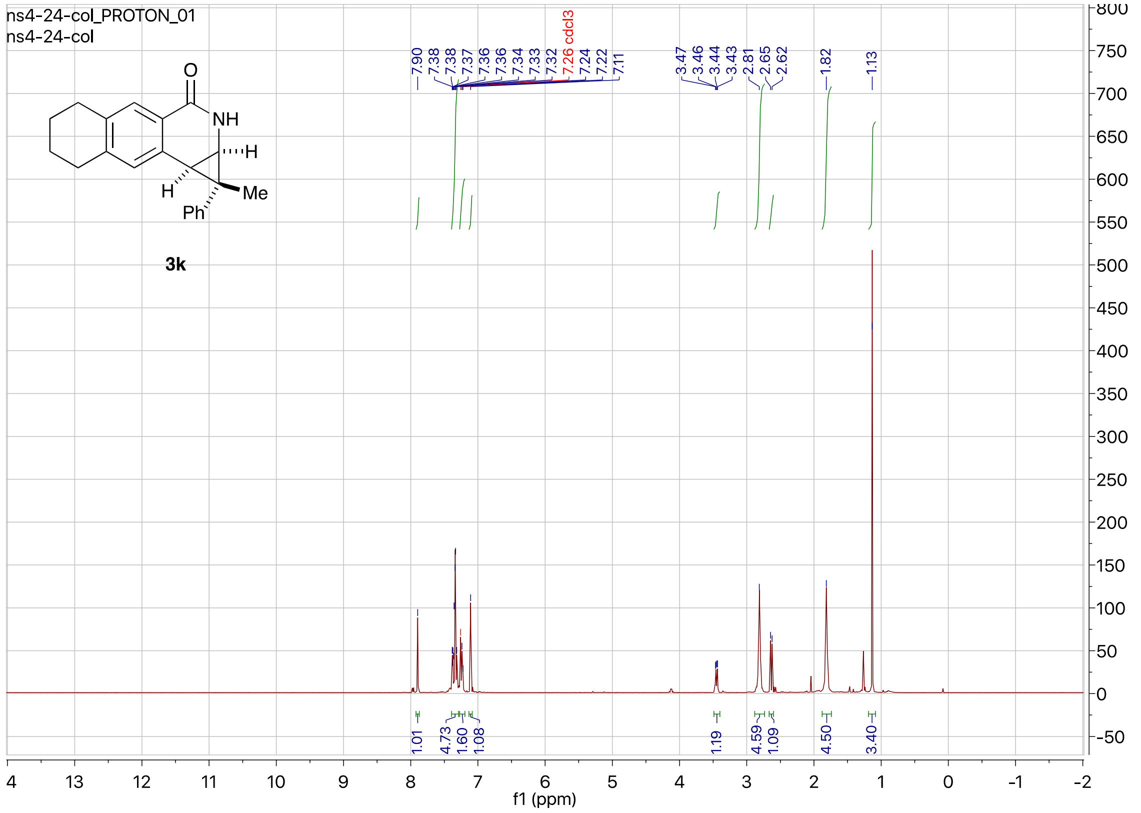

ns4-24-col_PROTON_01
ns4-24-col
3k
f1 (ppm)

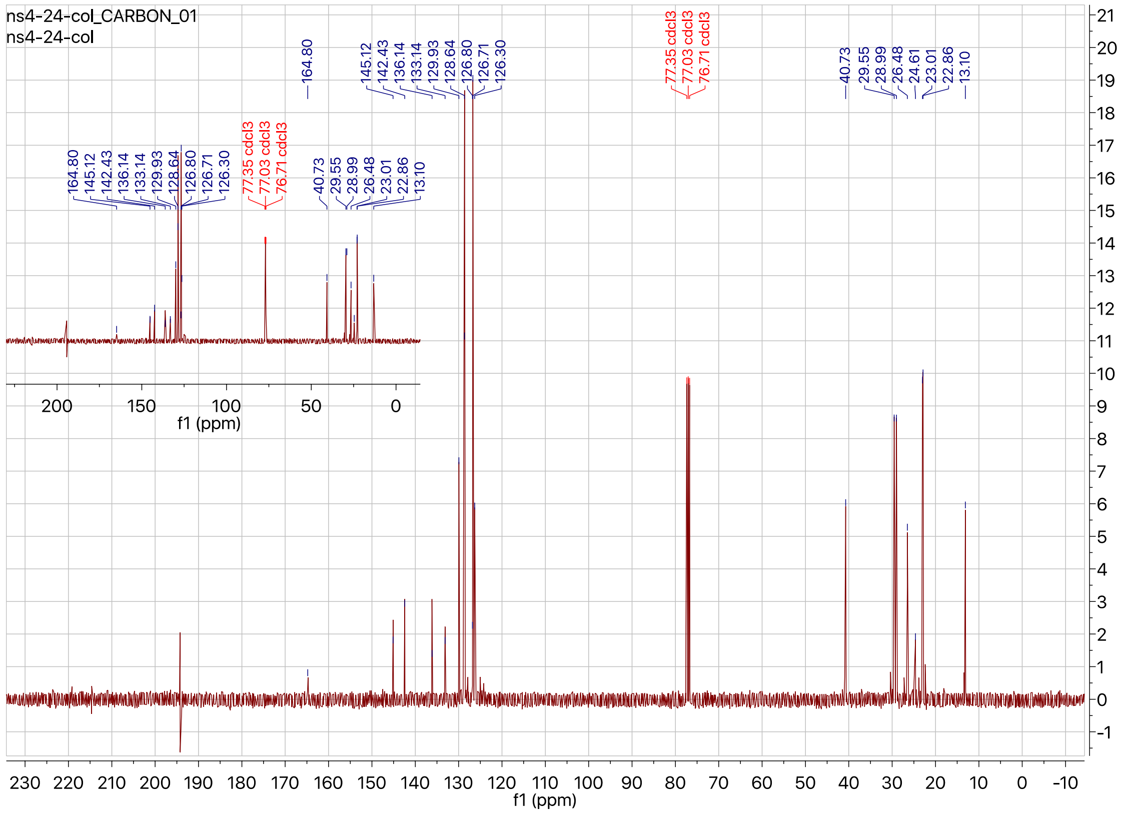

ns4-24-col_CARBON_01
ns4-24-col
f1 (ppm)

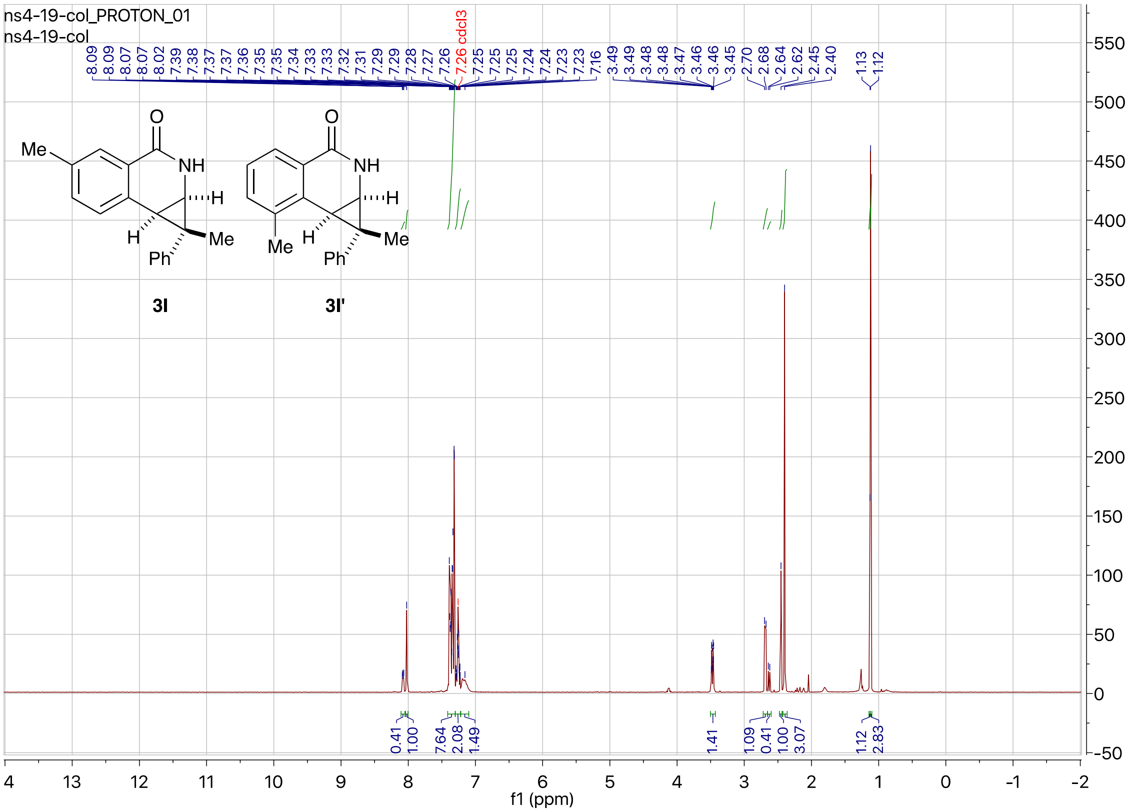
ns4-19-col_PROTON_01
ns4-19-col
8.09 8.09 8.07 8.02 7.39 7.38 7.37 7.37 7.36 7.35 7.35 7.34 7.33 7.33 7.32 7.31 7.29 7.29 7.28 7.27 7.26 7.26 cdcl3 7.25 7.25 7.25 7.24 7.24 7.23 7.23 7.16 3.49 3.49 3.48 3.48 3.47 3.46 3.46 3.45 2.70 2.68 2.64 2.62 2.45 2.40 1.13 1.12
O
NH
H
Me
Ph
3l
3l'
0.41 1.00 7.64 2.08 1.49 1.41 1.09 0.41 1.00 3.07 1.12 2.83
f1 (ppm)

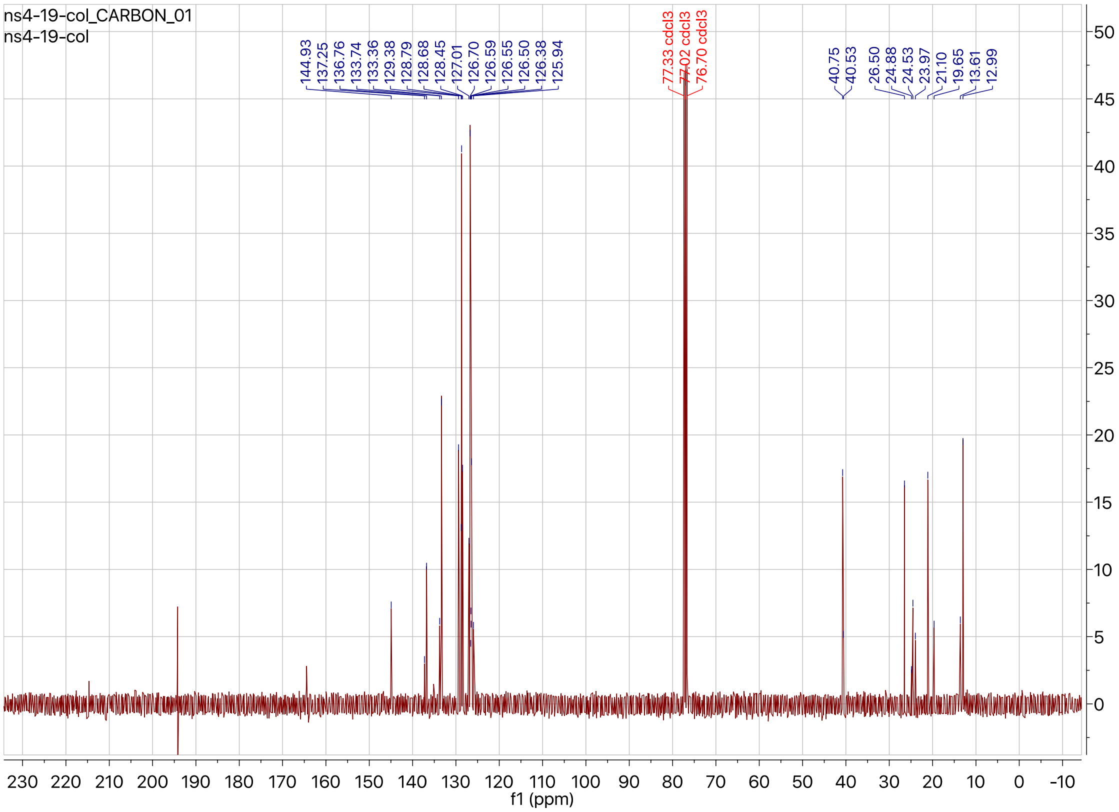
ns4-19-col_CARBON_01
ns4-19-col
144.93 137.25 136.76 133.74 133.36 129.38 128.79 128.68 128.45 127.01 126.70 126.59 126.55 126.50 126.38 125.94
77.33 cdcl3 77.02 cdcl3 76.70 cdcl3
40.75 40.53 26.50 24.88 24.53 23.97 21.10 19.65 13.61 12.99
f1 (ppm)

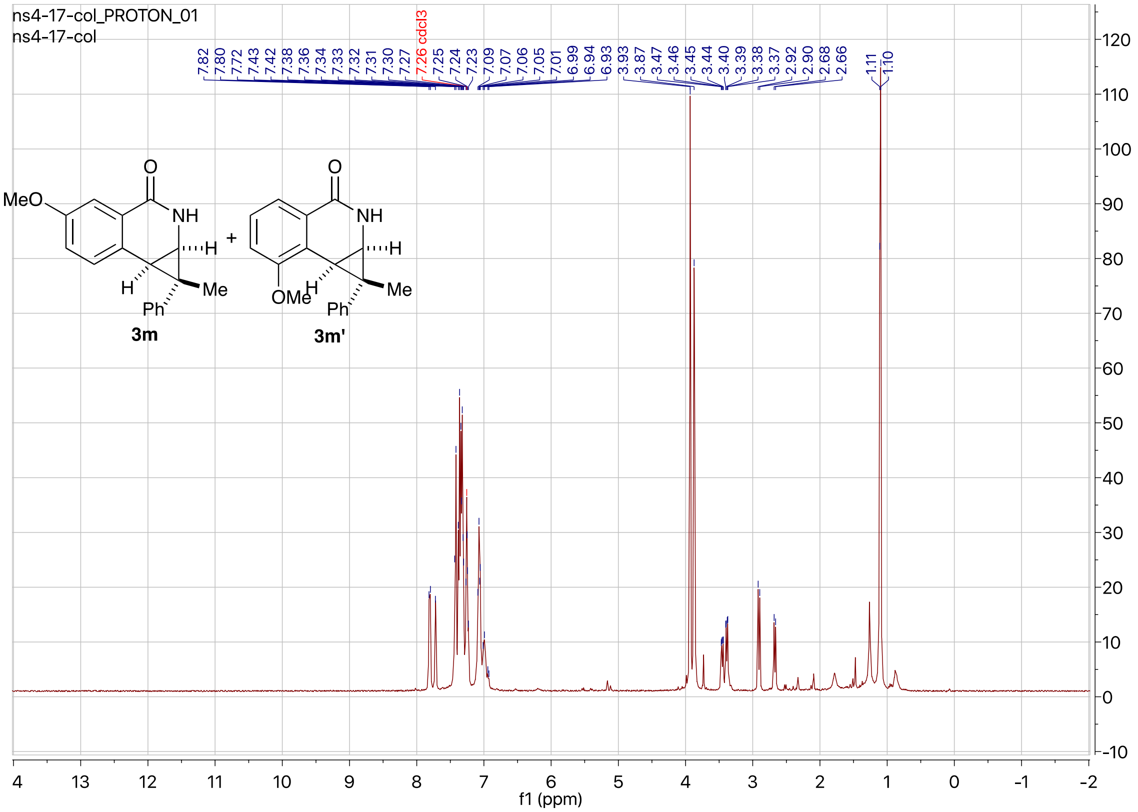
ns4-17-col_PROTON_01
ns4-17-col
7.82 7.80 7.72 7.43 7.42 7.38 7.36 7.34 7.33 7.32 7.31 7.30 7.27 7.26 cdcl3 7.25 7.24 7.23 7.09 7.07 7.06 7.05 7.01 6.99 6.94 6.93 3.93 3.87 3.47 3.46 3.45 3.44 3.40 3.39 3.38 3.37 2.92 2.90 2.68 2.66 1.11 1.10
MeO
O
NH
H
Me
Ph
3m
+
OMe
3m'
f1 (ppm)

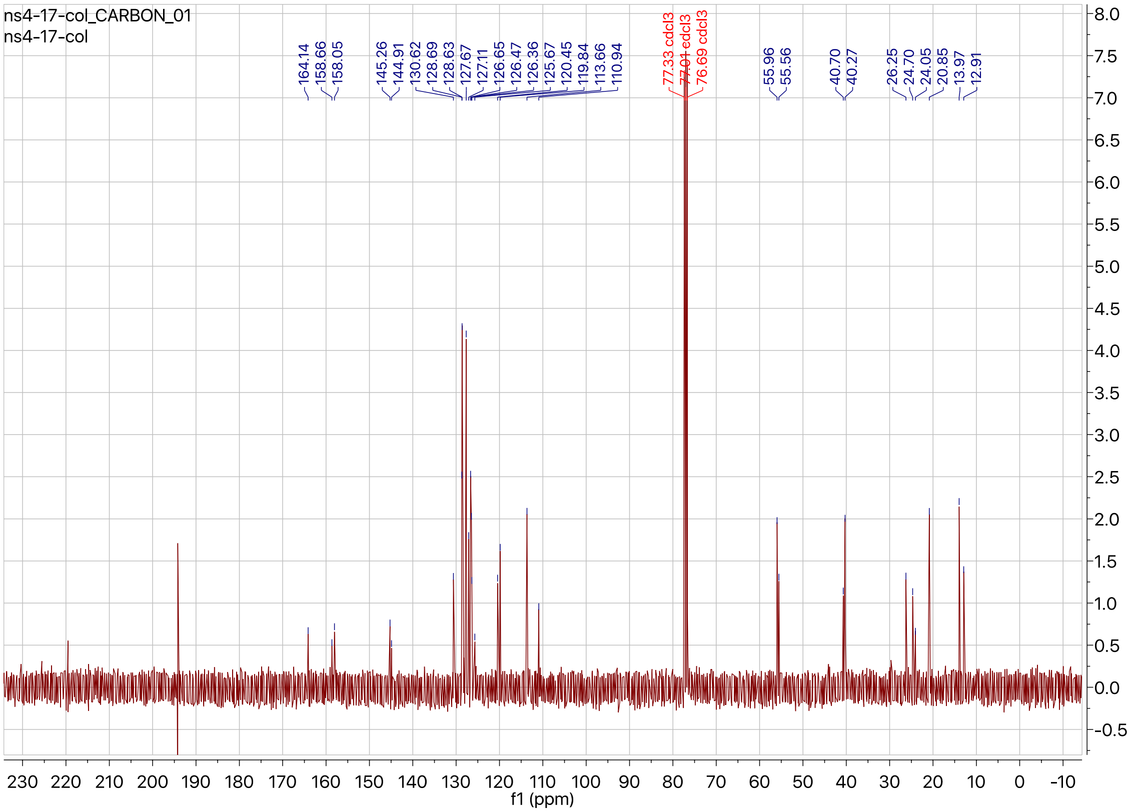
ns4-17-col_CARBON_01
ns4-17-col
164.14 158.66 158.05 145.26 144.91 130.62 128.69 128.63 127.67 127.11 126.65 126.47 126.36 125.67 120.45 119.84 113.66 110.94
77.33 cdcl3 77.01 cdcl3 76.69 cdcl3
55.96 55.56 40.70 40.27 26.25 24.70 24.05 20.85 13.97 12.91
f1 (ppm)

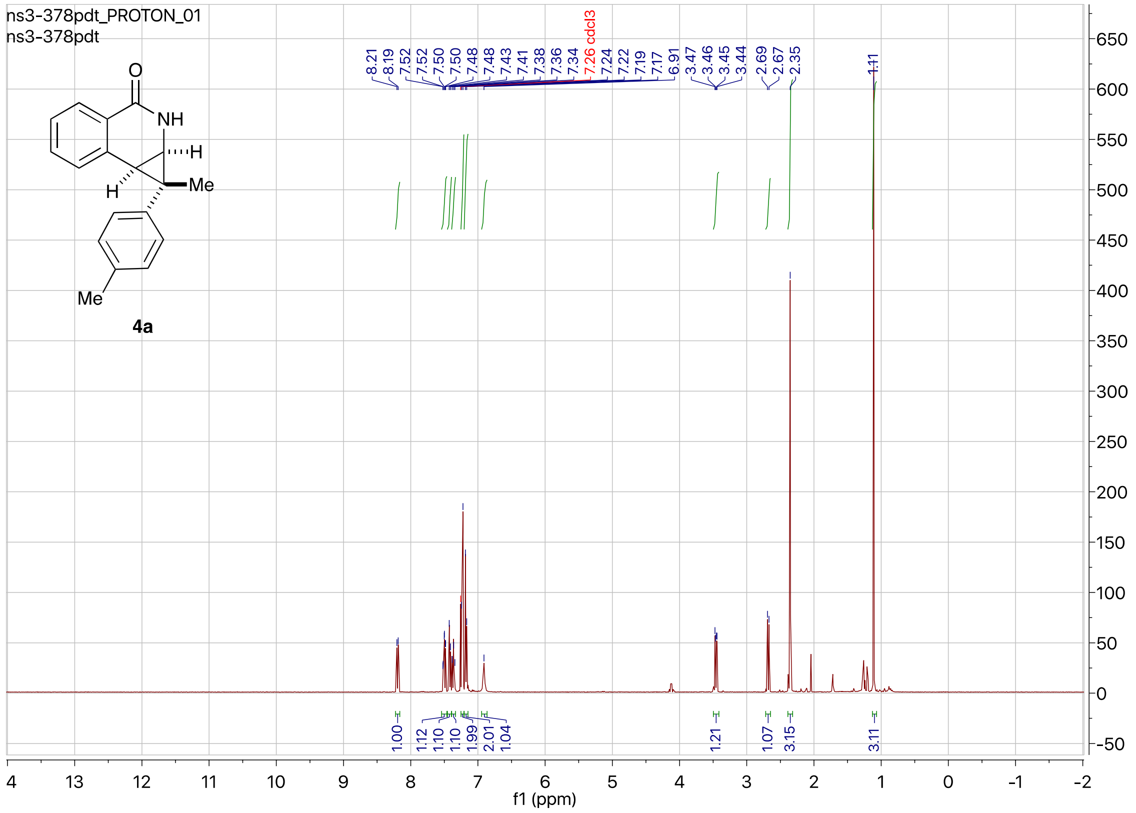

ns3-378pdt_PROTON_01
ns3-378pdt
4a

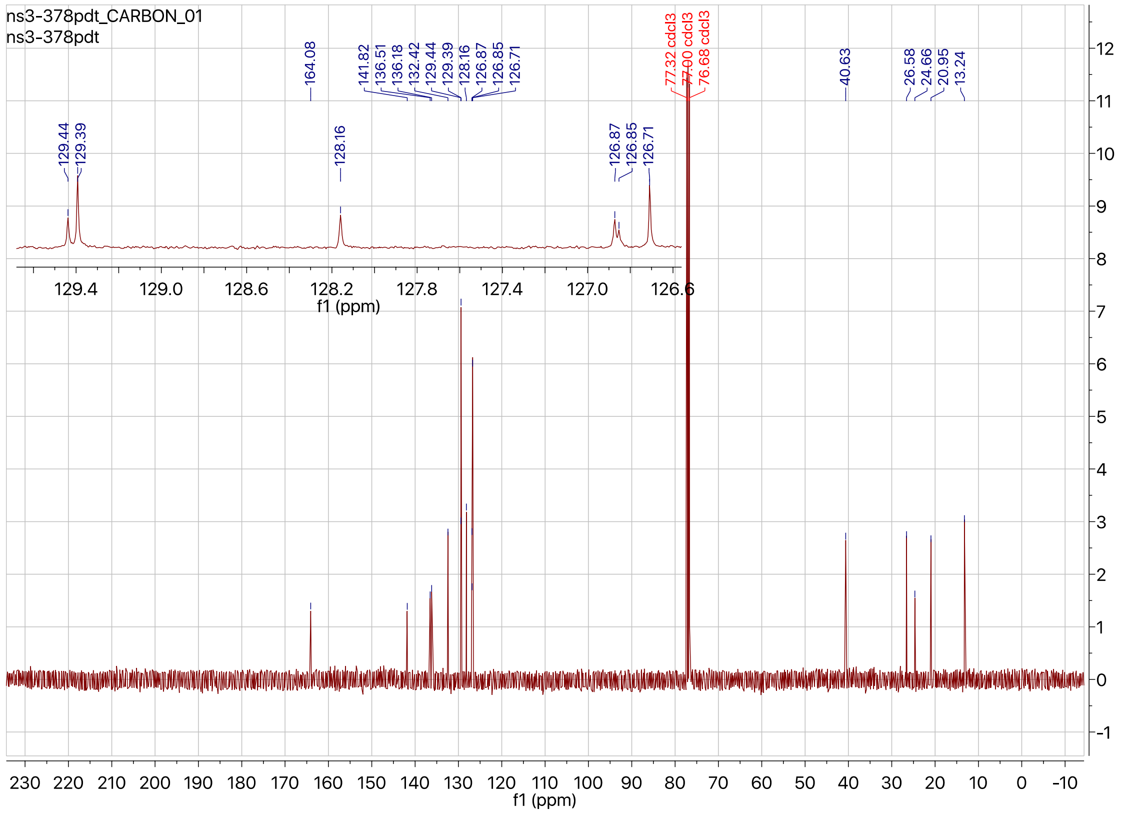

ns3-378pdt_CARBON_01
ns3-378pdt

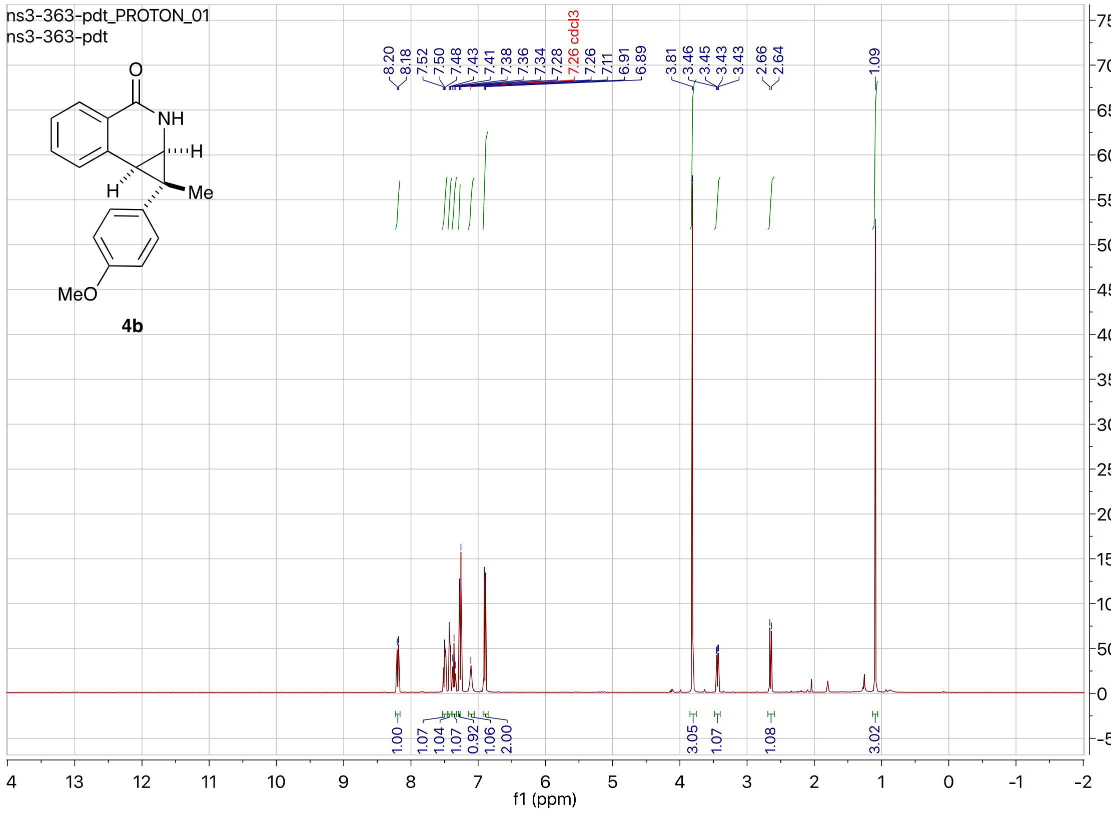
ns3-363-pdt_PROTON_01
ns3-363-pdt
4b
f1 (ppm)

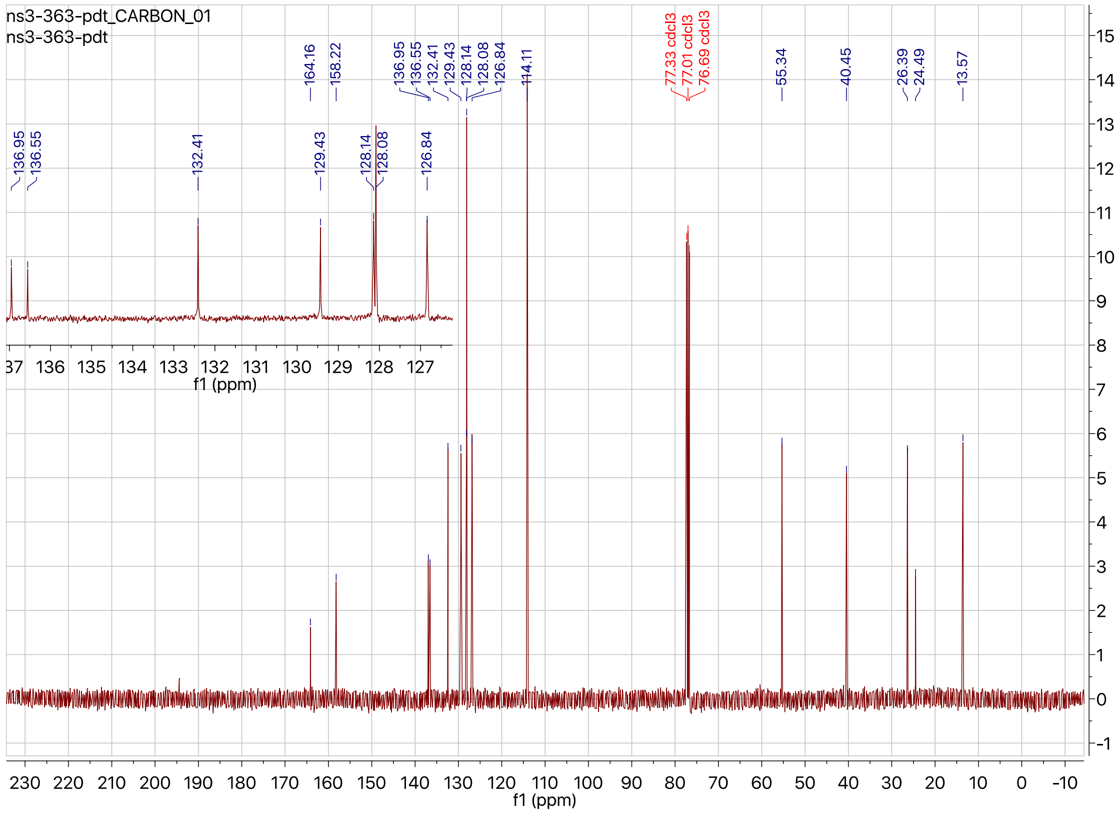
ns3-363-pdt_CARBON_01
ns3-363-pdt
f1 (ppm)

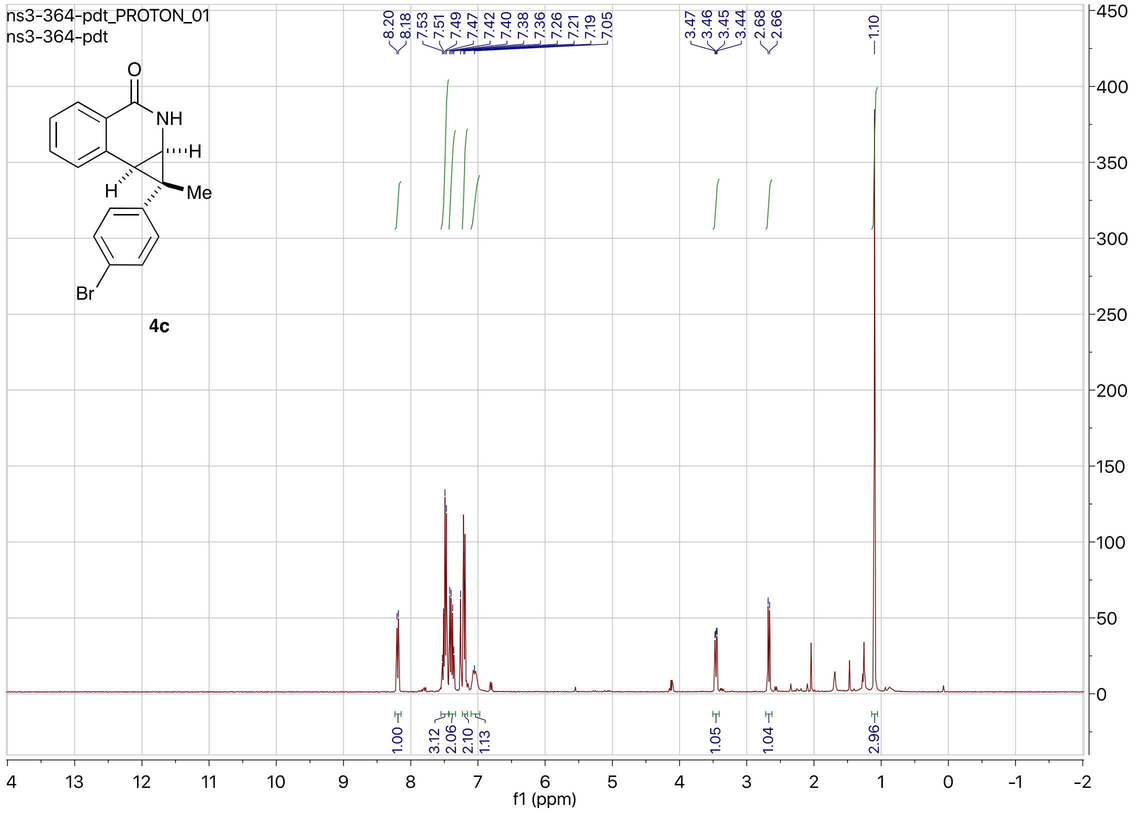
ns3-364-pdt_PROTON_01
ns3-364-pdt
4c
f1 (ppm)

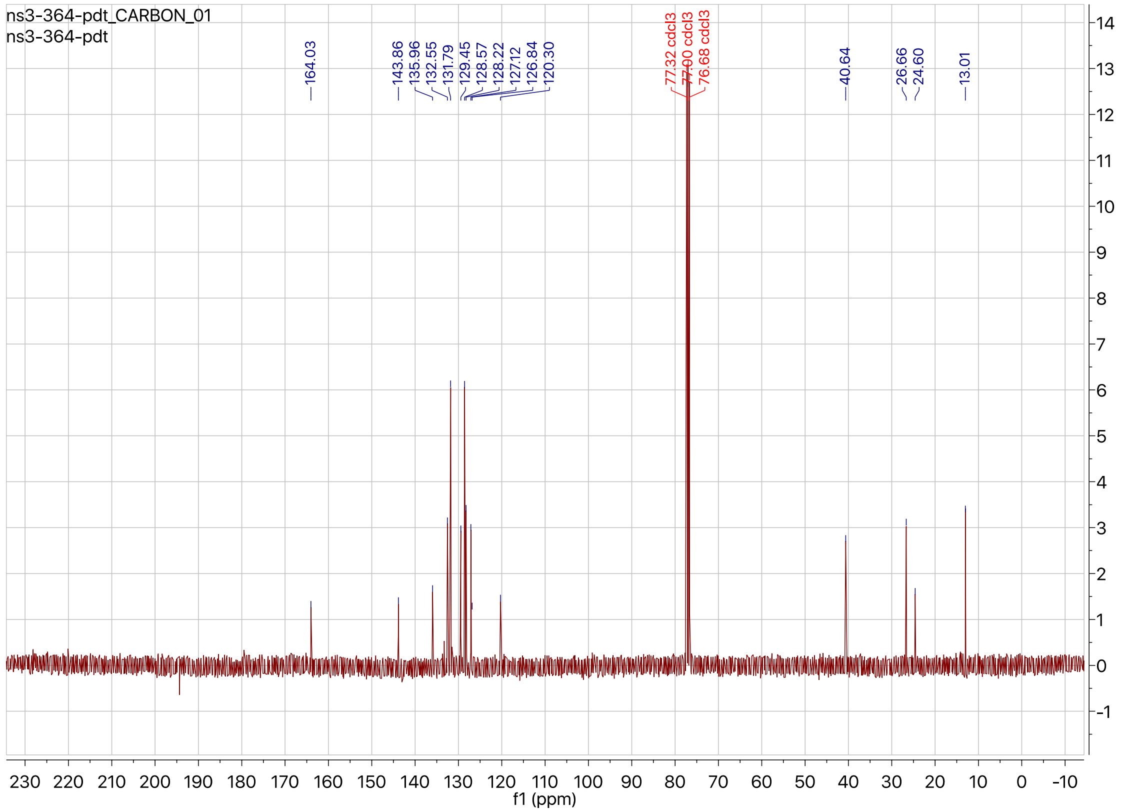
ns3-364-pdt_CARBON_01
ns3-364-pdt
f1 (ppm)

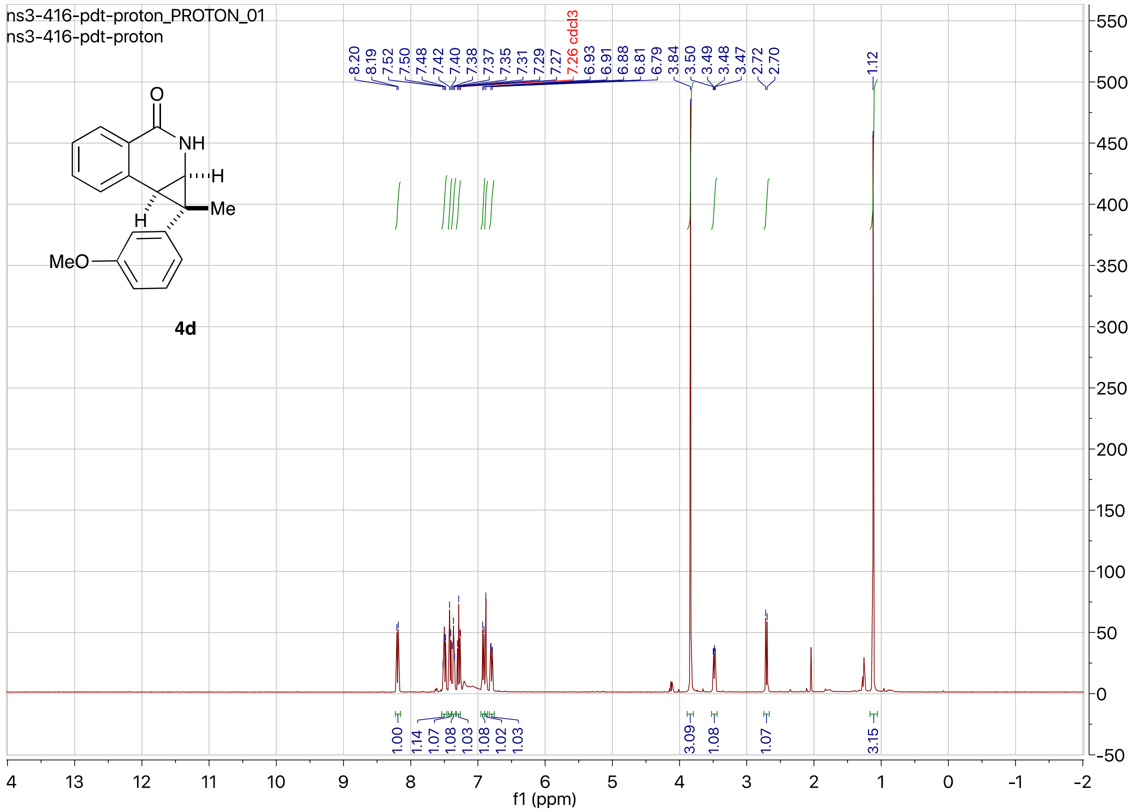
ns3-416-pdt-proton_PROTON_01
ns3-416-pdt-proton
8.20 8.19 7.52 7.50 7.48 7.42 7.40 7.38 7.37 7.35 7.31 7.29 7.27 7.26 cdcl3 6.93 6.91 6.88 6.81 6.79 3.84 3.50 3.49 3.48 3.47 2.72 2.70 1.12
O
NH
H
Me
H
MeO
4d
1.00 1.14 1.07 1.08 1.03 1.08 1.02 1.03 3.09 1.08 1.07 3.15
f1 (ppm)

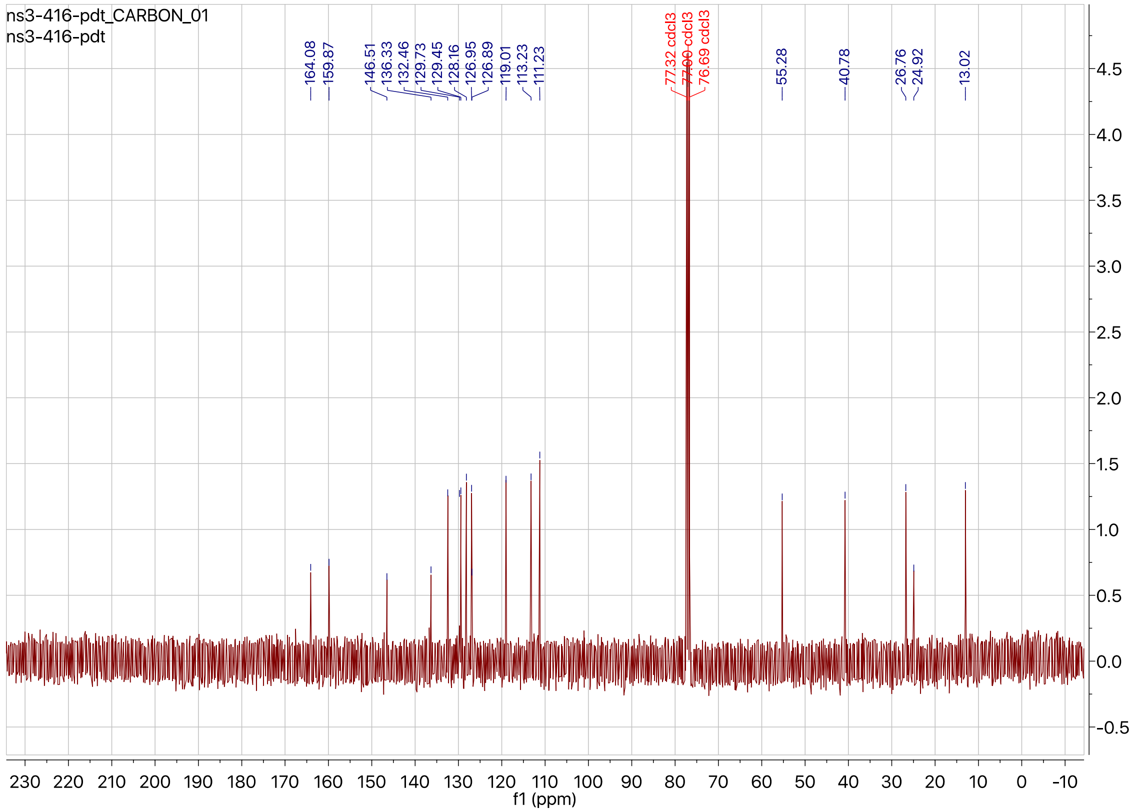
ns3-416-pdt_CARBON_01
ns3-416-pdt
164.08 159.87 146.51 136.33 132.46 129.73 129.45 128.16 126.95 126.89 119.01 113.23 111.23
77.32 cdcl3 77.00 cdcl3 76.69 cdcl3
55.28 40.78 26.76 24.92 13.02
f1 (ppm)

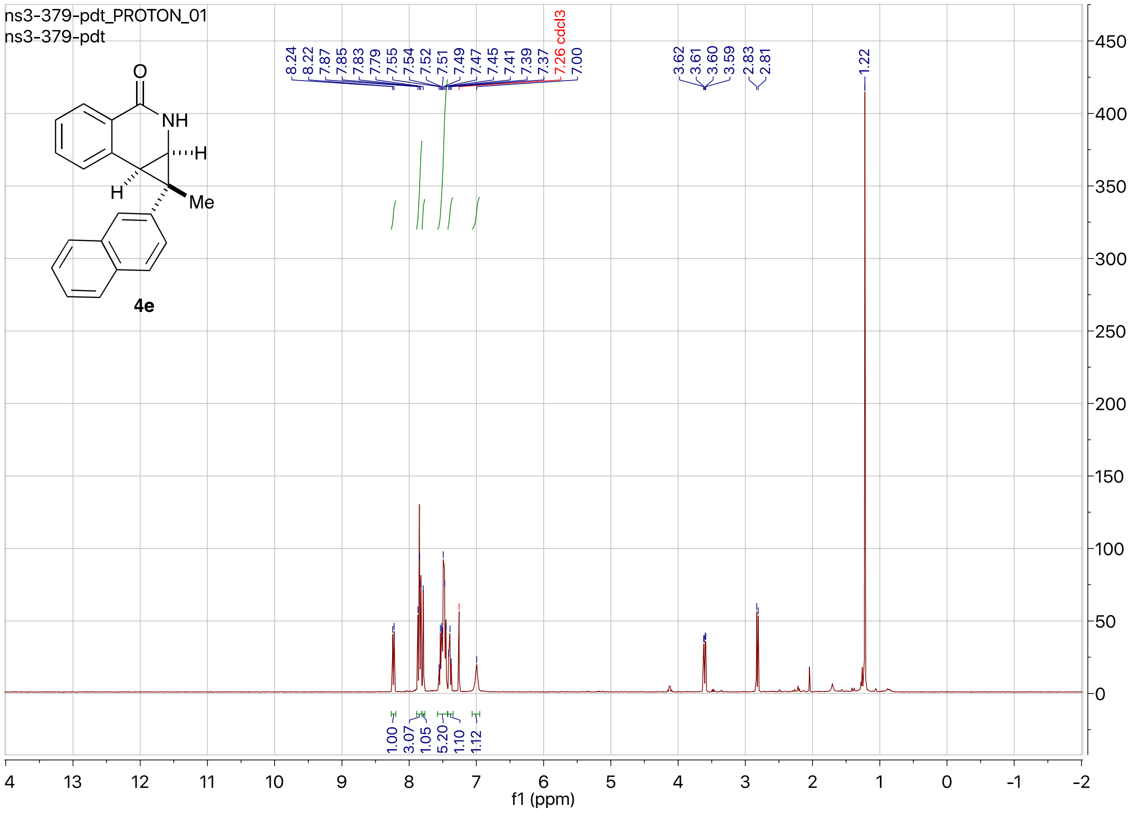
ns3-379-pdt_PROTON_01
ns3-379-pdt
8.24 8.22 7.87 7.85 7.83 7.79 7.55 7.54 7.52 7.51 7.49 7.47 7.45 7.41 7.39 7.37 7.26 cdcl3 7.00
3.62 3.61 3.60 3.59 2.83 2.81
1.22
O
NH
H
H
Me
4e
1.00 3.07 1.05 5.20 1.10 1.12
f1 (ppm)

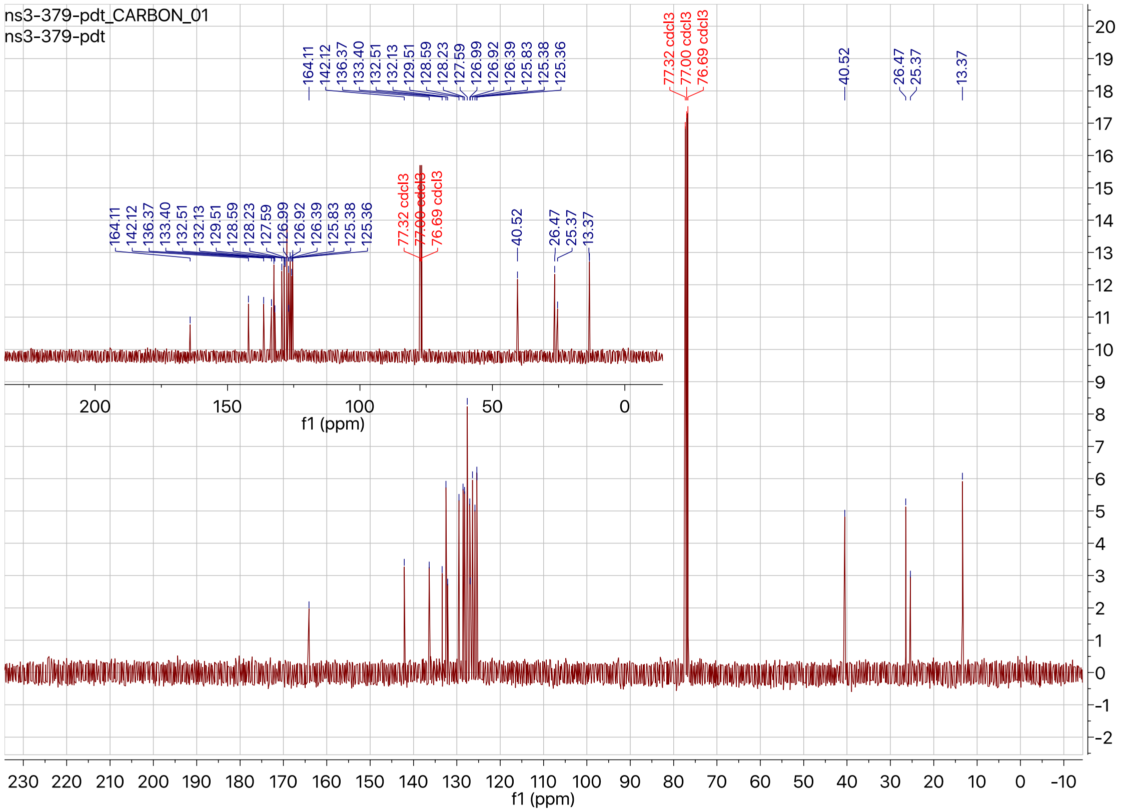
ns3-379-pdt_CARBON_01
ns3-379-pdt
164.11 142.12 136.37 133.40 132.51 132.13 129.51 128.59 128.23 127.59 126.99 126.92 126.39 125.83 125.38 125.36
77.32 cdcl3 77.00 cdcl3 76.69 cdcl3
40.52
26.47 25.37
13.37
f1 (ppm)

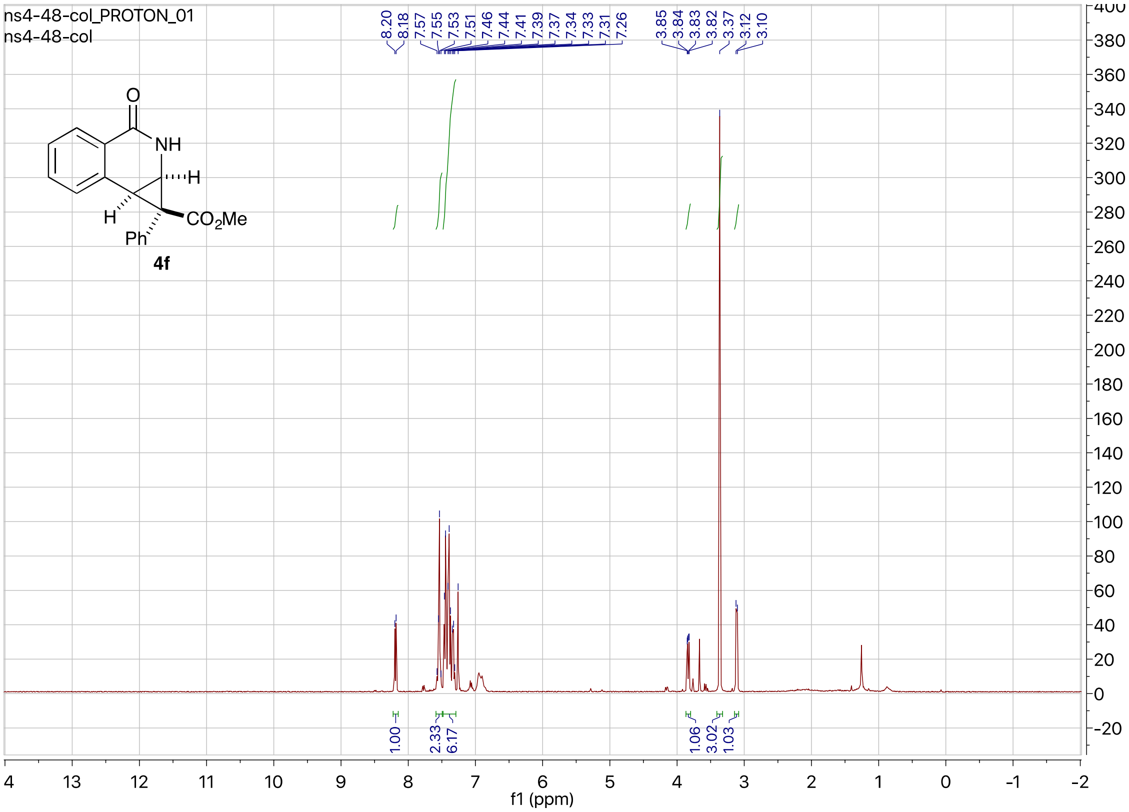
ns4-48-col_PROTON_01
ns4-48-col
8.20
8.18
7.57
7.55
7.53
7.51
7.46
7.44
7.41
7.39
7.37
7.34
7.33
7.31
7.26
3.85
3.84
3.83
3.82
3.37
3.12
3.10
O
NH
H
H
CO2Me
Ph
4f
1.00
2.33
6.17
1.06
3.02
1.03
f1 (ppm)

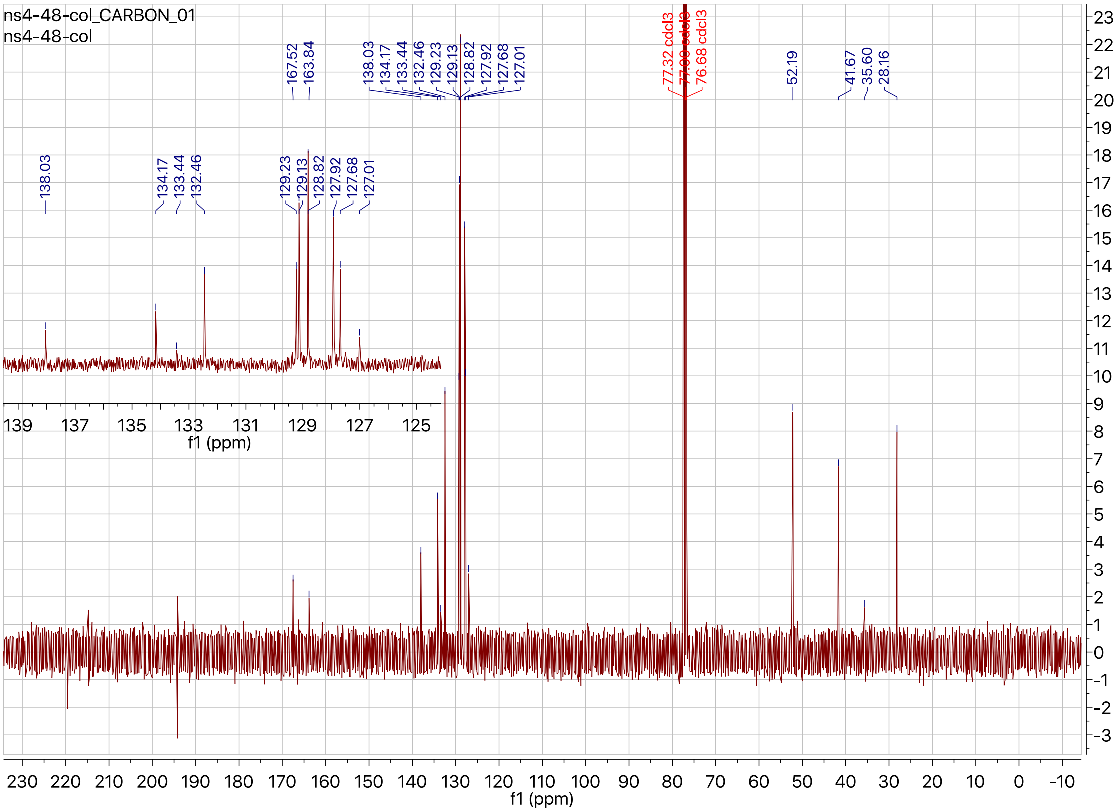
ns4-48-col_CARBON_01
ns4-48-col
167.52
163.84
138.03
134.17
133.44
132.46
129.23
129.13
128.82
127.92
127.68
127.01
77.32 cdcl3
77.00 cdcl3
76.68 cdcl3
52.19
41.67
35.60
28.16
f1 (ppm)

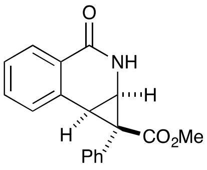

O
NH
H
H
CO2Me
Ph

4f

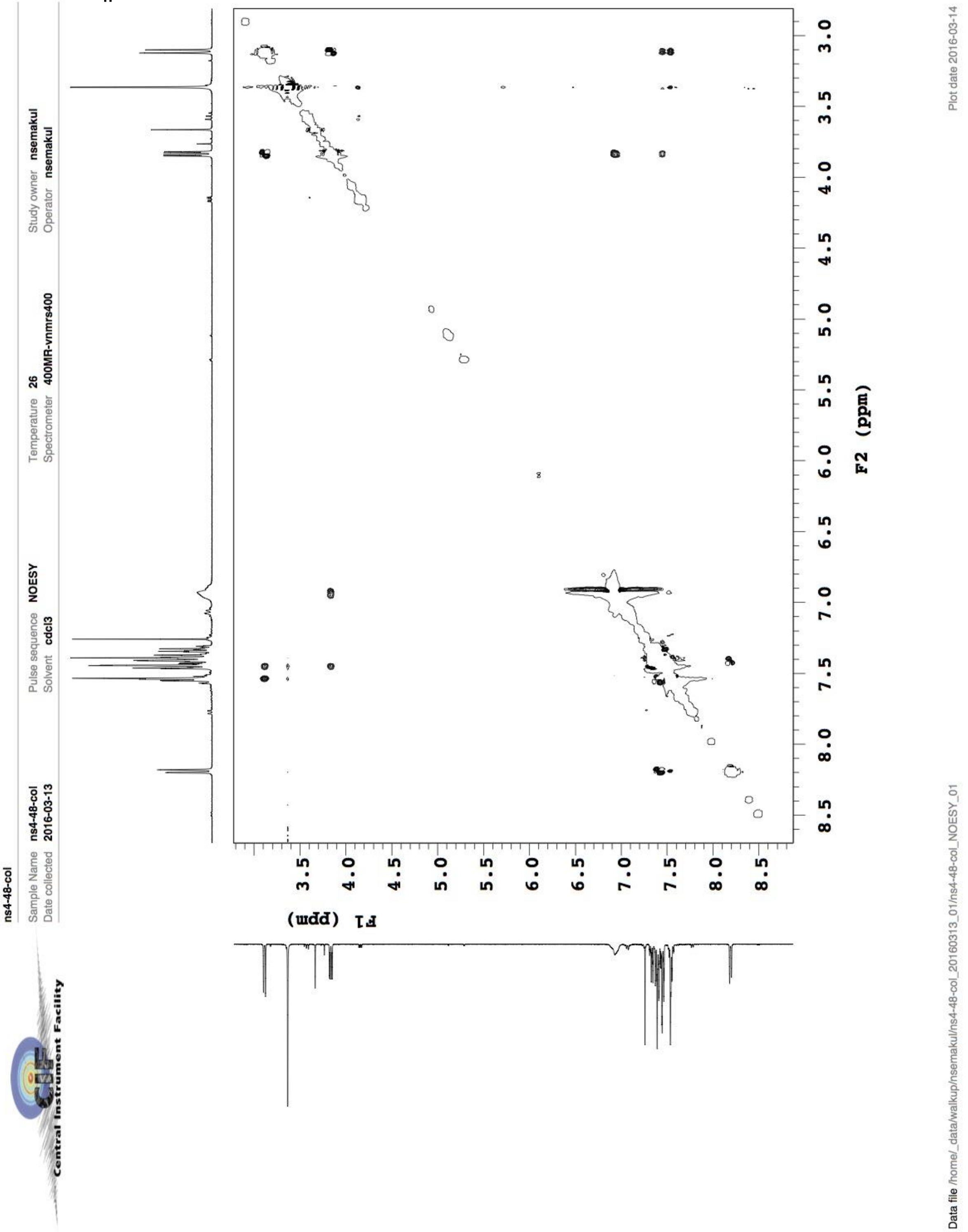

ns4-48-col
Sample Name ns4-48-col
Date collected 2016-03-13
Pulse sequence NOESY
Solvent cdcl3
Temperature 26
Spectrometer 400MR-vnmrs400
Study owner nsemakul
Operator nsemakul
F2 (ppm)
F1 (ppm)
Central Instrument Facility
Plot date 2016-03-14
Data file /home/_data/walkup/nsemakul/ns4-48-col_20160313_01/ns4-48-col_NOESY_01

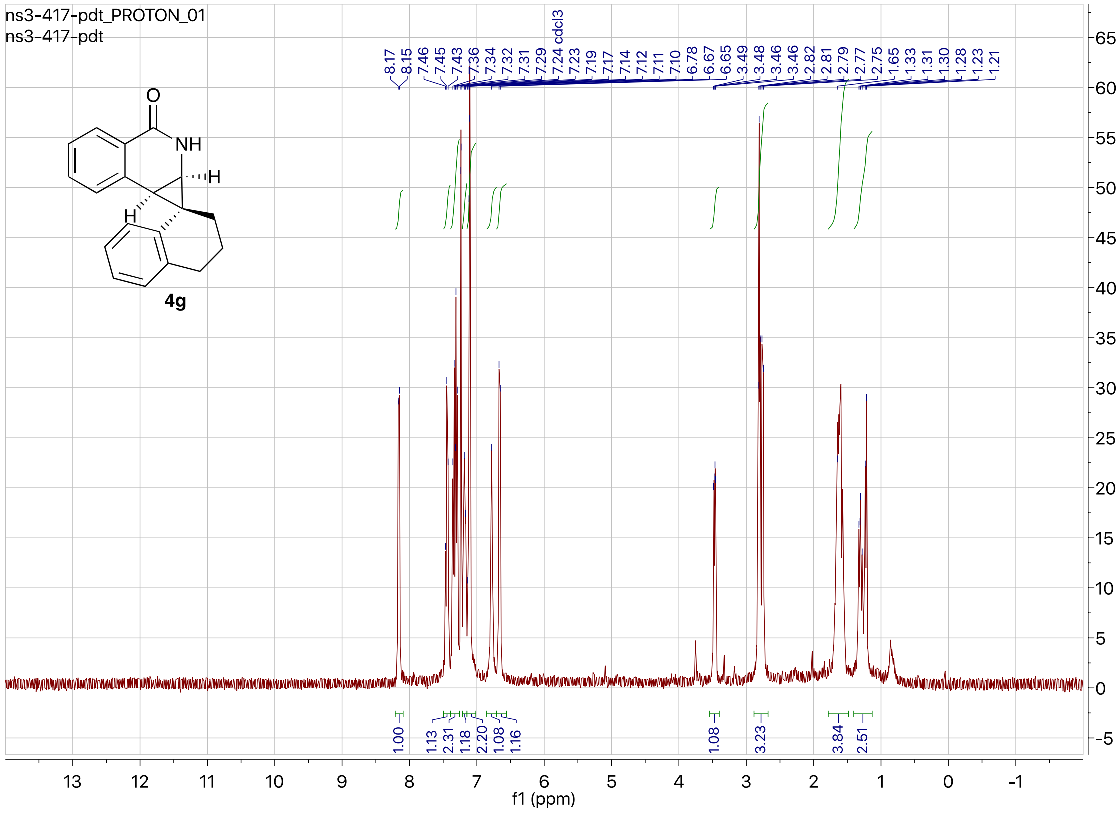
ns3-417-pdt_PROTON_01
ns3-417-pdt
8.17
8.15
7.46
7.45
7.43
7.36
7.34
7.32
7.31
7.29
7.24 cdcl3
7.23
7.19
7.17
7.14
7.12
7.11
7.10
6.78
6.67
6.65
3.49
3.48
3.46
3.46
2.82
2.81
2.79
2.77
2.75
1.65
1.33
1.31
1.30
1.28
1.23
1.21
O
NH
H
H
4g
1.00
1.13
2.31
1.18
2.20
1.08
1.16
1.08
3.23
3.84
2.51
f1 (ppm)

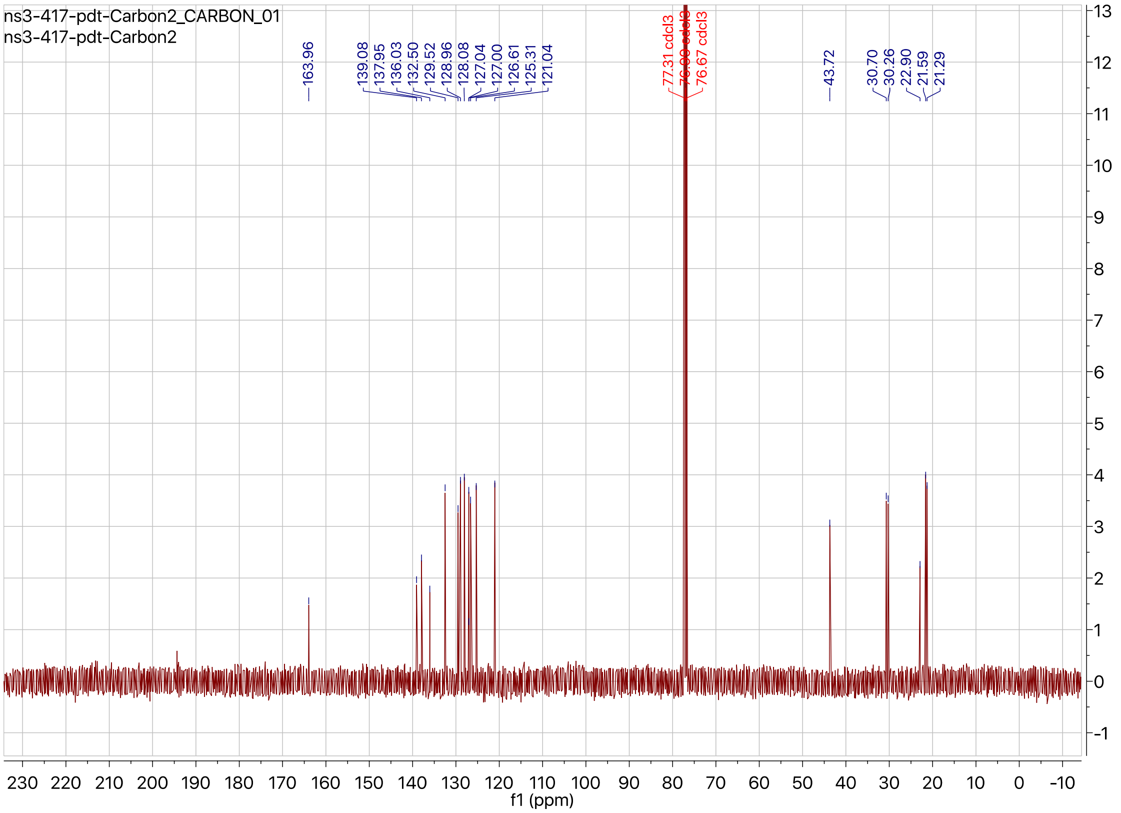
ns3-417-pdt-Carbon2_CARBON_01
ns3-417-pdt-Carbon2
163.96
139.08
137.95
136.03
132.50
129.52
128.96
128.08
127.04
127.00
126.61
125.31
121.04
77.31 cdcl3
76.67 cdcl3
43.72
30.70
30.26
22.90
21.59
21.29
f1 (ppm)

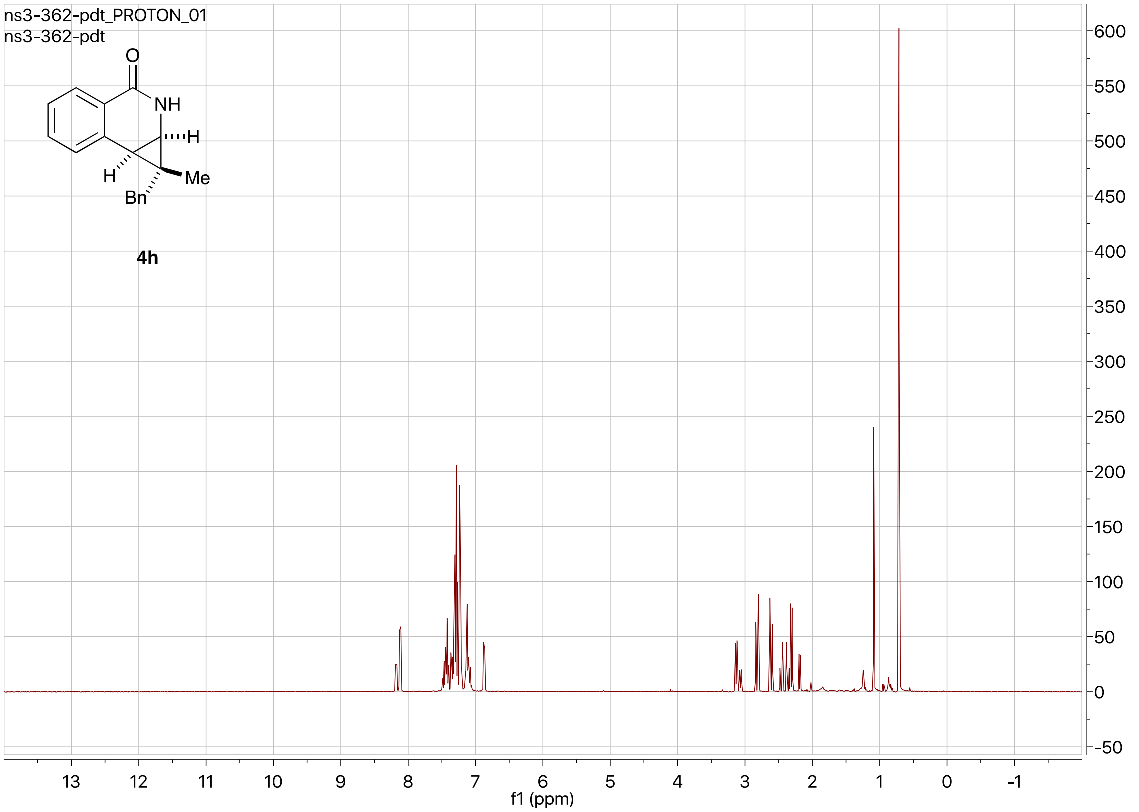
ns3-362-pdt_PROTON_01
ns3-362-pdt
O
NH
H
H
Me
Bn
4h
f1 (ppm)

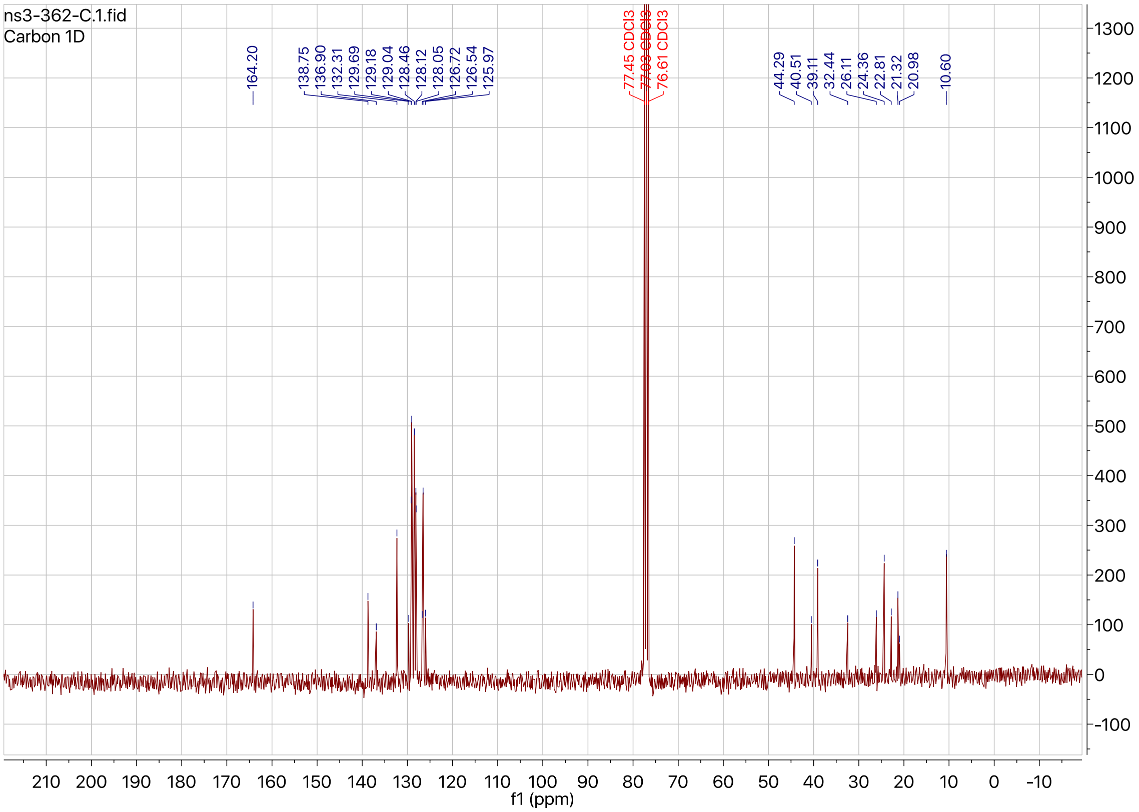
ns3-362-C.1.fid
Carbon 1D
164.20
138.75
136.90
132.31
129.69
129.18
129.04
128.46
128.12
128.05
126.72
126.54
125.97
77.45 CDCl3
76.61 CDCl3
44.29
40.51
39.11
32.44
26.11
24.36
22.81
21.32
20.98
10.60
f1 (ppm)

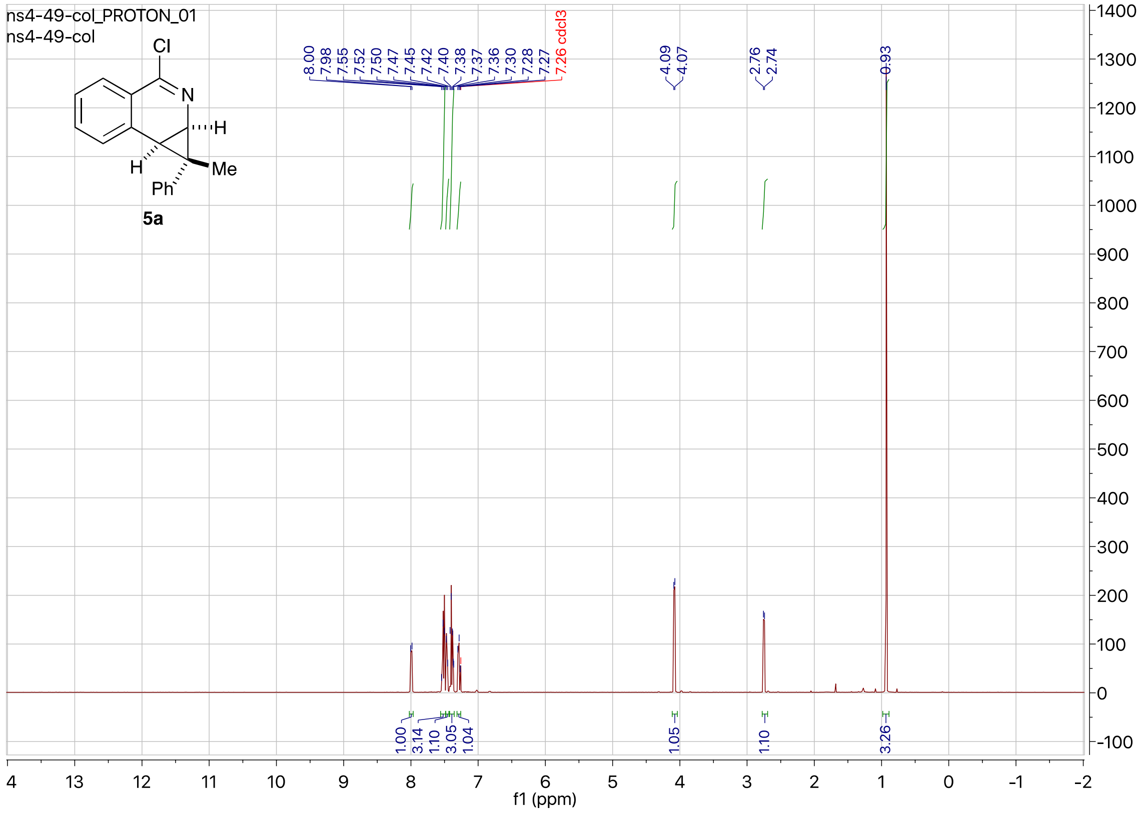
ns4-49-col_PROTON_01
ns4-49-col
Cl
N
H
H
Me
Ph
5a
8.00 7.98 7.55 7.52 7.50 7.47 7.45 7.42 7.40 7.38 7.37 7.36 7.30 7.28 7.27
7.26 cdcl3
4.09 4.07
2.76 2.74
0.93
1.00 3.14 1.10 3.05 1.04
1.05
1.10
3.26
f1 (ppm)

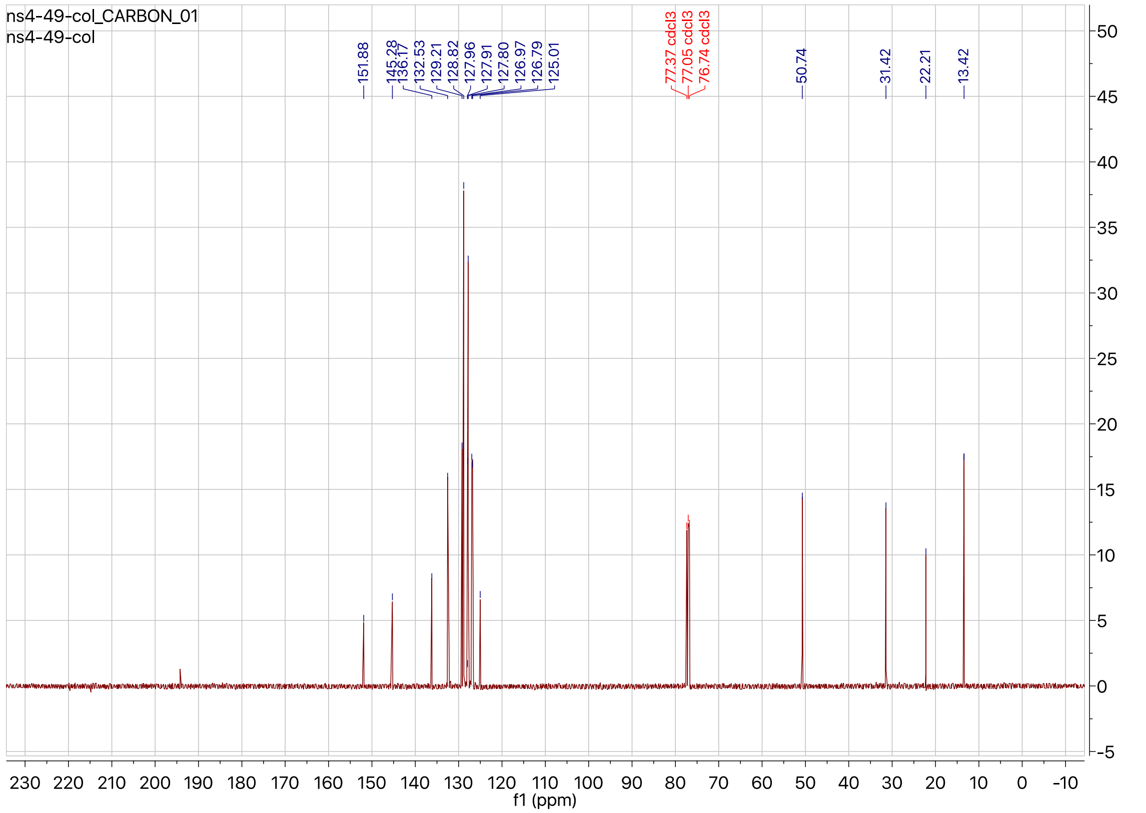
ns4-49-col_CARBON_01
ns4-49-col
151.88 145.28 136.17 132.53 129.21 128.82 127.96 127.91 127.80 126.97 126.79 125.01
77.37 cdcl3 77.05 cdcl3 76.74 cdcl3
50.74
31.42
22.21
13.42
f1 (ppm)

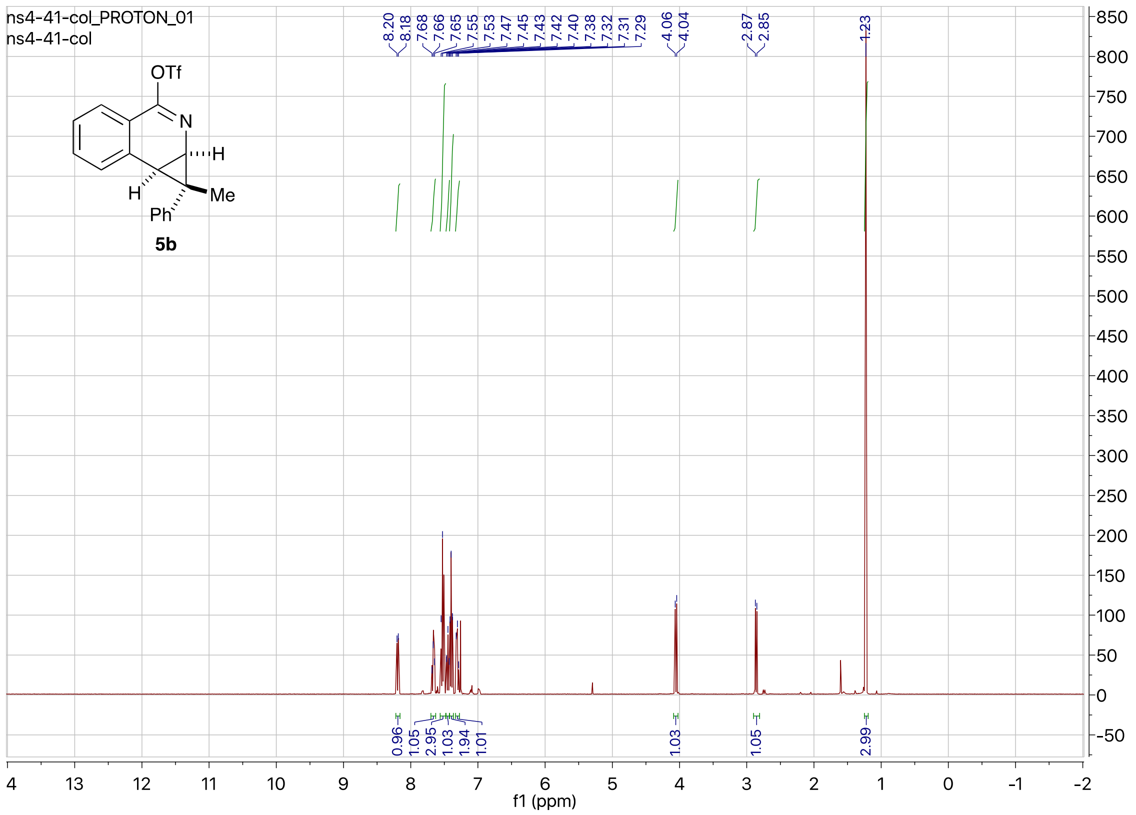
ns4-41-col_PROTON_01
ns4-41-col
OTf
N
H
H
Me
Ph
5b
8.20 8.18 7.68 7.66 7.65 7.55 7.53 7.47 7.45 7.43 7.42 7.40 7.38 7.32 7.31 7.29 4.06 4.04 2.87 2.85 1.23
0.96 1.05 2.95 1.03 1.94 1.01 1.03 1.05 2.99
f1 (ppm)

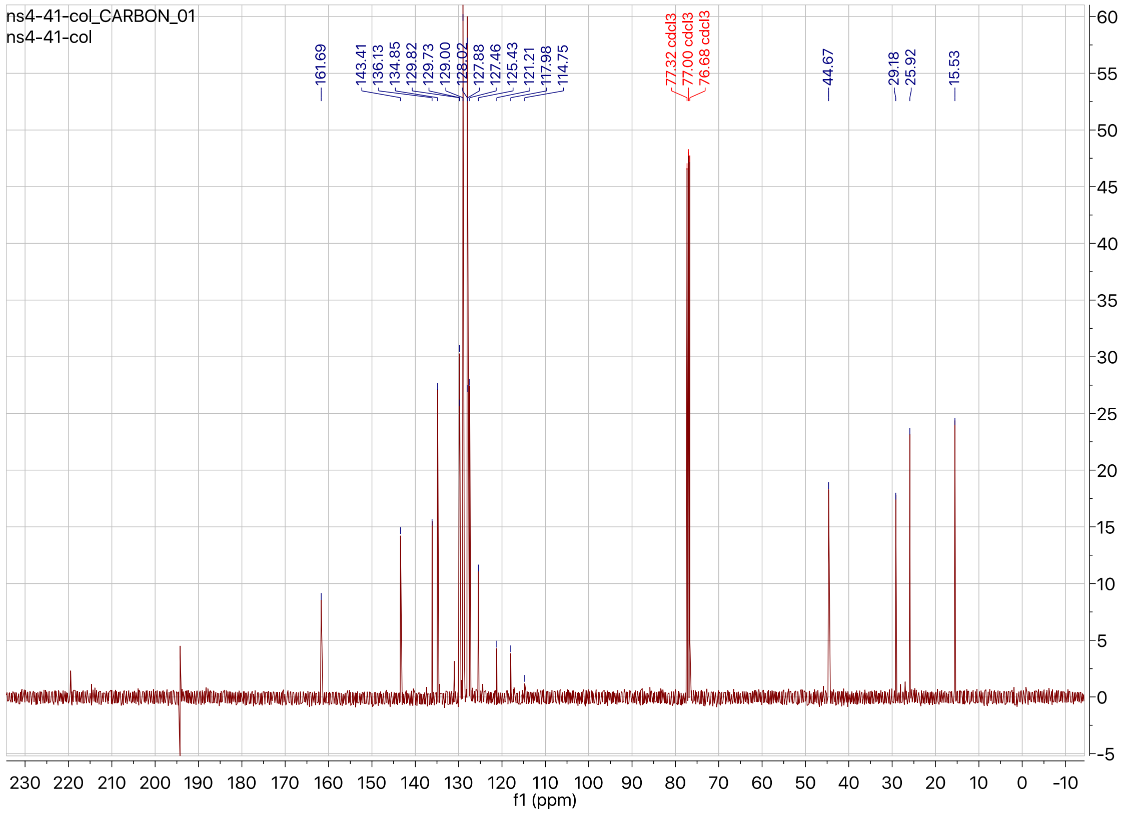
ns4-41-col_CARBON_01
ns4-41-col
161.69
143.41 136.13 134.85 129.82 129.73 129.00 128.02 127.88 127.46 125.43 121.21 117.98 114.75
77.32 cdcl3 77.00 cdcl3 76.68 cdcl3
44.67
29.18 25.92
15.53
f1 (ppm)

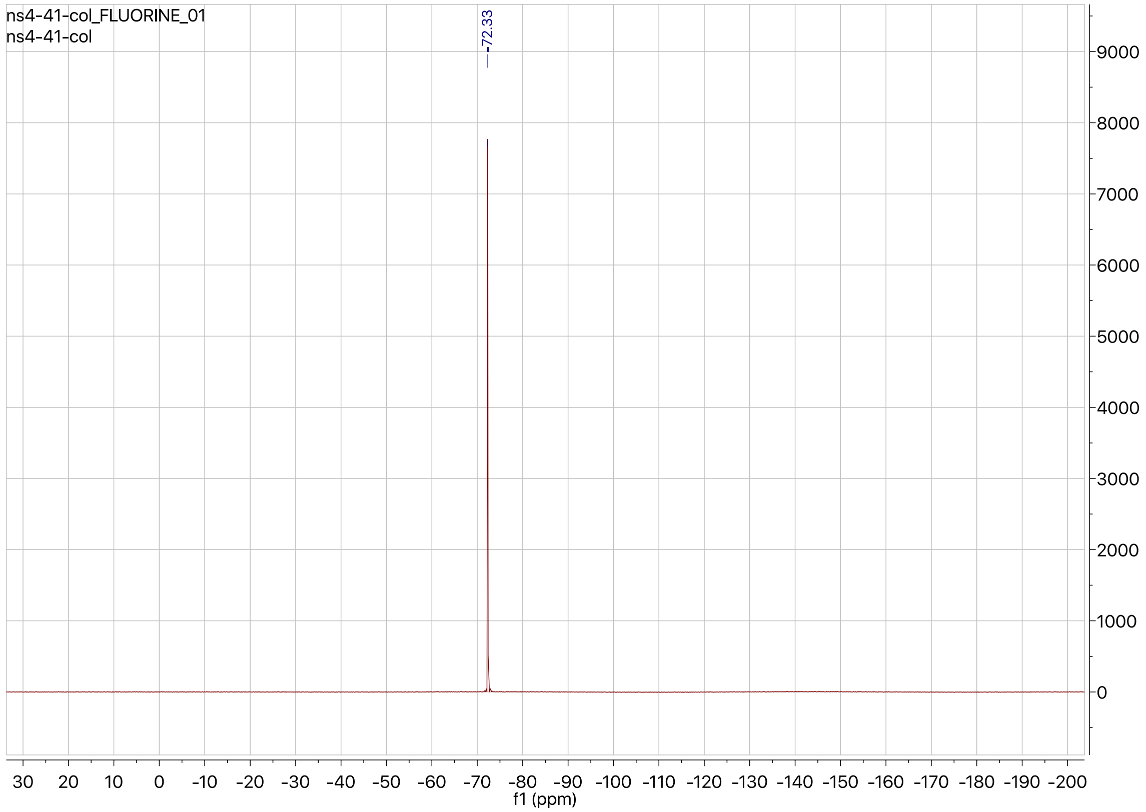

ns4-41-col_FLUORINE_01
ns4-41-col
-72.33
9000
8000
7000
6000
5000
4000
3000
2000
1000
0
30 20 10 0 -10 -20 -30 -40 -50 -60 -70 -80 -90 -100 -110 -120 -130 -140 -150 -160 -170 -180 -190 -200
f1 (ppm)
